# Unusual Rearrangement of a 1,8-Naphthalene Derivative

**DOI:** 10.1021/acs.joc.5c00021

**Published:** 2025-04-03

**Authors:** Asmaa Habib, Estela Sánchez-Santos, Irene Boya del Teso, José J. Garrido-González, Francisca Sanz, Luis Simón, Joaquín R. Morán, Ángel L. Fuentes de Arriba

**Affiliations:** † Organic Chemistry Department, 16779 University of Salamanca, Plaza de los Caídos s/n, Salamanca 37008, Spain; ‡ X-Ray Diffraction Service, University of Salamanca, Plaza de los Caídos s/n, Salamanca 37008, Spain; § Chemical Engineering Department, University of Salamanca, Plaza de los Caídos s/n, Salamanca 37008, Spain

## Abstract

The steric strain between nitro and carboxylic acid groups in an 8-nitro-1-naphthoic acid derivative is able to unexpectedly disrupt the aromaticity of the naphthalene core under mild reaction conditions. The addition of H_2_O to the aromatic ring of a highly strained naphtho oxazinium intermediate induces the fragmentation of a Csp^2^–Csp^2^ bond, with a concomitant rearrangement to yield a conjugated aldehyde. Key intermediates have been characterized, and the X-ray structure of the derivative has been obtained. Density functional theory (DFT) studies were performed to confirm the proposed mechanism.

## Graphical abstract

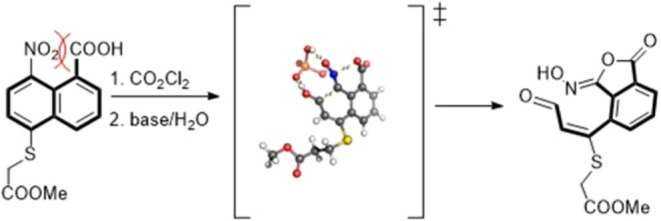

## Introduction

1,8-Napthalene derivatives are strained compounds due to the steric hindrance between substituents in 1,8-positions, which produce unusual physical properties. Anderson studied in 1975 that the rotational barrier energy of some naphthalene derivatives with *peri* interactions strongly depends on the planarity or tetrahedral character of the substituents.^1^ In some cases, the short distance between *peri* substituents facilitates a chemical reaction between them (Figure 1A–C). Dünitz observed that 8-(dimethylamino)-1-naphthylmethyl ketone has the dimethylamino group tilted toward the ketone group to exploit the binding interactions provided by the Burgi–Dünitz trajectory. In this way, a dihedral angle of 116.65° instead of 120° was observed (Figure 1A).^2^ This explains the reactivity observed by Wallis in many 1,8-substituted naphthalenes,^3^ with the reaction between dimethylamino-1-naphthaldehyde and benzoyl chloride, which produces the dihydro benzolinium compound, being one of the most remarkable reactions, as shown in Figure 1B.^3e^ Recently, Lectka has studied how the NH stretching frequency and the ^1^H–^19^F coupling constants of NH···F interactions are affected in 1,8-disubstituted naphthalenes (Figure 1C).^4^ In addition, the strain experienced by the groups in the *peri* position indicates that some reactions proceed easily due to the activation caused by the steric repulsion (Figure 1D–F). A highlighting example is the reaction reported by Priestap of 8-nitro-1-naphthoic acid, which yields a naphthoxazole when treated with Zn and acetic acid (Figure 1D),^5^ releasing the steric strain between the groups in 1- and 8-positions. The chemical properties are also strongly influenced; 1,8-bis­(dimethylamino) naphthalene (proton sponge) is a stronger base (p*K*_a_ = 12.1) than the corresponding dimethylaniline (p*K*_a_ = 5.1) since amine protonation releases a part of the steric strain between the two amino groups (Figure 1E).^6^ Recently, Nishiwaki has studied how bulky substituents in the *peri* position are able to distort the coplanarity of the naphthalene ring^7^ and affect the isomerization of bromine substituents (Figure 1F).^8^

**Figure 1:**
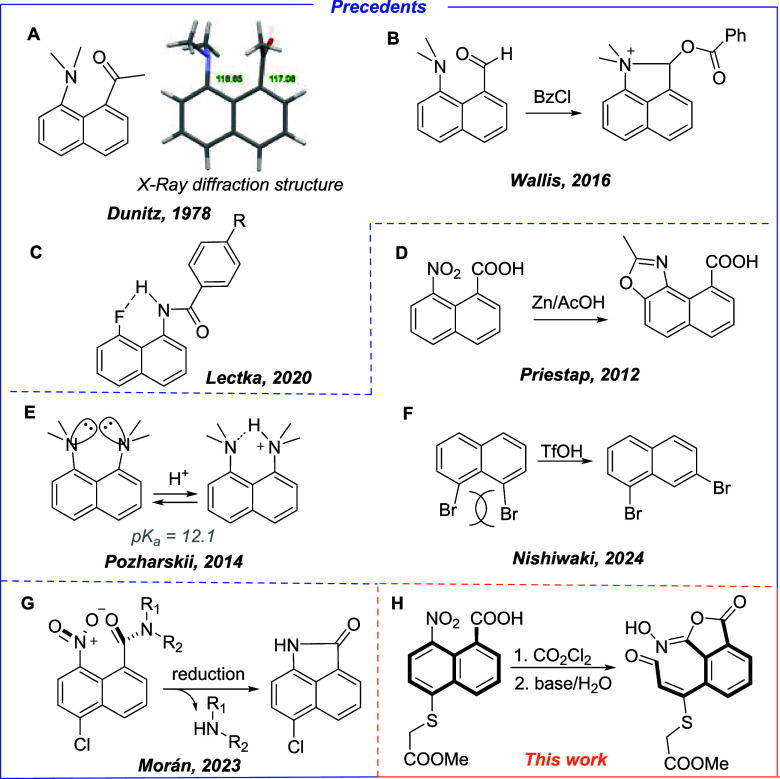
(A–G) Unusual chemical reactivity and physical properties of 1,8-disubstituted naphthalenes. (H) Unexpected rearrangement obtained in this work.

Recently, 5-chloro-8-nitro-1-naphthoyl (NNap) has been proposed by our group as an amine-protecting group, which easily liberates the amine and generates a lactam (Figure 1G).^9^ The nitro and amide groups suffer steric hindrance, and they are twisted from the naphthalene plane, as has been already revealed from X-ray diffraction studies. After the reduction of the nitro group to the amino group, the proximity with the amide facilitates the formation of the lactam with the concomitant release of the amine and the disappearance of the steric tension.

We envisioned that NNap could be used as a drug carrier by anchoring a drug to the carboxylic acid group, which could be released under reducing conditions. To overcome solubility issues, the chlorine atom in C-5 was substituted by a water-soluble moiety. Therefore, a thioglycolic fragment was included in the molecule, as shown in Figure 1H. Nevertheless, an attempt to couple the naphthalene fragment with a nitrogenated mustard (a well-known intercrossing DNA molecule) under Schotten–Baumann conditions led to the formation of an unexpected aldehyde in good yield (Figure 1H) with the concomitant fragmentation of the naphthalene ring. Intriguingly, this reaction was not observed when the Cl atom was placed in the C-5 position of the naphthalene ring. Herein, we explain how this reaction occurs and propose a mechanism to explain this unusual rearrangement.

## Results and Discussion

Compound **1** (Scheme 1) was synthesized following the literature procedure.^9^ Then, the thioglycolic acid fragment was introduced by nucleophilic aromatic substitution and the carboxylic acid attached to the thioglycolic moiety was selectively esterified with MeOH and SOCl_2_. The putative acid chloride of compound **2** was prepared by dissolving it in oxalyl chloride (see the Supporting Information (SI)). Then, Schotten–Baumann conditions with Na_2_CO_3_ and EtOAc were used to anchor nitrogen mustard **HCl·3**. However, the expected amide was only found at trace amounts, and an unexpected compound was obtained instead in high yield. The same outcome was obtained when the reaction was performed in wet dichloromethane (DCM) with excess of neutral bis­(2-chloroethyl)­amine or a biphasic reaction mixture with CH_2_Cl_2_ and aqueous phosphate buffer (Scheme 1 and SI).

**Scheme 1:**
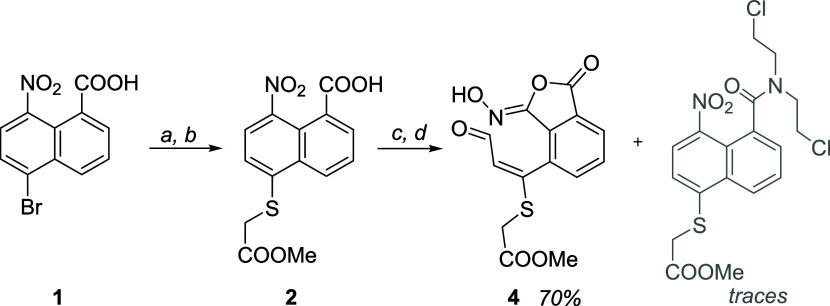
Synthesis of Aldehyde **4** from Reported Compound **1** ^a^

a Reaction conditions: (a) 5 equiv of thioglycolic acid sodium salt, 5 equiv of KOH, dimethyl sulfoxide (DMSO)/dimethylformamide (DMF)/H_2_O (25/5/2), 2 M, 5 °C, 89%; (b) 0.3 equiv of SOCl_2_, MeOH/tetrahydrofuran (THF) (3/1), 0.1 M, 88%; (c) 10 equiv of CO_2_Cl_2_, 0.02 equiv of NBu_4_Cl, 40 °C, 99%; and (d) 4.5 equiv of bis­(2-chloroethyl)­amine (**3**), wet DCM, 0.026 M, −30 °C, 70% or DCM/saturated aqueous KH_2_PO_4_.

Positive electrospray high-resolution mass spectrometry (HRMS) showed a peak at 344.0193 corresponding to the molecular formula C_14_H_11_NNaO_6_S^+^ ([M + Na]^+^). This formula is consistent with the protonated aldehyde **4** and also with a structural isomer of the starting naphthoic acid **2**. The presence of the aldehyde functional group in the rearranged compound **4** was supported by the IR spectrum with characteristic absorptions for aldehydes at 2850 and 2754 cm^–1^. The stretching carbonyl band is hidden under two other carboxyl bands at 1730 cm^–1^. The ^1^H NMR spectrum also supports the aldehyde functional group due to the presence of a 7.9 Hz doublet at 9.08 ppm coupled with an olefinic proton, and the ^13^C NMR spectrum presents a carbonyl carbon CH at 188.9 ppm. The presence of the aldehyde is also supported by the formation of a hemiacetal in CD_3_OD after adding a trace of *p*TsOH to a compound **4** CD_3_OD solution, showing two doublets at 6.00 and 4.50 ppm with *J* = 6.7 Hz. The unusual (*Z*)-3-(hydroxyimino)­isobenzofuran-1­(3*H*)-one (phthaloxime) structure is consistent with the carboxyl carbon at 163.2 ppm and the imino carbon at 145.7 ppm. Heteronuclear multiple bond coherence (HMBC) CIGAR long-range proton–carbon correlations are also consistent with these assignations since the hydroxyimino proton singlet at 11.83 ppm showed a three-bond correlation with the hydroxymino carbon in the DMSO-*d*_6_ spectrum at 145.8 ppm, while the aromatic *ortho* proton at 8.07 ppm showed the corresponding correlation with the carboxyl carbon (Figure S8). All of the other correlations are in agreement with the proposed structure (see the SI). An interesting fact is the splitting of the methylene protons in the ^1^H NMR spectrum as an AB system at 3.95 and 3.89 ppm. This fact seems not to agree with a planar structure but can be explained by the presence of atropoisomers. Modeling studies predict a rotational barrier for this structure around 20 kcal/mol (Figure 2), high enough to prevent rotation in the NMR scale.

**Figure 2:**
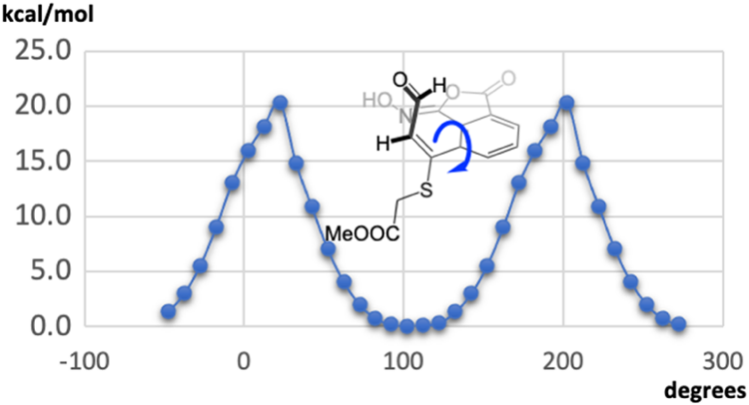
Modeling studies of the phthaloxime aldehyde showing the twisting between the aromatic ring and the substituent supporting the aldehyde.

The phthaloxime **I** ^10^ (Scheme 2) has been known for a long time, as the result of the reaction of phthalic anhydride and *O*-benzyl hydroxylamine, followed by hydrogenolysis. It has been reported that this structure is in equilibrium with the hydroxyphthalimide structure **II** through a deep red anion intermediate formed under basic conditions (Scheme 2, top) and assignation of the right structure has been a matter of considerable debate.^11^

**Scheme 2:**
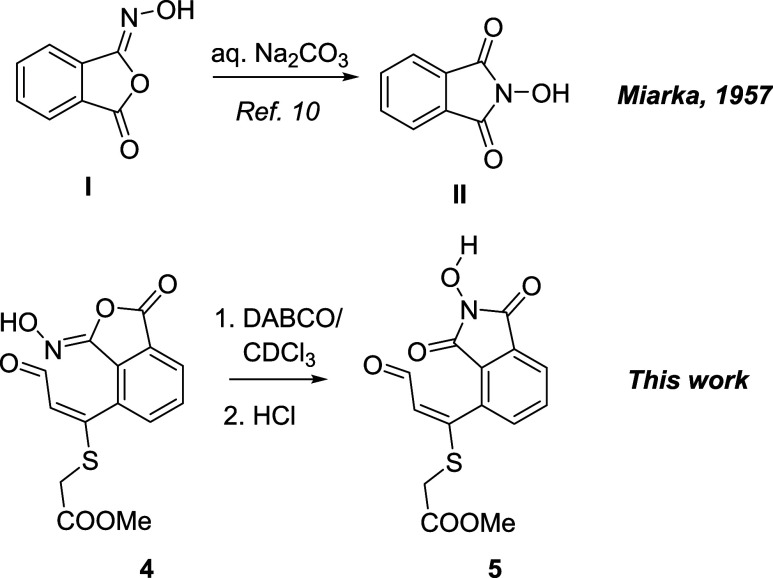
(Top) Rearrangement of (*Z*)-3-(Hydroxyimino)­isobenzofuran-1­(3*H*)-one into *N*-Hydroxyphthalimide under Basic Conditions Reported in Literature and (Bottom) Rearrangement Found with Aldehyde **4**

In our case, treatment of a CDCl_3_ solution of the phthaloxime **4** with 1,4-diazabicyclo[2.2.2]­octane (DABCO) indeed yielded a deep red solution, from which, after acidulation with aqueous 2 M HCl, the hydroxyphthalimide compound **5** was obtained (Scheme 2, bottom). The IR spectrum agreed with the hydroxyphthalimide structure with a characteristic band at 1787 cm^–1^.^12^ The most convincing fact is the appearance of the two carboxyl carbons in the ^13^C NMR spectrum at 162.4 and 163.0 ppm, while the imino carbon at 145.8 ppm has disappeared. All of the other spectroscopic properties agree with the structure (SI); therefore, in this case, there is no structure ambiguity between both compounds.

We were unable to obtain suitable crystals from the previous aldehydes **4** or **5** for X-ray analysis; therefore, the 2,4-dinitrophenyl hydrazones of both compounds were prepared with 2,4-dinitrophenyl hydrazine in THF. Both aldehydes rendered the same dinitrophenyl hydrazone, which is a deep red crystalline compound from which the X-ray analysis was possible. The structure shown in Figure 3 corresponds to the hydroxyphthalimide compound **6** since the phthaloxime rearranges under these conditions to the hydroxyphthalimide.

**Figure 3:**
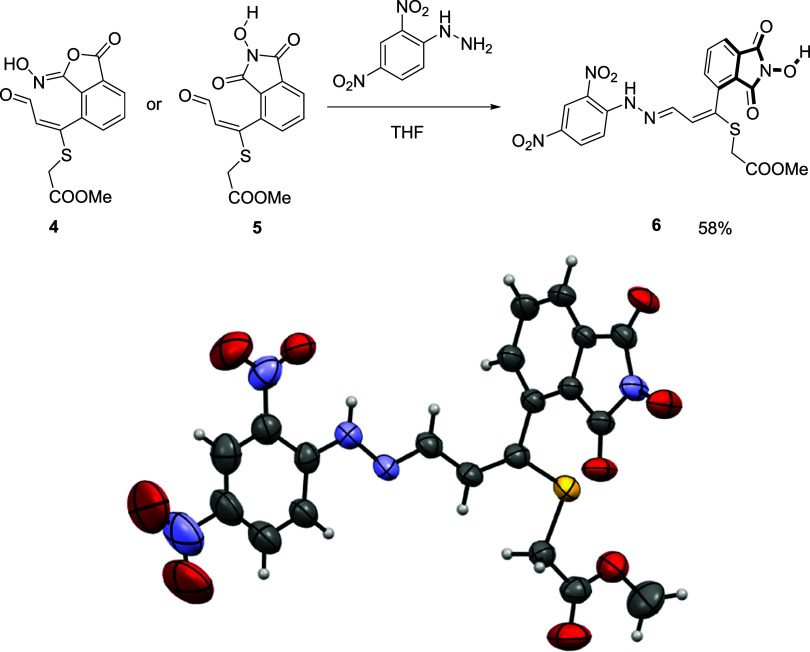
(Top) Synthesis of compound **6** from either **4** or **5**. (Bottom) Oak Ridge thermal ellipsoid plot (ORTEP) diagram of compound **6**. Displacement ellipsoids are drawn at the 50% probability level. Hydrogen atoms are shown as spheres of arbitrary radius.

This structure is in good agreement with the previous modeling of the phthaloxime aldehyde **4**, showing an almost 90° torsion angle between the hydroxyphthalimide aromatic ring and the substituent. This fact supports the presence of atropoisomers in these compounds.

A comparison of the ^13^C NMR spectra of the hydrazone with both previous aldehydes confirms the structure of the phthaloxime for the reaction of acylnitronium chloride since the hydrazone lacks the 145.8 ppm carbon from the imine, while it shows the two carboxyl carbons at 162.9 and 163.7 ppm. This result shows that the phthaloxime rearranges to the hydroxyphthalimide in the presence of dinitrophenyl hydrazine.

After elucidating the structure of the aldehyde **4**, we aimed at investigating the mechanism to explain the unexpected formation of this compound from 1,8-disubstituted naphthalene **2**. We were suspicious of the reaction between compound **2** and oxalyl chloride to generate the acid chloride since the reaction of an acid chloride with a secondary amine should obviously yield an amide. In order to explain the low yield of the amide, we discarded the acid chloride as the intermediate, and we proposed a naphtho oxazinium chloride **7** instead (Scheme 3) as the reaction product of the acid **2** with oxalyl chloride. This intermediate offers a plausible explanation to justify the preference for the nucleophilic attack on the C-7 position of the naphthalene ring instead of the carbonyl group of an acid chloride.

**Scheme 3:**
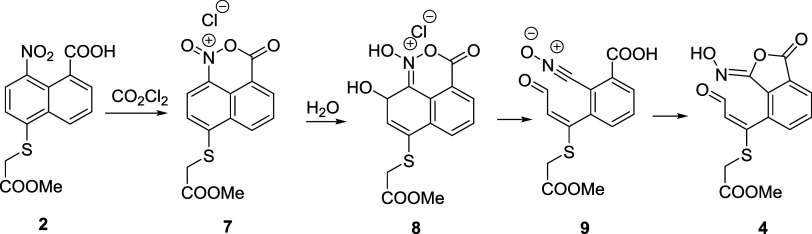
Key Intermediates Proposed for the Rearrangement of 1,8-Disubstituted Naphthalene **2**

Although the naphtho oxazine chloride **7** is a highly unstable compound, it has been possible to carry out a reasonable characterization. High-resolution mass spectrometry shows a peak at 304.0273 corresponding to the molecular formula C_14_H_10_NO_5_S^+^. The lack of the chloride atom in the molecular formula ruled out the acid chloride structure. The IR spectrum showed a broad band at 1728 cm^–1^, inconsistent with an acid chloride, which is expected to absorb around 1800 cm^–1^. ^13^C NMR showed two carboxyl signals at 168.8 and 167.7 ppm, consistent with the expected chemical shifts for the naphtho oxazinium carbon and the ester group.

Although the addition of water to the naphthalene skeleton seems to be a counterintuitive reaction, as it should yield a large loss of aromatic energy, a good precedent can be found in the formation of the naphtho oxazole compound reported by Priestap (Figure 1D). In fact, these compounds also show interesting pharmaceutical and biological properties since they have properties similar to those of aristolochic acids, which have been proposed to act through a nitrenium ion to couple with DNA through the addition of the amino group of a DNA nucleobase (Scheme 4). However, no fragmentation was reported in this case.

**Scheme 4:**
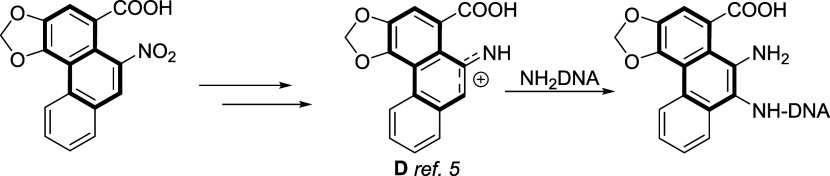
Naphtho Oxazole Derivatives with Cytotoxic Activity and the Proposed Nitrenium Intermediate Which Reacts with the Amino Group of DNA Nucleobases

Trying to show that indeed the naphtho oxazinium chloride **7** undergoes nucleophilic attack in C-7, this compound was treated at 0 °C with potassium acetate. Two doublets with *J* = 5.2 Hz grow up during the reaction in the ^1^H NMR spectrum in CDCl_3_ at 6.67 and 6.15 ppm, consistent with the addition of the acetate to the C-7 naphthalene ring. However, the compound is unstable, and further characterization has not been possible. We have been able to perform this study more easily with the chloride derivative in position 5 instead of the thioglycolic fragment since a simple washing of the potassium acetate salt with cold water and evaporation of the solvent yielded a crystalline compound that was easier to handle. The structure of the acetate is shown in Scheme 5. The physical properties are in good agreement with those of the addition compound. The HRMS spectrum shows a peak at 315.9976 corresponding to the formula C_13_H_8_O_5_N^35^Cl^23^Na, which matches the proposed acetate. This substitution is known to take place in the presence of oxalyl chloride, as already reported in the literature.^9^ The ^1^H NMR spectrum showed two doublets at 6.72 and 6.41 ppm corresponding to the proton geminal to the acetate group and the vinyl proton, respectively, confirming again the addition of the acetate (SI). This easy nucleophilic addition to the naphthalene C-7 of the dioxo naphtho oxazine may be an interesting clue for the addition of nucleic bases to aristolochic acids as an alternative to the reported nitrenium cation intermediates.

**Scheme 5:**
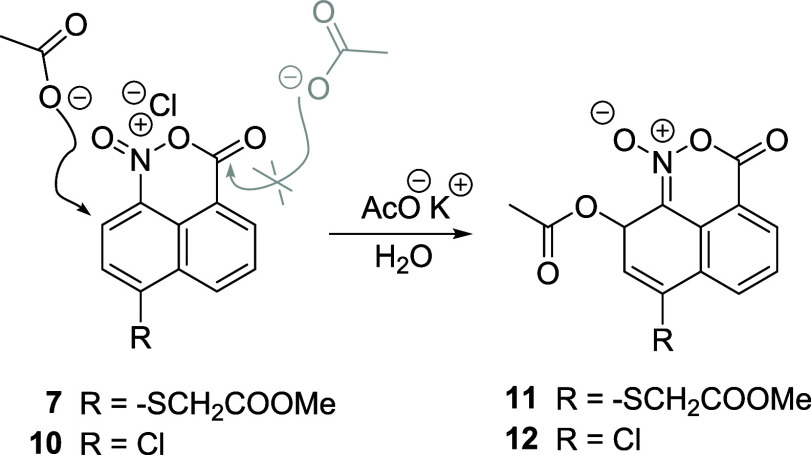
Addition of Potassium Acetate to the Naphthalene Compounds **7** and **10** in the C-7 Position

As acetate **12** cannot undergo further reaction to the aldehyde, its hydrolysis to the alcohol was undertaken; however, hydrolysis of the naphtho oxazinium was obtained instead.

Therefore, in order for compound **7** to generate the observed product **4**, the addition of water to C-7 should yield an alcohol (**8**), which can evolve to an aldehyde with concomitant ring opening and formation of the nitrile oxide **9** (Scheme 3, **7** → **8** → **9**). A precedent to this reaction can be found in the Mukaiyama nitrile oxide synthesis from nitroderivatives and isocyanates (Scheme 6).^13^

**Scheme 6:**
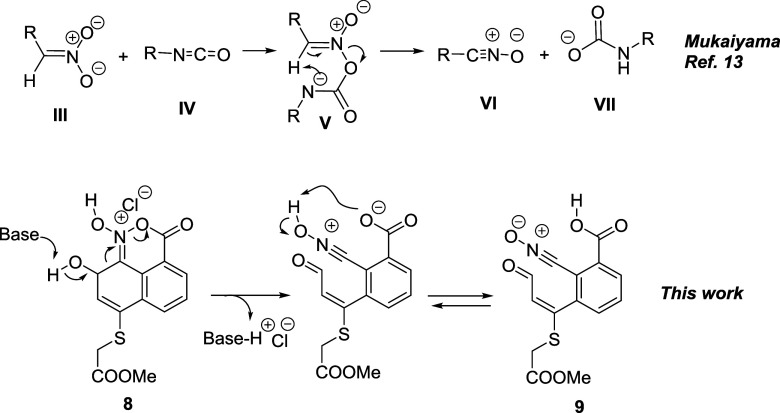
(Top) Mukaiyama Nitrile Oxide Synthesis and (Bottom) Rearrangement Observed in This Work

The formation of the acyl nitronate is similar in both cases. While in the Mukaiyama reaction a proton is abstracted to yield the nitrile oxide and a carbamate, in our case it is the C–C fragmentation which provides the pair of electrons to promote the carboxylate elimination, yielding in this process an aldehyde and a nitrile oxide.

Nitrile oxides are highly reactive species, which readily undergo 1,3 dipolar additions.^14^ All attempts to trap this intermediate, even with tetracyanoethylene, were unsuccessful, probably because the intramolecular reaction with the carboxylic group in C-1 is faster (Scheme 3, **9** → **4**), and the phthalooxime **4** is formed.

To shed light into this unusual rearrangement, density functional theory (DFT) calculations (see details in the SI section) were performed to understand the reaction mechanism and, especially, the preference for the addition of H_2_O to the C-7 position of the naphtho oxazinium cation instead of the carboxylic C carbon. To simplify the calculations, the 2-thioacetate group was replaced by a methylsulfane. Dihydrogen phosphate (H_2_PO_4_^–^) and dimethylamine were studied as possible basic catalysts. Solvent effects (chloroform) were included by an implicit solvation model, and in the reaction catalyzed by dimethylamine, the chlorine counteranion was not included (the net charge of the molecule was +1 in the calculations), while in the case of the H_2_PO_4_^–^-catalyzed reaction, this group was used as a counteranion (the net charge was 0). An exhaustive search of TS structures with different orientations of the reacting groups was performed, although some of these orientations collapsed during the optimization to the same structure.

For the reaction catalyzed by dimethylamine, addition to the carboxylic C atom was favored (a ≈2.0 kcal/mol energy difference between the most stable TSs, a 95:5 ratio considering all TSs, see the SI). In the case of the reaction catalyzed by H_2_PO_4_^–^, the major product corresponds to the addition to C-7 (energy difference of ≈2.5 kcal/mol between most stable TSs, 97:3 ratio, Scheme 7). These results agree with the experimental observations. Not surprisingly, analogue calculations for the addition of one H_2_O molecule to the acyl chloride show a much larger preference for the addition on the carboxylic C atom (≈8.0 kcal/mol), confirming that the formation of the naphtho oxazinium chloride **A** is necessary to justify the reactivity observed (Scheme 7).

**Scheme 7:**
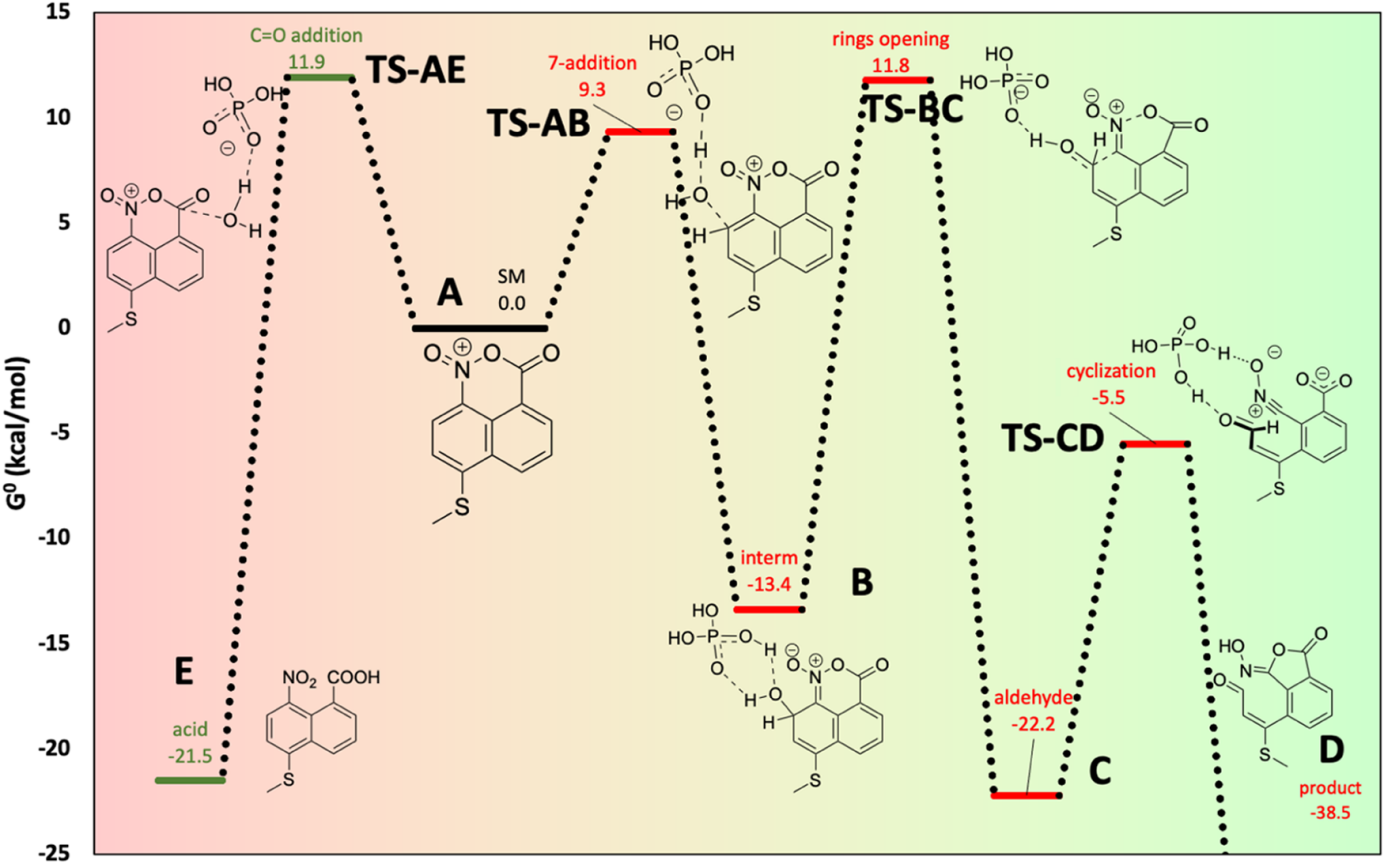
Computer Reaction Energy Profile for the Pathway through the Lowest-Energy Transition-State Structure Leading to the Rearranged Aldehyde (Green Background) and the Competing Hydrolysis Reaction (Red Background)

The reaction continues with the opening of two of the six-membered rings of the intermediate **C**: the C7–C8 bond and the N–O bonds are broken to generate the aldehyde and hydroxynitrile groups **C**. TS structures corresponding to this step (**TS-BC**), catalyzed by an additional dihydrogen phosphate group, were also found. The hydroxy group can adopt a *pseudoaxial* or *pseudoequatorial* position, but in the last case, it is oriented in an almost planar *s-trans* conformation with respect to the conjugated double bond, an effect that probably contributes to stabilize the equatorial TS structure. The reaction is concerted, but shows some degree of asynchronicity: the N–O distance is 2.5 Å, while the C–C distance is elongated to 1.9 Å (Figure 4). Finally, the reaction proceeds through **TS-CD** and product **D** (corresponding to compound **4**) is formed.

**Figure 4:**
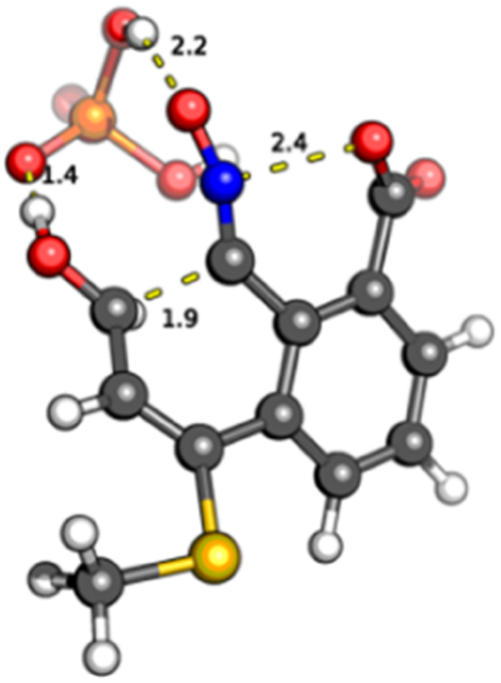
Minimum energy transition state for the H_2_PO_4_^–^-catalyzed fragmentation of C7–C8 and N–O bonds.

Since the addition of a water molecule to C-7 (the preceding step) is reversible, the accumulation of the aldehyde product also requires that the TSs corresponding to this second step are also more stable than the TSs (**TS-AE**) of the H_2_O addition to the carbonyl C atom. Otherwise, the irreversible formation of the aldehyde from the intermediate would be slower than the protonation and elimination of the C-7 OH group and the addition of the resulting water molecule to the carbonyl C atom to yield the competing reaction toward the carboxylate **E**. However, direct comparison of the energies of these TSs is not possible since they have different numbers of atoms; the second-step TSs are deprotonated with respect to the TSs for the addition of H_2_O to the starting materials.

In order to correct this effect, the energies of a neutral phosphoric acid structure and of an H_2_PO_4_^–^ anion were added and subtracted from the energy of the second-step TS structure. Formally, this balances the number of atoms under conditions in which the concentrations of phosphoric acid and dihydrogen phosphate are identical. Curiously, under these assumptions, the energy of the second-step most stable TSs is 2.5 kcal/mol,^15^ higher than the energy of the most stable TSs corresponding to the H_2_O addition to the C-7 atom, and very similar to the energy of the TSs for the addition on the carbonyl C atom, corresponding to the competing path. Under conditions in which the concentration of H_2_PO_4_^–^ is higher (basic p*K*_a_), the formation of the aldehyde should be favored, while, in agreement with experimental results, acid p*K*_a_ should increase the effective barrier of the second step and the hydrolysis should follow the competing path.

## Conclusions

In summary, an unprecedented rearrangement of a 1,8-nitronaphthalene carboxylic acid derivative toward an aromatic ring bearing an aldehyde and a phthaloxime functional group has been reported. The steric hindrance between the nitro and carboxylate groups in the 1,8-positions promotes the formation of a strained naphtho oxazinium intermediate with an unusual reactivity. Addition of water under very mild reaction conditions at 0–20 °C initiates a fragmentation reaction able to break the aromaticity of the naphthalene core. MS and NMR experiments support the intermediates proposed, and the final structure was confirmed by X-ray diffraction analysis of a 2,4-dinitrophenyl hydrazone derivative. DFT studies have been carried out and they support the mechanism proposed. The naphtho oxazinium intermediate could find application as a cytotoxic reagent by its similar properties to aristolochic acids, which are able to react with DNA nucleobases.

## Supplementary Material



## Data Availability

The data underlying this study are available in the published article and its SI.

## Notes

Complete experimental procedures and characterization data for compounds, including ^1^H, ^13^C, 2D NMR, X-ray crystal analysis, crystallographic data for **6** (CCDC 2351359), and modeling studies (PDF)
