# Supplementary material for: Unusual Rearrangement of a 1,8-Naphthalene Derivative
Source: J Org Chem. 2025 Apr 3;90(14):4946–53. doi: 10.1021/acs.joc.5c00021 (PMC12129256; doi:10.1021/acs.joc.5c00021)
Supplement: Supplementary file 1 [file jo5c00021_si_001.pdf]

# Supporting Information

# An unusual rearrangement of a 1,8-naphthalene derivative

Asmaa Habib,[†] Estela Sánchez-Santos,[†] Irene Boya del Teso,[†] José J. Garrido-González,[†] Francisca Sanz,[‡] Luis Simón,*[,‖], Joaquín R. Morán,[†] Ángel L. Fuentes de Arriba*[,†]

[†] Organic Chemistry Department, University of Salamanca, Plaza de los Caídos s/n, Salamanca 37008, Spain. E-mail: angelfuentes@usal.es

[‡] X-Ray Diffraction Service, University of Salamanca, Plaza de los Caídos s/n, Salamanca 37008, Spain

[‖] Chemical Engineering Department, University of Salamanca, Plaza de los Caídos s/n, Salamanca 37008. E-mail: lsimon@usal.es

# Supporting Information

**Table of contents**

1 General information ........................................ S3
2 Synthesis and Characterization ........................................ S5
3 Crystallographic Data ........................................ S11
4 Computacional Studies ........................................ S14
5 NMR Spectra ........................................ S14
6 References ........................................ S75

## 1 General information

Reagents

Reagents were purchased and used without further purification unless otherwise noted. The solvents used were purified and dried following the standard methods. [1]

Purification of reaction crudes

Reactions were monitored by analytical thin layer chromatography using pre-coated aluminium-backed plates (0.2 mm silica gel 60 F254, Merck®) and visualized by UV light. Purification of compounds was performed using silica gel column chromatography (Chromagel 60A SdS. C.C. 70-200 µm) with solvent mixtures of increasing polarity as eluents and crystallization using different solvent mixtures.

Melting Points (m.p.)

Melting points were measured in a Leica Galen III microscope and are reported in ºC.

IR spectroscopy

IR spectra were recorded using a Nicolet IR100 neat or with Nujol as a suspension.

NMR spectroscopy $^{1}H$ and $^{13}C$

$^{1}H$ NMR and $^{13}C$ NMR spectra were recorded at room temperature using a Bruker WP200-SY and a Bruker Avance NEO 400 MHz with a Prodigy CPPBBO BB-H&F z-gradient cryo-probe (400 MHz to $^{1}H$ and 100 MHz to $^{13}C$) spectrometers (NMR Service, NUCLEUS, University of Salamanca). Chemical shifts (δ) are given in ppm with the solvent signal as internal standard unless otherwise stated ($CHCl_3$ 7.26 ppm for $^{1}H$ NMR, $CDCl_3$ 77.0 ppm for $^{13}C$ NMR; $CD_3OD$ 3.31 ppm for $^{1}H$ NMR, $CD_3OD$ 49.0 ppm for $^{13}C$ NMR; DMSO 2.50 for $^{1}H$ NMR, DMSO-$d_6$ 39.52 for $^{13}C$ NMR). [2] Coupling constants ($J$) were reported in Hertz (Hz). The following abbreviations were used to explain the multiplicities: s, singlet; d, doublet; dd, doublet of doublets; t, triplet; m, multiplet. Structural assignments were made with additional information from gCOSY, gHSQC, and gHMBC experiments.

Mass spectrometry

Mass spectra were recorded on Waters ZQ 4000 (quadrupole) and Thermo Q Exactive Focus (orbitrap) spectrometers.

X-ray diffraction studies

Data collection was performed on a Bruker Kappa APEX II diffractometer. For more details, see Section 3. Crystallographic Data.

Modelling studies

Theorical studies were carried out using Gaussian16 software with w-B97XD functional in combination with 6-31+G(d,p) basis set. For more details, see Section 4. Computational studies.

## 2 Synthesis and Characterization

### 5-bromo-1-naphthoic acid

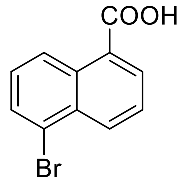


In a round bottom flask (500 ml) 1-naphthoic acid (40 g, 0.23 mol) was suspended in chloroform (400 ml) and acetic acid (40 ml). The reaction was heated to reflux in an oil bath, and bromine (30 ml, 0.59 mol) was slowly added. The brominated compound crystallises after a few minutes and the reaction was heated for further 4 hours in an oil bath to complete the reaction. The reaction mixture was added over water and filtered to yield 5-bromo-1-naphthoic acid as a white solid (50 g, 87% yield).

Spectral properties agree with the literature.[3]

### 5-bromo-8-nitro-1-naphthoic acid (1)

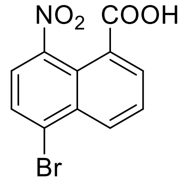


In a two necked round bottom flask with a thermometer 5-bromo-1-naphthoic acid (20 g, 80 mmol) was dissolved in acetic anhydride (200 ml) and *p*TsOH (2 g, 10.5 mmol) was added. The reaction vessel was placed in a water bath with ice and fuming nitric acid (20 ml, 0.44 mol) was carefully added keeping the temperature below 35 ºC. After one hour the reaction mixture was added over 1 liter of water and ice until the acetic anhydride was hydrolyzed. Then the reaction was filtered rendering 5-bromo-8-nitro-1-naphthoic acid as a pale cream solid (18 g, 76% yield).

Spectral properties agree with the literature. [3]

### 5-((carboxymethyl)thio)-8-nitro-1-naphthoic acid

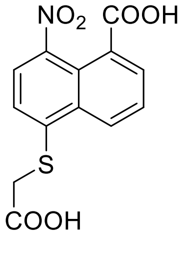


In a round bottom flask thioglycolic acid sodium salt (8 g, 70 mmol), potassium hydroxide (4 g, 0.71 mol) and water (2 ml) were placed and stirred until a homogeneous solution was obtained. Then, 5-bromo-8-nitro-1-naphthoic acid (4 g, 13.5 mmol) was dissolved in DMSO (25 ml) and DMF (5 ml). Both solutions were cooled down to 5 ºC then, the

naphthalene solution was poured over the thioglycolic acid salt, keeping the temperature below 10 ºC. After 10 minutes the reaction was added over 2M aqueous hydrochloric acid (100 ml) and filtered to yield 5-((carboxymethyl)thio)-8-nitro-1-naphthoic acid as a yellow solid (3.7 g, 89% yield).

**m.p.:** >200 ºC; **IR** (nujol, ν in $cm^{-1}$): 3760, 3650, 3617, 1697, 1560, 1521, 1385, 1340, 1262, 1191, 899. **$^1$H NMR** (400 MHz, DMSO-*$d_6$*) δ 8.50 (dd, *J* = 8.5, 0.9 Hz, 1H), 8.22 (d, *J* = 8.2 Hz, 1H), 8.16 (dd, *J* = 7.2, 1.1 Hz, 1H), 7.84 (dd, *J* = 8.5, 7.3 Hz, 1H), 7.62 (d, *J* = 8.3 Hz, 1H), 4.21 (s, 2H). **$^{13}$C{$^1$H} NMR** (101 MHz, DMSO-*$d_6$*) δ 169.6 (C), 168.0 (C), 144.6 (C), 142.7 (C), 131.2 (CH), 129.7 (C), 127.3 (CH), 126.9 (CH), 124.9 (CH), 121.9 (CH), 121.3 (2C), 34.2 ($CH_2$). **HRMS** (ESI) m/z calcd. for $C_{13}H_9O_6N^{23}Na^{32}S$ $[M+Na]^+$ 330.0048, found 330.0043.

**5-((2-methoxy-2-oxoethyl)thio)-8-nitro-1-naphthoic acid (2)**

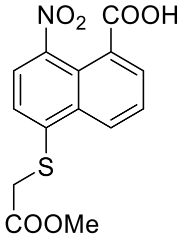


5-((carboxymethyl)thio)-8-nitro-1-naphthoic acid (3.7 g, 12 mmol) was dissolved in MeOH (30 ml) and THF (10 ml). Thionyl chloride (0.25 ml, 3.4 mmol) was added and the reaction was allowed to stand for 1 hour. Evaporation under reduced pressure returned a crude compound which was crystallized from methylene chloride to yield the methyl ester as a yellow solid (3.4 g, 88% yield).

**m.p.:** 170 ºC; **IR** (nujol, ν in $cm^{-1}$): 3903, 3747, 3663, 1761, 1690, 1567, 1288, 1132, 827. **$^1$H NMR** (400 MHz, DMSO-*$d_6$*) δ 8.49 (dd, *J* = 8.6, 1.1 Hz, 1H), 8.19 (dd, *J* = 8.2, 4.3 Hz, 1H), 8.15 (dd, *J* = 7.2, 1.1 Hz 1H), 7.83 (dd, *J* = 8.6, 7.2 Hz, 1H), 7.62 (d, *J* = 8.3 Hz, 1H), 4.30 (s, 2H), 3.66 (s, 3H). **$^{13}$C{$^1$H} NMR** (101 MHz, DMSO-*$d_6$*) δ 168.9 (C), 168.0 (C), 144.8 (C), 142.0 (C), 131.3 (C), 131.2 (CH), 129.7 (C), 127.4 (CH), 127.0 (CH), 124.8 (CH), 122.5 (CH), 121.3 (C), 52.6 ($CH_3$), 33.7 ($CH_2$). **HRMS** (ESI) m/z calcd. for $C_{14}H_{11}O_6N^{23}Na^{32}S$ $[M+Na]^+$ 344.0205, found 344.0199.

**7-((2-methoxy-2-oxoethyl)thio)-1,3-dioxo-1*H*,3*H*-naphtho[1,8-*cd*][1,2]oxazin-1-ium chloride (7)**

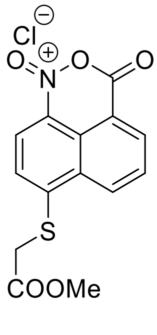

5-((2-methoxy-2-oxoethyl)thio)-8-nitro-1-naphthoic acid (1 g, 3.1 mmol) was suspended in oxalyl chloride (3 ml, 35 mmol) and tetraethyl ammonium chloride (10 mg, 0.06 mmol) was added as a catalyst. The reaction flask was sonicated in an ultrasound bath at 40 ºC for 30 minutes until the starting material was completely dissolved. To the clear solution toluene was added (5 ml) and the solvents were removed under reduced pressure in a water bath at 20 ºC yielding compound **7** as a pale yellow solid (1 g, 99%).

(Melting point and IR were not measured due to the instability of the compound). **$^{1}$H NMR** (400 MHz, $CDCl_3$) δ 8.64 (dd, *J* = 8.6, 1.0 Hz, 1H), 8.36 (dd, *J* = 7.4, 1.0 Hz, 1H), 8.18 (d, *J* = 8.2 Hz, 1H), 7.76 (dd, *J* = 8.6, 7.4 Hz, 1H), 7.61 (d, *J* = 8.2 Hz, 1H), 3.90 (s, 2H), 3.76 (s, 3H). **$^{13}$C{$^{1}$H} NMR** (101 MHz, $CDCl_3$) δ 168.8 (C), 167.7 (C), 144.9 (C), 143.0 (C), 133.1 (CH), 132.7 (C), 130.0 (CH), 126.7 (CH), 125.9 (CH), 124.0 (CH), 121.5 (C), 53.2 ($CH_2$), 35.3 ($CH_3$). **HRMS** (ESI) m/z calcd. for $C_{14}H_{10}NO_5S^+$ ($[M]^+$) 304.0274, found 304.0273.

**Methyl 2-(((*E*)-1-((*Z*)-3-(hydroxyimino)-1-oxo-1,3-dihydroisobenzofuran-4-yl)-3-oxoprop-1-en-1-yl)thio)acetate (4)**

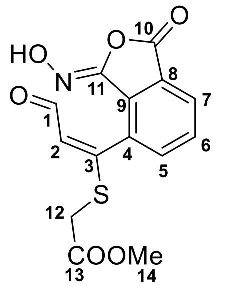


***Procedure A:***

Compound **7** (1 g, 3.1 mmol) was quickly dissolved in methylene chloride (20 ml) and added to a solution of bis(2-chloroethyl)amine (2 g, 14.1 mmol) in an ether solution saturated with water and methylene chloride (100 ml) cooled to -30 ºC. After 30 minutes the reaction mixture was added over aqueous sulfuric acid (2M, 100 ml), separated in a separatory funnel, and the organic phase was washed again with another portion of aqueous sulfuric acid (50 ml) and water (100 ml). Evaporation of the major part of the methylene chloride induced the crystallization of the aldehyde which was filtered under reduced pressure to yield a pale cream solid (700 mg, 70% yield).

***Caution!** Bis(2-chloroethyl)amine must be handled with care due to its toxicity.*

***Procedure B:***

Compound **7** (100 mg, 0.31 mmol) was dissolved in a biphasic mixture of methylene chloride (2 ml) and a saturated aqueous solution of $KH_2PO_4$ (2 ml). The reaction mixture was vigorously stirred for 30 minutes, then it was transferred to a separatory funnel. The organic phase was decantated, dried over anhydrous sodium sulfate, filtered and

evaporated under reduced pressure to yield aldehyde **4** (70% NMR yield) and traces of carboxylic acid **2**.

**m.p.:** 111-113 °C. **IR** (neat, ν in $cm^{-1}$): 3318, 1785, 1734, 1663, 1573, 1525, 1438, 1283, 1137.**$^{1}$H NMR** (400 MHz, DMSO-$d_6$) δ 11.83 (s, OH), 9.07 (d, *J* = 7.9 Hz, 1H(1)), 8.07 (dd, *J* = 7.0, 1.7 Hz, 1H(7)), 7.85 – 7.76 (m, 2H(5,6)), 6.23 (d, *J* = 7.9 Hz, 1H(2)), 3.95 (d, *J* = 16.0 Hz, 1H(12)), 3.89 (d, *J* = 16.2 Hz, 1H(12)), 3.66 (s, 3H(14)). **$^{13}$C{$^{1}$H} NMR** (101 MHz, DMSO-$d_6$) δ 188.9 (CH(1)), 168.2 (C(13)), 163.2 (C(10)), 159.4 (C(3)), 145.8 (C(11)), 136.5 (CH(5)), 132.9 (C(9)), 131.7 (CH(6)), 128.8 (C(8)), 127.0 (C(4)), 126.7 (CH(7)), 124.5 (CH(2)), 52.6 ($CH_3$(14)), 34.0 ($CH_2$(12)). **HRMS** (ESI) m/z calcd. for $C_{14}H_{11}NNaO_6S^+$ ($[M+Na]^+$) 344.0199, found 344.0193.

**Methyl (*Z*)-2-((1-(2-hydroxy-1,3-dioxoisoindolin-4-yl)-3-oxoprop-1-en-1-yl)thio)acetate (5)**

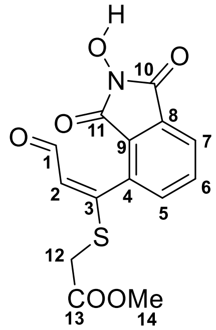


In a round bottom flask methyl 2-(((*E*)-1-((*Z*)-3-(hydroxyimino)-1-oxo-1,3-dihydroisobenzofuran-4-yl)-3-oxoprop-1-en-1-yl)thio)acetate (100 mg, 0.31 mmol) was dissolved in methylene chloride (20 ml) and DABCO (18 mg, 0.16 mmol) was added. After 20 minutes at room temperature, acetic acid was added, and the reaction mixture was chromatographed over silica gel (20 g) eluting with a mixture of methylene chloride/ethyl acetate 4/1 yielding the isomeric aldehyde as a pale cream oil (17 mg, 17% yield).

**$^{1}$H NMR** (400 MHz, $CDCl_3$) δ 9.05 (d, *J* = 7.8 Hz, 1H(1)), 7.90 (dd, *J* = 7.5, 1.0 Hz, 1H(7)), 7.79 (t, *J* = 7.6 Hz, 1H(6)), 7.67 (dd, *J* = 7.8, 0.9 Hz, 1H(5)), 6.26 (d, *J* = 7.8 Hz, 1H(2)), 3.81 (d, *J* = 15.4, 1H(12)), 3.77 (s, 3H(14)), 3.74 (d, *J* = 15.8, 1H(12)). **IR** (neat, ν in $cm^{-1}$): 3494, 3280, 1787, 1723, 1664, 1573, 1314. **$^{13}$C{$^{1}$H} NMR** (101 MHz, $CDCl_3$) δ 188.9 (CH(1)), 168.0 (C(13)), 163.0 (C(10)), 162.4 (C(11)), 159.3 (C(3)), 135.5 (CH(5)), 134.5 (CH(6)), 131.6 (C(4)), 130.0 (C(8)), 126.7 (C(9)), 124.9 (CH(7)), 124.7 (CH(2)), 53.1 ($CH_3$(14)), 34.5 ($CH_2$(12)). **HRMS** (ESI) m/z calcd. for calcd. for $C_{14}H_{11}NNaO_6S^+$ ($[M+Na]^+$) 344.0199, found 344.0194.

**Methyl 2-(((1*Z*,3*Z*)-3-(2-(2,4-dinitrophenyl)hydrazineylidene)-1-(2-hydroxy-1,3-dioxoisoindolin-4-yl)prop-1-en-1-yl)thio)acetate (6)**

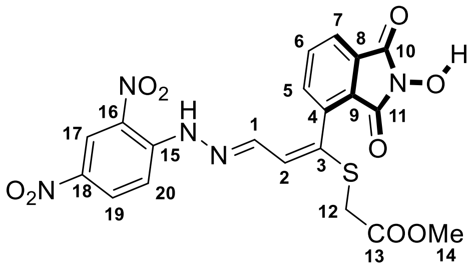

Either of the aldehydes **4** or **5** (100 mg, 0.31 mmol) were dissolved in THF and 2,4-dinitrophenyl hydrazine was added (100 mg, 0.50 mmol). The reaction mixture was heated in an oil bath to 60 ºC for 30 minutes. Evaporation of the solvent under reduced pressure and chromatography over silica gel (20 g) eluting with $CH_2Cl_2$/EtOAc (1/1) yielded compound **6** as a deep red solid (90 mg, 58% yield).

**m.p.:** 123-125 ºC. **IR** (neat, ν in $cm^{-1}$): 3546, 3280, 1787, 1729, 1599, 1515, 1457, 1320. **$^1$H NMR** (400 MHz, $CDCl_3$) δ 10.95 (s, 1H), 9.04 (d, *J* = 2.4 Hz, 1H(17)), 8.30 (dd, *J* = 9.6, 2.4 Hz, 1H(19)), 7.96 – 7.88 (m, 2H (7, 20)), 7.84 (t, *J* = 7.4 Hz, 1H(6)), 7.69 (d, *J* = 7.8 Hz, 1H(5)), 7.39 (d, *J* = 9.6 Hz, 1H(1)), 6.80 (d, *J* = 9.5 Hz, 1H(2)), 3.77 – 3.72 (s, 3H (14)), 3.53 (s, 2H (12)). **$^{13}$C{$^1$H} NMR** (101 MHz, $CDCl_3$, $CD_3OD$) δ 169.2 (C(13)), 163.7 (C(10)), 162.9 (C(11)), 145.2 (CH(1)), 144.3 (C(15)), 141.1 (C(3)), 138.2 (C(18)), 136.0 (CH(5)), 134.5 (CH(6)), 133.2 (C(4)), 130.4 (C(8)), 129.8 (CH(19)), 129.4 (C(16)), 126.8 (C(11)), 124.9 (CH(2)), 124.1 (CH(7)), 123.3 (CH(17)), 116.8 (CH(20)), 52.9 ($CH_3$(14)), 34.6 ($CH_2$(12)). **HRMS** (ESI) m/z calcd. for $C_{20}H_{14}N_5O_9S^-$ ($[M-H]^-$) 500.0518, found 500.0518.

**9-acetoxy-7-chloro-3-oxo-3*H*,9*H*-naphtho[1,8-*cd*][1,2]oxazine 1-oxide (12)**

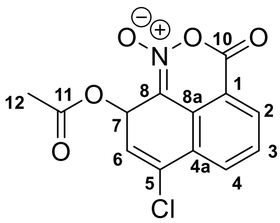

Compound **1** (30 mg, 0.1 mmol) was suspended in oxalyl chloride (1.5 ml, 17.8 mmol) and a trace of triethylammonium bromide was added. The mixture was sonicated for 1 hour at 40-50 ºC until the solution became homogeneous. Bubbles of $CO_2$ and HCl were observed during the reaction. Then, the reaction mixture was concentrated under reduced pressure at 20 ºC. The crude reaction mixture was dissolved in 1 ml of $CDCl_3$ and cooled down at 0 ºC under argon atmosphere. Potassium acetate (30 mg, 0.3 mmol) and acetic acid (30 mL, 0.5 mmol) were added. The reaction mixture was stirred for 40 minutes, added over water and extracted twice with $CH_2Cl_2$. The combined organic phases were dried over $Na_2SO_4$, filtered and concentrated under reduced pressure to yield compound **12** as a pale yellow solid (30 mg, 99% yield).

(Melting point and IR were not measured due to the instability of the compound). **$^{1}$H NMR** (400 MHz, $CDCl_3$) δ 8.25 (d, *J* = 7.9 Hz, 1H(2)), 8.14 (d, *J* = 7.8 Hz, 1H(4)), 7.71 (t, *J* = 7.9 Hz, 1H(3)), 6.72 (d, *J* = 5.2 Hz, 1H(7)), 6.41 (d, *J* = 5.2 Hz, 1H(6)), 2.13 (s, 3H(12)). **$^{13}$C NMR {$^{1}$H}** (101 MHz, $CDCl_3$) δ 169.4 (C(11)), 161.9 (C(10)), 133.0 (C(5)), 132.4 (CH(4)), 130.6 (CH(3)), 130.2 (CH(2)), 127.0 (C(4a)), 125.7 (C(8a)), 121.8 (CH(6)), 117.6 (C(1)), 115.5 (C(8)), 63.9 (CH(7)), 20.1 ($CH_3$(12)). **HRMS** (ESI) m/z calcd. for $C_{13}H_8ClNNaO_5^+$ ($[M+Na]^+$) 315.9983, found 315.9976.

## 3 Crystallographic Data

• *Procedure for the single crystal preparation*

Compound **6**. Single crystals suitable for X-ray diffraction measurements were obtained by slowly evaporating a solution of compound **6** in $CHCl_3$/EtOH in a clean and dry 10 mL glass vial. The mouth of the glass vial was covered with a balloon, fastened with a rubber band, and kept it for slow evaporation at 4 °C. At the end of the process, single crystals were obtained after 5-6 days.

• *X-Ray Crystallography*

Suitable single crystals of compound **6** were mounted on glass fibre for data collection on a Bruker Kappa APEX II diffractometer. Data were collected at 298(2) K using Cu $K_\alpha$ radiation ($\lambda$ = 1.54178 Å) and ω scan technique, and were corrected for Lorentz and polarization effects. The detector was placed at a distance of approximately 37.5 mm from the crystal.
A series of narrow frames of data were collected with a scan width of 0.5° in ω and an exposure time of 10 s per frame. The data were integrated with SAINT[1] to a resolution of 0.78 Å using a narrow-frame algorithm. Data were corrected for absorption effects using the multi-scan method using SADABS.[2]
Subsequent structure solution and refinement were carried out with SHELXT and SHELXL, respectively.[3,4] The structures were solved by direct methods combined with difference Fourier synthesis and refined by full-matrix least-squares procedures, with anisotropic thermal parameters in the last cycles of refinement for all non-hydrogen atoms. The refinement was based on $F^2$ for all reflections, weighted R factors (wR) and goodness-of-fit (GoF) values are based on $F^2$, while conventional R factors (R) are based on F. The $Fo^2 > 2\sigma(Fo^2)$ criterion was used only for calculating R factors and it is not relevant to the choice of reflections for the refinement. The R factors based on $F^2$ are about twice as large as those based on F. Scattering factors were taken from the International Tables for Crystallography.[5] Hydrogen atoms were positioned geometrically. Mercury 4.2.0 program was used for analysis and molecular and crystal structure drawings preparation.[6]

- *Crystal Data*

**Table S1**. Crystal data and structure refinement for compound **6**. CCDC 2351359.

| | |
|---|---|
| Empirical formula | $C_{23}H_{21}Cl_3N_5O_{10}S$ |
| Formula weight | 665.86 |
| Temperature (K) | 298(2) |
| Wavelength (Å) | 1.54178 |
| Crystal system | Monoclinic |
| Space group | $P2_1/c$ |
| Unit cell dimensions: | |
| a (Å) | 17.4129(9) |
| b (Å) | 8.4850(5) |
| c (Å) | 20.4961(8) |
| α [º], β[º], γ[º] | 90.00,100.521(3), 90.00 |
| Volume | 2977.4(3) |
| Z, Density (calculated) ($Mg/m^3$) | 4, 1.485 |
| Absorption coefficient ($mm^{-1}$) | 3.987 |
| F(000) | 1364 |
| Crystal size (mm) | 0.13 x 0.11 x 0.09 |
| 2Θ range for data collection(°) | 2.58 to 66.98 |
| Limiting indices | -20≤ h ≤19, -10≤ k ≤9, -21≤ l ≤23 |
| Reflections collected / Independent | 19491/5021 (Rint = 0.0628) |
| Refinement method | Full-matrix least-squares on $F^2$ |
| Data / restraints / parameters | 5021/ 0 / 382 |
| Goodness-of-fit on $F^2$ | 1.933 |
| Final R indices[I>2σ(I)] | R1 = 0.1010, wR2 = 0.2823 |
| R indices (all data) | R1 = 0.1259, wR2 = 0.2964 |
| Largest diff. peak and hole ($eÅ^{-3}$) | 0.780 and -0.857 |

- *Molecular structure of compound **6***

**Compound 6:** $C_{23}H_{21}Cl_3N_5O_{10}S$

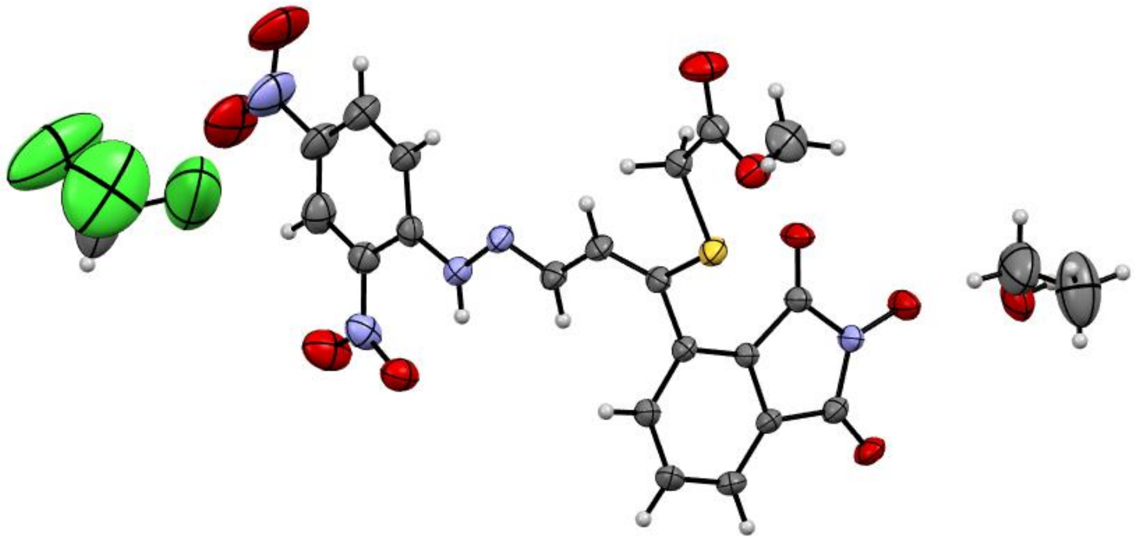

**Figure S1.** Molecular structure of compound **6**. Displacement ellipsoids are drawn at 50 % probability level. Hydrogen atoms are shown as spheres of arbitrary radius.

## 4 Computacional Studies

### DFT calculations on the reaction mechanism

DFT calculations were done using Gaussian16 software [10]. The functional w-B97XD [11] was used in combination with 6-31+G(d,p) basis set [12]. Solvent effects (chloroform) were introduced by means of implicit SMD solvation model [13], and Gibbs correction to the single point energy was evaluated by the rigid-rotor harmonic approximation [14]) as implemented in Goodvibes software [15] for a temperature 298 K and a threshold of 100 $cm^{-1}$.

For each transformation, several TS structures (corresponding to different conformations) were calculated. The energies in the diagram of Figure 10 correspond to the Gibbs free energy of the most stable TSs, but product distribution was calculated using a Maxwell-Boltzmann average considering the Gibbs free energies of each TS structure obtained.

### Cartesian coordinates of TS structures found:

### Dihydrogenphosphate catalysed water addition to carbonyl C of the naphtho oxazinium cation (A) to yield carboxylate E (TS-AE).

**Single point energy: -1860.2542838 Hartrees**
**Gibbs free energy: -1860.060879 Hartrees**
**No. img. Freqs= 1**

| | | | |
|---|---|---|---|
| C | 2.36906 | 1.48794 | -0.95576 |
| C | 1.29619 | 1.88002 | -0.18158 |
| C | 0.60058 | 0.95584 | 0.64720 |
| C | 1.11199 | -0.37076 | 0.72295 |
| C | 2.25069 | -0.75238 | -0.07366 |
| C | 2.83974 | 0.17661 | -0.91681 |
| C | -0.58367 | 1.22857 | 1.39778 |
| C | 0.47711 | -1.30936 | 1.58042 |
| H | 3.68703 | -0.08027 | -1.54073 |
| C | -0.62637 | -0.99181 | 2.33078 |
| C | -1.18773 | 0.28561 | 2.21923 |
| H | 0.86874 | -2.32026 | 1.64278 |
| H | -1.09573 | -1.73153 | 2.96959 |
| H | -2.11167 | 0.52642 | 2.73257 |
| C | -1.33383 | 2.43484 | 1.28818 |
| O | -2.13353 | 3.15580 | 1.66254 |
| N | 0.96685 | 3.28029 | -0.19856 |
| O | 0.18100 | 3.70843 | 0.66114 |
| S | 2.83860 | -2.40054 | 0.07140 |
| C | 4.22244 | -2.47449 | -1.08773 |
| H | 4.59602 | -3.49972 | -1.00931 |
| H | 3.89278 | -2.29654 | -2.11500 |
| H | 5.02189 | -1.78455 | -0.80373 |
| O | 1.48928 | 4.01779 | -1.00311 |
| H | 2.86739 | 2.21931 | -1.58279 |
| O | -1.60527 | 2.33719 | -0.90038 |
| H | -2.06627 | 1.46116 | -1.08577 |
| H | -2.22565 | 3.02664 | -1.15957 |
| O | -2.76702 | 0.07230 | -1.28531 |
| P | -3.68831 | -0.48356 | -0.21014 |
| O | -3.19970 | -1.97670 | 0.21868 |
| O | -5.10997 | -0.87011 | -0.92958 |
| O | -3.89292 | 0.30475 | 1.04455 |
| H | -5.35744 | -0.20310 | -1.57930 |
| H | -2.99558 | -2.52083 | -0.55059 |

**Single point energy: -1860.25762445 Hartrees**
**Gibbs free energy: -1860.061949 Hartrees**
**No. img. Freqs= 1**

| | | | |
|---|---|---|---|
| C | -0.78832 | -0.65038 | 1.30465 |
| C | 0.33968 | 0.05314 | 0.90417 |
| C | 0.24376 | 1.26426 | 0.15759 |
| C | -1.05448 | 1.76441 | -0.12981 |
| C | -2.21335 | 1.02518 | 0.30957 |
| C | -2.05448 | -0.16554 | 1.01378 |
| C | 1.36379 | 1.98690 | -0.30590 |
| C | -1.17085 | 2.97186 | -0.85753 |
| H | -2.91012 | -0.74065 | 1.34495 |

| | | | |
|---|---|---|---|
| C | -0.06096 | 3.66542 | -1.28337 |
| C | 1.21946 | 3.16619 | -1.01149 |
| H | -2.15383 | 3.36623 | -1.09651 |
| H | -0.17410 | 4.58911 | -1.84159 |
| H | 2.10204 | 3.69208 | -1.36343 |
| C | 2.73359 | 1.49275 | -0.10438 |
| O | 3.76957 | 2.03478 | 0.03580 |
| N | 1.58547 | -0.47059 | 1.26619 |
| O | 2.66162 | 0.18551 | 0.98185 |
| S | -3.78539 | 1.67854 | -0.06986 |
| C | -4.94400 | 0.46596 | 0.60266 |
| H | -5.93420 | 0.86508 | 0.36441 |
| H | -4.82752 | -0.50569 | 0.11478 |
| H | -4.84809 | 0.37900 | 1.68860 |
| O | 1.70852 | -1.49157 | 1.88870 |
| H | -0.67559 | -1.57964 | 1.85151 |
| O | 2.89693 | 0.35471 | -1.57936 |
| H | 2.26031 | -0.46263 | -1.57713 |
| H | 3.80003 | 0.01133 | -1.54757 |
| O | 1.39853 | -1.61639 | -1.42360 |
| P | 0.02980 | -1.88831 | -2.02854 |
| O | -0.63306 | -2.96448 | -0.98718 |
| O | 0.23127 | -2.76037 | -3.39415 |
| O | -0.91685 | -0.77729 | -2.33309 |
| H | 0.92551 | -3.42229 | -3.29286 |
| H | -1.55485 | -3.13475 | -1.21378 |

**Single point energy: -1860.25799099 Hartrees**
**Gibbs free energy: -1860.06294 Hartrees**
**No. img. Freqs= 1**

| | | | |
|---|---|---|---|
| C | -0.81389 | -0.45067 | 1.72902 |
| C | 0.31985 | 0.17728 | 1.22854 |
| C | 0.23139 | 1.21034 | 0.24698 |
| C | -1.06174 | 1.65675 | -0.13270 |
| C | -2.22787 | 1.00344 | 0.41848 |
| C | -2.07650 | -0.04609 | 1.31854 |
| C | 1.35515 | 1.81261 | -0.35942 |
| C | -1.16778 | 2.72094 | -1.05985 |
| H | -2.93306 | -0.57226 | 1.72013 |
| C | -0.05277 | 3.31921 | -1.60204 |
| C | 1.22329 | 2.84985 | -1.26286 |
| H | -2.14805 | 3.07913 | -1.35967 |
| H | -0.15887 | 4.13591 | -2.30871 |
| H | 2.11008 | 3.28170 | -1.71741 |
| C | 2.70675 | 1.29061 | -0.13113 |
| O | 3.78499 | 1.74761 | -0.20438 |
| N | 1.55669 | -0.21463 | 1.75370 |
| O | 2.63415 | 0.34058 | 1.31905 |
| S | -3.79433 | 1.57583 | -0.09458 |
| C | -4.96433 | 0.50103 | 0.76716 |
| H | -5.95034 | 0.84645 | 0.44290 |
| H | -4.83658 | -0.54273 | 0.46774 |
| H | -4.88689 | 0.61454 | 1.85211 |
| O | 1.65913 | -1.02089 | 2.64364 |
| H | -0.71017 | -1.25616 | 2.44550 |
| O | 2.66222 | -0.22553 | -1.21598 |
| H | 1.79226 | -0.78396 | -1.30582 |
| H | 3.34619 | -0.83390 | -0.90397 |
| O | 0.60082 | -1.62404 | -1.41984 |
| P | 0.50048 | -2.89274 | -0.58620 |
| O | 1.98007 | -2.97693 | 0.12160 |
| O | 0.46681 | -4.17124 | -1.59315 |
| O | -0.57721 | -3.08222 | 0.42756 |
| H | 0.99792 | -4.01806 | -2.38339 |
| H | 1.98776 | -3.58037 | 0.87372 |

**Single point energy: -1860.25818918 Hartrees**
**Gibbs free energy: -1860.064136 Hartrees**
**No. img. Freqs= 1**

| | | | |
|---|---|---|---|
| C | 1.52971 | -1.36308 | -1.75056 |
| C | 0.78482 | -0.24202 | -1.40891 |
| C | 1.12412 | 0.56932 | -0.28575 |
| C | 2.31241 | 0.25629 | 0.42553 |
| C | 3.07212 | -0.91428 | 0.05232 |
| C | 2.66025 | -1.69973 | -1.01955 |
| C | 0.34048 | 1.65695 | 0.15867 |
| C | 2.69760 | 1.08961 | 1.50357 |
| H | 3.20257 | -2.59231 | -1.30448 |
| C | 1.94117 | 2.17558 | 1.88113 |
| C | 0.73725 | 2.45044 | 1.21708 |
| H | 3.60897 | 0.87410 | 2.05341 |
| H | 2.25877 | 2.80309 | 2.70761 |
| H | 0.10335 | 3.27107 | 1.53994 |
| C | -0.98774 | 1.91146 | -0.41276 |
| O | -1.67758 | 2.85844 | -0.48838 |
| N | -0.30605 | 0.07549 | -2.22584 |
| O | -1.02874 | 1.10565 | -1.94629 |
| S | 4.48497 | -1.30274 | 1.00165 |
| C | 5.09609 | -2.83889 | 0.27126 |
| H | 5.97305 | -3.10288 | 0.86926 |
| H | 4.35410 | -3.63792 | 0.35482 |
| H | 5.40569 | -2.69206 | -0.76692 |
| O | -0.57783 | -0.54788 | -3.21940 |
| H | 1.22240 | -1.97555 | -2.58926 |
| O | -1.95566 | 0.56031 | 0.41061 |
| H | -1.49867 | -0.34016 | 0.67392 |
| H | -2.75388 | 0.36657 | -0.09821 |
| O | -0.81007 | -1.51402 | 1.10739 |
| P | -0.71952 | -2.91676 | 0.53117 |
| O | -1.95640 | -3.72991 | 1.22164 |

| | | | |
|---|---|---|---|
| O | 0.61078 | -3.61363 | 1.17394 |
| O | -0.68555 | -3.14608 | -0.94414 |
| H | 0.73167 | -3.35306 | 2.09478 |
| H | -2.10824 | -4.57247 | 0.77768 |

**Single point energy: -1860.25741268 Hartrees**
**Gibbs free energy: -1860.062626 Hartrees**
**No. img. Freqs= 1**

| | | | |
|---|---|---|---|
| C | 1.48627 | 0.60016 | -1.18678 |
| C | 0.71975 | 1.39425 | -0.34700 |
| C | 0.40212 | 0.99428 | 0.98335 |
| C | 0.94891 | -0.22862 | 1.45634 |
| C | 1.75211 | -1.03499 | 0.56915 |
| C | 1.99890 | -0.60645 | -0.73106 |
| C | -0.41635 | 1.74965 | 1.84975 |
| C | 0.66793 | -0.62805 | 2.78440 |
| H | 2.58691 | -1.20216 | -1.41808 |
| C | -0.11561 | 0.13953 | 3.61594 |
| C | -0.67120 | 1.33615 | 3.14297 |
| H | 1.07318 | -1.56067 | 3.16535 |
| H | -0.31622 | -0.18517 | 4.63180 |
| H | -1.31182 | 1.93677 | 3.78193 |
| C | -1.07601 | 2.98349 | 1.39654 |
| O | -1.41040 | 3.96328 | 1.95877 |
| N | 0.27443 | 2.62262 | -0.85225 |
| O | -0.39848 | 3.42177 | -0.09104 |
| S | 2.37770 | -2.54434 | 1.18709 |
| C | 3.28751 | -3.24767 | -0.20727 |
| H | 3.68510 | -4.19524 | 0.16773 |
| H | 2.62399 | -3.45087 | -1.05238 |
| H | 4.12103 | -2.60522 | -0.50433 |
| O | 0.53931 | 3.00675 | -1.95846 |
| H | 1.68434 | 0.92487 | -2.20161 |
| O | -2.60121 | 2.26006 | 0.58216 |
| H | -2.42592 | 1.72354 | -0.28721 |
| H | -3.14971 | 3.01766 | 0.33745 |
| O | -2.04584 | 1.08261 | -1.52545 |
| P | -2.02836 | -0.38011 | -1.93030 |
| O | -1.40726 | -1.14872 | -0.63419 |
| O | -0.80885 | -0.57108 | -3.00976 |
| O | -3.26363 | -1.04832 | -2.44331 |
| H | -1.16778 | -0.71523 | -3.89247 |
| H | -1.50156 | -2.10525 | -0.70846 |

**Single point energy: -1860.25490148 Hartrees**
**Gibbs free energy: -1860.060726 Hartrees**
**No. img. Freqs= 1**

| | | | |
|---|---|---|---|
| C | 2.22862 | 1.39610 | -1.11536 |
| C | 1.22812 | 1.88891 | -0.28564 |
| C | 0.58972 | 1.06102 | 0.68245 |
| C | 1.07526 | -0.25770 | 0.86404 |
| C | 2.13415 | -0.74229 | 0.01281 |
| C | 2.66552 | 0.08696 | -0.97687 |
| C | -0.51657 | 1.49268 | 1.44566 |
| C | 0.47319 | -1.06368 | 1.86469 |
| H | 3.44712 | -0.26362 | -1.63972 |
| C | -0.57308 | -0.59940 | 2.62846 |
| C | -1.09533 | 0.68517 | 2.40034 |
| H | 0.83314 | -2.07419 | 2.03633 |
| H | -1.02090 | -1.23586 | 3.38474 |
| H | -1.96276 | 1.03491 | 2.95062 |
| C | -1.15912 | 2.77296 | 1.12432 |
| O | -1.93944 | 3.45524 | 1.67856 |
| N | 0.91806 | 3.25403 | -0.36845 |
| O | 0.05758 | 3.75562 | 0.44842 |
| S | 2.69715 | -2.37102 | 0.27034 |
| C | 3.95952 | -2.61552 | -0.99948 |
| H | 4.30614 | -3.64245 | -0.85182 |
| H | 3.53404 | -2.52624 | -2.00264 |
| H | 4.80224 | -1.93295 | -0.86115 |
| O | 1.47256 | 4.00014 | -1.13384 |
| H | 2.68367 | 2.04443 | -1.85671 |
| O | -1.75163 | 2.40213 | -0.62763 |
| H | -1.93140 | 1.38363 | -0.79641 |
| H | -2.58592 | 2.87721 | -0.73140 |
| O | -2.03003 | -0.01466 | -1.01177 |
| P | -3.11897 | -0.96625 | -0.54058 |
| O | -3.71573 | -0.25321 | 0.80994 |
| O | -2.37364 | -2.30437 | 0.02552 |
| O | -4.22561 | -1.40474 | -1.43707 |
| H | -1.53462 | -2.08350 | 0.44615 |
| H | -4.58799 | -0.60207 | 1.02724 |

**Single point energy: -1860.25820262 Hartrees**
**Gibbs free energy: -1860.062637 Hartrees**
**No. img. Freqs= 1**

| | | | |
|---|---|---|---|
| C | 0.70339 | -0.61282 | -1.80254 |
| C | -0.28080 | 0.23822 | -1.32066 |
| C | -0.00966 | 1.20038 | -0.30328 |
| C | 1.32995 | 1.32123 | 0.15461 |
| C | 2.34372 | 0.43990 | -0.37761 |
| C | 2.00648 | -0.51124 | -1.33339 |
| C | -0.99640 | 2.03454 | 0.26497 |
| C | 1.62464 | 2.29398 | 1.13878 |
| H | 2.74241 | -1.20104 | -1.72681 |
| C | 0.64777 | 3.11654 | 1.65361 |
| C | -0.67822 | 2.97983 | 1.22114 |
| H | 2.64135 | 2.40110 | 1.50469 |
| H | 0.89825 | 3.85940 | 2.40405 |
| H | -1.46238 | 3.60299 | 1.64114 |

| | | | |
|---|---|---|---|
| C | -2.41742 | 1.89110 | -0.09083 |
| O | -3.31253 | 2.65743 | -0.11805 |
| N | -1.56107 | 0.12385 | -1.87814 |
| O | -2.50242 | 0.91650 | -1.48366 |
| S | 3.97135 | 0.61652 | 0.22878 |
| C | 4.89728 | -0.68532 | -0.61609 |
| H | 5.91815 | -0.59650 | -0.23342 |
| H | 4.50637 | -1.67452 | -0.36272 |
| H | 4.90827 | -0.52851 | -1.69823 |
| O | -1.82034 | -0.64812 | -2.76044 |
| H | 0.45318 | -1.35781 | -2.54859 |
| O | -2.90461 | 0.49534 | 1.02306 |
| H | -2.50862 | -0.44116 | 0.78857 |
| H | -3.86538 | 0.42384 | 0.93705 |
| O | -1.97469 | -1.68450 | 0.35806 |
| P | -0.78732 | -2.54673 | 0.75295 |
| O | -1.38479 | -3.59233 | 1.85411 |
| O | 0.24923 | -1.62685 | 1.62516 |
| O | 0.01486 | -3.24209 | -0.29420 |
| H | -0.19796 | -1.12993 | 2.32047 |
| H | -0.78025 | -4.32917 | 2.00133 |

**Single point energy: -1860.25405683 Hartrees**
**Gibbs free energy: -1860.059398 Hartrees**
**No. img. Freqs= 1**

| | | | |
|---|---|---|---|
| C | -0.70500 | 0.54868 | -1.77491 |
| C | 0.30578 | -0.26520 | -1.28947 |
| C | 0.06502 | -1.23755 | -0.27436 |
| C | -1.27186 | -1.40324 | 0.17914 |
| C | -2.31344 | -0.55510 | -0.35357 |
| C | -2.00670 | 0.40260 | -1.30985 |
| C | 1.07621 | -2.04179 | 0.29858 |
| C | -1.54020 | -2.38682 | 1.16031 |
| H | -2.76441 | 1.06506 | -1.70802 |
| C | -0.54084 | -3.17980 | 1.67730 |
| C | 0.78181 | -3.00184 | 1.24863 |
| H | -2.55534 | -2.52787 | 1.51895 |
| H | -0.77116 | -3.93498 | 2.42198 |
| H | 1.58071 | -3.61005 | 1.66334 |
| C | 2.49729 | -1.86844 | -0.05272 |
| O | 3.39508 | -2.62660 | -0.09633 |
| N | 1.58639 | -0.10461 | -1.84785 |
| O | 2.54474 | -0.87291 | -1.46884 |
| S | -3.93606 | -0.77886 | 0.25649 |
| C | -4.90061 | 0.49442 | -0.58953 |
| H | -5.91726 | 0.37765 | -0.20321 |
| H | -4.53751 | 1.49524 | -0.34015 |
| H | -4.91115 | 0.33510 | -1.67127 |
| O | 1.81479 | 0.69252 | -2.71699 |
| H | -0.47932 | 1.30041 | -2.52196 |
| O | 3.03349 | -0.42189 | 0.92003 |
| H | 2.51155 | 0.47887 | 0.80598 |
| H | 3.05399 | -0.66023 | 1.85593 |
| O | 1.86420 | 1.68824 | 0.49911 |
| P | 0.61766 | 2.48367 | 0.84244 |
| O | 1.10412 | 3.53265 | 1.99385 |
| O | -0.42181 | 1.50228 | 1.64305 |
| O | -0.15995 | 3.16055 | -0.23413 |
| H | 0.00145 | 1.04050 | 2.37578 |
| H | 0.46202 | 4.24229 | 2.11302 |

**Single point energy: -1860.25802052 Hartrees**
**Gibbs free energy: -1860.063447 Hartrees**
**No. img. Freqs= 1**

| | | | |
|---|---|---|---|
| C | -0.57171 | 0.24402 | 2.42975 |
| C | 0.50617 | 0.76581 | 1.71753 |
| C | 0.36085 | 1.20884 | 0.36944 |
| C | -0.93289 | 1.20013 | -0.20748 |
| C | -2.04054 | 0.68798 | 0.56210 |
| C | -1.82813 | 0.20112 | 1.85392 |
| C | 1.45698 | 1.62993 | -0.40881 |
| C | -1.07850 | 1.66682 | -1.53624 |
| H | -2.64330 | -0.21071 | 2.43521 |
| C | 0.00134 | 2.12225 | -2.25901 |
| C | 1.28774 | 2.08551 | -1.69956 |
| H | -2.05596 | 1.65756 | -2.00907 |
| H | -0.13500 | 2.47809 | -3.27532 |
| H | 2.15356 | 2.38715 | -2.28186 |
| C | 2.82355 | 1.44822 | 0.11461 |
| O | 3.86400 | 1.90225 | -0.22298 |
| N | 1.71571 | 0.93236 | 2.38657 |
| O | 2.70906 | 1.50564 | 1.77723 |
| S | -3.61588 | 0.71194 | -0.17334 |
| C | -4.68702 | -0.09767 | 1.03629 |
| H | -5.67457 | -0.11537 | 0.56637 |
| H | -4.36198 | -1.12529 | 1.22070 |
| H | -4.74688 | 0.47435 | 1.96628 |
| O | 1.87753 | 0.62993 | 3.54291 |
| H | -0.42718 | -0.10681 | 3.44599 |
| O | 2.90739 | -0.37360 | 0.18419 |
| H | 2.30418 | -0.86412 | -0.54076 |
| H | 3.83307 | -0.60767 | 0.03788 |
| O | 1.48851 | -1.44904 | -1.48917 |
| P | 0.19010 | -2.20896 | -1.25240 |
| O | 0.54772 | -3.74712 | -1.66861 |
| O | -0.86374 | -1.73752 | -2.40653 |
| O | -0.51325 | -2.14794 | 0.06112 |
| H | -0.42027 | -1.58406 | -3.24901 |
| H | -0.16065 | -4.35026 | -1.41501 |

**Single point energy: -1860.25588909 Hartrees**
**Gibbs free energy: -1860.061069 Hartrees**
**No. img. Freqs= 1**

| | | | |
|---|---|---|---|
| C | -0.73208 | 0.51245 | -1.86400 |
| C | 0.28484 | -0.26666 | -1.33590 |
| C | 0.04788 | -1.19591 | -0.28149 |
| C | -1.28712 | -1.34397 | 0.18265 |
| C | -2.33210 | -0.52681 | -0.38513 |
| C | -2.03179 | 0.38455 | -1.39116 |
| C | 1.05923 | -1.98131 | 0.31697 |
| C | -1.55240 | -2.28627 | 1.20472 |
| H | -2.79704 | 1.01477 | -1.82724 |
| C | -0.55332 | -3.06069 | 1.74707 |
| C | 0.76705 | -2.90389 | 1.30367 |
| H | -2.56502 | -2.40796 | 1.57694 |
| H | -0.78055 | -3.78205 | 2.52528 |
| H | 1.56671 | -3.49573 | 1.73992 |
| C | 2.47722 | -1.82540 | -0.04163 |
| O | 3.38149 | -2.57320 | -0.06494 |
| N | 1.56619 | -0.11797 | -1.89750 |
| O | 2.53237 | -0.85189 | -1.47821 |
| S | -3.95221 | -0.73321 | 0.23496 |
| C | -4.93417 | 0.46456 | -0.69561 |
| H | -5.95350 | 0.34521 | -0.31706 |
| H | -4.60103 | 1.48747 | -0.49933 |
| H | -4.92521 | 0.24358 | -1.76671 |
| O | 1.78739 | 0.64053 | -2.80294 |
| H | -0.51030 | 1.22320 | -2.65191 |
| O | 3.01219 | -0.33604 | 0.92502 |
| H | 2.52753 | 0.55942 | 0.70378 |
| H | 2.86921 | -0.51273 | 1.86464 |
| O | 1.88129 | 1.77091 | 0.29278 |
| P | 0.65643 | 2.41733 | 0.92261 |
| O | -0.00777 | 3.25798 | -0.31289 |
| O | 1.15809 | 3.58319 | 1.94883 |
| O | -0.36361 | 1.59312 | 1.63366 |
| H | 1.91220 | 4.06861 | 1.59318 |
| H | -0.85546 | 3.63792 | -0.05357 |

**Single point energy: -1860.25674084 Hartrees**
**Gibbs free energy: -1860.061387 Hartrees**
**No. img. Freqs= 1**

| | | | |
|---|---|---|---|
| C | -0.76623 | 0.48286 | -1.86478 |
| C | 0.27011 | -0.27670 | -1.33387 |
| C | 0.04951 | -1.21255 | -0.27958 |
| C | -1.27970 | -1.37483 | 0.19472 |
| C | -2.33868 | -0.57284 | -0.37082 |
| C | -2.05867 | 0.33369 | -1.39017 |
| C | 1.07438 | -1.98365 | 0.30819 |
| C | -1.52311 | -2.31268 | 1.22475 |
| H | -2.83524 | 0.95156 | -1.82304 |
| C | -0.50740 | -3.06826 | 1.76587 |
| C | 0.80553 | -2.89830 | 1.30922 |
| H | -2.53012 | -2.44433 | 1.60861 |
| H | -0.71791 | -3.78324 | 2.55453 |
| H | 1.61891 | -3.47110 | 1.74488 |
| C | 2.48249 | -1.81577 | -0.08006 |
| O | 3.39604 | -2.55804 | -0.12432 |
| N | 1.54279 | -0.09598 | -1.87901 |
| O | 2.52341 | -0.83314 | -1.47419 |
| S | -3.94781 | -0.78988 | 0.26210 |
| C | -4.94061 | 0.43362 | -0.62251 |
| H | -5.94804 | 0.32680 | -0.20991 |
| H | -4.58061 | 1.44702 | -0.42538 |
| H | -4.97273 | 0.22374 | -1.69521 |
| O | 1.77094 | 0.69264 | -2.76049 |
| H | -0.55971 | 1.19381 | -2.65625 |
| O | 2.97004 | -0.40082 | 1.02687 |
| H | 2.51166 | 0.50757 | 0.82576 |
| H | 3.91782 | -0.27619 | 0.88244 |
| O | 1.92111 | 1.75594 | 0.40668 |
| P | 0.61486 | 2.45231 | 0.78480 |
| O | 0.12042 | 3.23967 | -0.56174 |
| O | 0.98817 | 3.65350 | 1.82510 |
| O | -0.54094 | 1.68918 | 1.32401 |
| H | 1.90250 | 3.94131 | 1.72536 |
| H | 0.86755 | 3.55700 | -1.08217 |

**Single point energy: -1860.25615814 Hartrees**
**Gibbs free energy: -1860.062497 Hartrees**
**No. img. Freqs= 1**

| | | | |
|---|---|---|---|
| C | 1.66776 | 1.04708 | -1.24439 |
| C | 0.87694 | 1.72000 | -0.32037 |
| C | 0.38926 | 1.07570 | 0.85527 |
| C | 0.80248 | -0.26096 | 1.09941 |
| C | 1.63121 | -0.93557 | 0.13052 |
| C | 2.03711 | -0.26903 | -1.02457 |
| C | -0.48976 | 1.68532 | 1.77866 |
| C | 0.35601 | -0.90633 | 2.27847 |
| H | 2.64670 | -0.75949 | -1.77281 |
| C | -0.46628 | -0.27288 | 3.18008 |
| C | -0.91078 | 1.03055 | 2.92013 |
| H | 0.65406 | -1.93089 | 2.47864 |
| H | -0.80002 | -0.78959 | 4.07377 |
| H | -1.60279 | 1.52219 | 3.59734 |
| C | -1.07875 | 2.99816 | 1.50802 |
| O | -1.54184 | 3.85437 | 2.15744 |
| N | 0.60463 | 3.06981 | -0.56890 |
| O | -0.06698 | 3.74596 | 0.29189 |
| S | 2.08607 | -2.58556 | 0.46084 |

| | | | |
|---|---|---|---|
| C | 3.01681 | -3.09205 | -1.00289 |
| H | 3.28577 | -4.13551 | -0.81518 |
| H | 2.39602 | -3.04089 | -1.90172 |
| H | 3.93174 | -2.50513 | -1.11990 |
| O | 1.01679 | 3.64733 | -1.54609 |
| H | 2.00475 | 1.56161 | -2.13772 |
| O | -2.35997 | 2.51095 | 0.21065 |
| H | -2.18714 | 1.71354 | -0.43049 |
| H | -2.62868 | 3.27833 | -0.30990 |
| O | -1.89592 | 0.60943 | -1.32548 |
| P | -2.27522 | -0.84387 | -1.08415 |
| O | -0.97857 | -1.68752 | -1.62228 |
| O | -3.44625 | -1.24310 | -2.15196 |
| O | -2.70294 | -1.32342 | 0.26155 |
| H | -3.31564 | -0.80355 | -3.00002 |
| H | -1.03605 | -2.60798 | -1.34087 |

**Single point energy: -1860.25435591 Hartrees**
**Gibbs free energy: -1860.05864 Hartrees**
**No. img. Freqs= 1**

| | | | |
|---|---|---|---|
| C | 2.54798 | 1.46621 | -1.03722 |
| C | 1.35776 | 1.68140 | -0.33124 |
| C | 0.79013 | 0.66627 | 0.49565 |
| C | 1.50396 | -0.54201 | 0.67954 |
| C | 2.74927 | -0.72840 | -0.02546 |
| C | 3.22421 | 0.27287 | -0.89078 |
| C | -0.45791 | 0.84377 | 1.11224 |
| C | 0.94378 | -1.52260 | 1.53451 |
| H | 4.14351 | 0.13475 | -1.44602 |
| C | -0.26509 | -1.31337 | 2.16093 |
| C | -0.98429 | -0.12821 | 1.93828 |
| H | 1.46288 | -2.46199 | 1.70039 |
| H | -0.68056 | -2.07822 | 2.80887 |
| H | -1.95906 | 0.01566 | 2.39460 |
| C | -1.24502 | 2.05300 | 0.76845 |
| O | -2.21335 | 2.48953 | 1.33915 |
| N | 0.77931 | 2.92949 | -0.38669 |
| O | -0.29937 | 3.19285 | 0.34474 |
| S | 3.60900 | -2.20978 | 0.23363 |
| C | 5.08691 | -2.07236 | -0.79864 |
| H | 5.62666 | -3.00929 | -0.63355 |
| H | 4.82608 | -1.99530 | -1.85748 |
| H | 5.71766 | -1.23832 | -0.48015 |
| O | 1.22378 | 3.85971 | -1.00670 |
| H | 2.94623 | 2.24407 | -1.68055 |
| O | -1.66036 | 1.62913 | -0.95194 |
| H | -2.35712 | 0.79218 | -0.83086 |
| H | -2.17716 | 2.37272 | -1.29419 |
| O | -3.16199 | -0.22286 | -0.56172 |
| P | -4.54268 | -0.11876 | 0.08698 |
| O | -5.31283 | 0.99324 | -0.83262 |
| O | -4.38250 | 0.62602 | 1.52558 |
| O | -5.35677 | -1.34796 | 0.28572 |
| H | -3.76025 | 1.37240 | 1.49364 |
| H | -6.24751 | 1.05645 | -0.60347 |

**Single point energy: -1860.25105137 Hartrees**
**Gibbs free energy: -1860.056276 Hartrees**
**No. img. Freqs= 1**

| | | | |
|---|---|---|---|
| C | 1.65078 | -1.46192 | -1.47605 |
| C | 0.86699 | -0.35704 | -1.19186 |
| C | 1.24940 | 0.59415 | -0.20032 |
| C | 2.51627 | 0.41939 | 0.42372 |
| C | 3.31972 | -0.73484 | 0.10646 |
| C | 2.85951 | -1.66356 | -0.81914 |
| C | 0.46081 | 1.69842 | 0.22676 |
| C | 2.95825 | 1.38428 | 1.36733 |
| H | 3.43594 | -2.54573 | -1.06915 |
| C | 2.19270 | 2.46851 | 1.71345 |
| C | 0.91406 | 2.61531 | 1.15904 |
| H | 3.92722 | 1.26421 | 1.84254 |
| H | 2.54629 | 3.18510 | 2.44566 |
| H | 0.26827 | 3.42605 | 1.48175 |
| C | -0.91639 | 1.87339 | -0.16886 |
| O | -1.76839 | 2.64119 | -0.24498 |
| N | -0.29877 | -0.17188 | -1.99718 |
| O | -0.99441 | 0.85433 | -1.82066 |
| S | 4.85642 | -0.91203 | 0.92744 |
| C | 5.53197 | -2.45396 | 0.27001 |
| H | 6.49135 | -2.57684 | 0.78112 |
| H | 4.89098 | -3.30622 | 0.51187 |
| H | 5.71051 | -2.38526 | -0.80659 |
| O | -0.58861 | -0.96840 | -2.85934 |
| H | 1.32500 | -2.16727 | -2.23301 |
| O | -1.78995 | 0.26044 | 0.71383 |
| H | -1.81330 | 0.61191 | 1.66980 |
| H | -1.26657 | -0.54794 | 0.78866 |
| O | -1.65418 | 1.17392 | 3.07442 |
| P | -0.45978 | 0.79531 | 3.93017 |
| O | 0.31338 | -0.34223 | 3.02157 |
| O | -0.99669 | -0.05232 | 5.21826 |
| O | 0.51228 | 1.80927 | 4.43150 |
| H | -1.79386 | -0.55041 | 5.00446 |
| H | 1.19337 | -0.52625 | 3.36913 |

**Single point energy: -1860.25796647 Hartrees**
**Gibbs free energy: -1860.06429 Hartrees**
**No. img. Freqs= 1**

| | | | |
|---|---|---|---|
| C | -0.51200 | -0.25899 | 2.06257 |
| C | 0.54591 | 0.42460 | 1.47017 |

| | | | |
|---|---|---|---|
| C | 0.34989 | 1.23952 | 0.31620 |
| C | -0.97236 | 1.44600 | -0.14712 |
| C | -2.05993 | 0.75979 | 0.50912 |
| C | -1.80061 | -0.09366 | 1.58134 |
| C | 1.42366 | 1.81174 | -0.39338 |
| C | -1.16645 | 2.28629 | -1.27018 |
| H | -2.59821 | -0.64635 | 2.06101 |
| C | -0.10341 | 2.87846 | -1.91498 |
| C | 1.20943 | 2.62111 | -1.48919 |
| H | -2.17013 | 2.46728 | -1.64368 |
| H | -0.27651 | 3.52075 | -2.77261 |
| H | 2.05758 | 3.03613 | -2.02593 |
| C | 2.80447 | 1.42945 | -0.03980 |
| O | 3.84748 | 1.91905 | -0.31526 |
| N | 1.79932 | 0.35123 | 2.07504 |
| O | 2.78674 | 1.03259 | 1.57784 |
| S | -3.66819 | 1.02965 | -0.10049 |
| C | -4.70294 | -0.10148 | 0.85689 |
| H | -5.70888 | 0.02440 | 0.44615 |
| H | -4.38479 | -1.13743 | 0.71171 |
| H | -4.71621 | 0.16592 | 1.91692 |
| O | 2.00625 | -0.26354 | 3.09007 |
| H | -0.32776 | -0.90729 | 2.91168 |
| O | 2.81635 | -0.33399 | -0.48050 |
| H | 1.97659 | -0.74781 | -0.98910 |
| H | 3.61679 | -0.47371 | -1.00204 |
| O | 0.84900 | -1.24432 | -1.60440 |
| P | -0.06894 | -2.34235 | -1.08146 |
| O | 0.15656 | -3.58021 | -2.12034 |
| O | -1.60278 | -1.86673 | -1.37694 |
| O | 0.00070 | -2.79870 | 0.33653 |
| H | -1.67722 | -1.40513 | -2.22046 |
| H | -0.22032 | -4.39850 | -1.77630 |

**Single point energy: -1860.25503612 Hartrees**
**Gibbs free energy: -1860.060475 Hartrees**
**No. img. Freqs= 1**

| | | | |
|---|---|---|---|
| C | -0.75590 | 0.43644 | -1.88982 |
| C | 0.25968 | -0.33802 | -1.35525 |
| C | 0.02286 | -1.26250 | -0.29763 |
| C | -1.31420 | -1.41747 | 0.15862 |
| C | -2.35929 | -0.60614 | -0.41773 |
| C | -2.05801 | 0.30475 | -1.42225 |
| C | 1.03647 | -2.03784 | 0.30935 |
| C | -1.57877 | -2.35682 | 1.18379 |
| H | -2.82243 | 0.93410 | -1.86069 |
| C | -0.57624 | -3.11889 | 1.73791 |
| C | 0.74581 | -2.95515 | 1.30122 |
| H | -2.59366 | -2.48815 | 1.54681 |
| H | -0.80284 | -3.83907 | 2.51746 |
| H | 1.54703 | -3.54011 | 1.74390 |
| C | 2.45384 | -1.88184 | -0.05431 |
| O | 3.35691 | -2.63188 | -0.07077 |
| N | 1.54231 | -0.19361 | -1.91669 |
| O | 2.51063 | -0.92006 | -1.48691 |
| S | -3.98276 | -0.81249 | 0.19956 |
| C | -4.96947 | 0.35563 | -0.76404 |
| H | -5.99127 | 0.23061 | -0.39406 |
| H | -4.65186 | 1.38681 | -0.58639 |
| H | -4.94552 | 0.11290 | -1.83008 |
| O | 1.76254 | 0.55003 | -2.83342 |
| H | -0.53304 | 1.14717 | -2.67707 |
| O | 2.99525 | -0.38480 | 0.90685 |
| H | 2.50964 | 0.50872 | 0.68514 |
| H | 2.87617 | -0.55488 | 1.85057 |
| O | 1.84898 | 1.69519 | 0.23322 |
| P | 0.73274 | 2.52534 | 0.83963 |
| O | -0.31661 | 1.42025 | 1.42141 |
| O | -0.13984 | 3.15210 | -0.39719 |
| O | 1.03208 | 3.59452 | 1.84086 |
| H | 0.02994 | 4.09756 | -0.47660 |
| H | -1.00704 | 1.83054 | 1.95489 |

**Single point energy: -1860.25500265 Hartrees**
**Gibbs free energy: -1860.060205 Hartrees**
**No. img. Freqs= 1**

| | | | |
|---|---|---|---|
| C | -0.75967 | 0.48771 | -1.85398 |
| C | 0.26834 | -0.27467 | -1.31878 |
| C | 0.03631 | -1.21543 | -0.27234 |
| C | -1.29924 | -1.38014 | 0.18491 |
| C | -2.35053 | -0.56675 | -0.37918 |
| C | -2.05847 | 0.34504 | -1.38740 |
| C | 1.05145 | -1.99868 | 0.32202 |
| C | -1.56029 | -2.33595 | 1.19521 |
| H | -2.82715 | 0.97090 | -1.82278 |
| C | -0.55655 | -3.10717 | 1.73442 |
| C | 0.76379 | -2.93374 | 1.29871 |
| H | -2.57345 | -2.47277 | 1.56036 |
| H | -0.78122 | -3.83994 | 2.50260 |
| H | 1.56695 | -3.52390 | 1.73103 |
| C | 2.46973 | -1.83041 | -0.02892 |
| O | 3.37700 | -2.57343 | -0.07130 |
| N | 1.55030 | -0.09781 | -1.86433 |
| O | 2.52577 | -0.81764 | -1.44340 |
| S | -3.96593 | -0.77823 | 0.24750 |
| C | -4.95295 | 0.43517 | -0.65733 |
| H | -5.96602 | 0.32335 | -0.26017 |
| H | -4.60376 | 1.45235 | -0.46032 |
| H | -4.96718 | 0.21977 | -1.72937 |
| O | 1.76872 | 0.67889 | -2.75772 |

| | | | |
|---|---|---|---|
| H | -0.54593 | 1.19962 | -2.64256 |
| O | 2.99293 | -0.36840 | 0.97987 |
| H | 2.53537 | 0.54111 | 0.75587 |
| H | 2.81524 | -0.55584 | 1.91141 |
| O | 1.94588 | 1.77511 | 0.34517 |
| P | 0.66124 | 2.45361 | 0.81899 |
| O | 0.04872 | 3.22389 | -0.48581 |
| O | 1.09757 | 3.66535 | 1.82097 |
| O | -0.43031 | 1.67118 | 1.45747 |
| H | 1.99152 | 3.97632 | 1.63832 |
| H | 0.74666 | 3.56478 | -1.05738 |

**Structure of the carboxylate E**

**Single point energy: -1860.31466331 Hartrees**
**Gibbs free energy: -1860.117396 Hartrees**
**No. img. Freqs= 0**

| | | | |
|---|---|---|---|
| C | 0.46450 | -0.24175 | 2.12010 |
| C | -0.65307 | -0.50947 | 1.37121 |
| C | -0.57097 | -1.06420 | 0.05613 |
| C | 0.72531 | -1.47275 | -0.38467 |
| C | 1.88353 | -1.21308 | 0.43269 |
| C | 1.74017 | -0.57428 | 1.64565 |
| C | -1.66523 | -1.22025 | -0.85520 |
| C | 0.85321 | -2.12339 | -1.64090 |
| H | 2.59287 | -0.34284 | 2.27210 |
| C | -0.23311 | -2.35826 | -2.44332 |
| C | -1.49648 | -1.88077 | -2.05331 |
| H | 1.83247 | -2.44934 | -1.97830 |
| H | -0.11660 | -2.87189 | -3.39264 |
| H | -2.34824 | -1.98932 | -2.71867 |
| C | -2.99593 | -0.56684 | -0.69247 |
| O | -4.05717 | -1.03879 | -1.00577 |
| N | -1.92609 | -0.33703 | 2.06690 |
| O | -2.81040 | -1.15314 | 1.84757 |
| S | 3.45779 | -1.71887 | -0.18835 |
| C | 4.61100 | -1.18229 | 1.09543 |
| H | 5.59877 | -1.47854 | 0.73020 |
| H | 4.59175 | -0.09598 | 1.22192 |
| H | 4.41869 | -1.69059 | 2.04476 |
| O | -2.03087 | 0.57216 | 2.86874 |
| H | 0.35420 | 0.19415 | 3.10750 |
| O | -2.87939 | 0.71349 | -0.25563 |
| H | -1.50051 | 1.73934 | -0.84455 |
| H | -3.76598 | 1.09825 | -0.16467 |
| O | -0.84902 | 2.21296 | -1.39599 |
| P | 0.37716 | 2.83952 | -0.60377 |
| O | 0.68201 | 4.06058 | -1.59744 |
| O | 1.60173 | 1.81846 | -0.78115 |
| O | 0.18308 | 3.17495 | 0.81888 |
| H | 1.71738 | 1.49071 | -1.68310 |
| H | 1.36436 | 4.65553 | -1.25922 |

**TS structures for the phosphate catalysed water addition to C-7 of the naphtho oxazinium cation A to yield intermediate B (TS-AB).**

**Single point energy: -1860.2599397 Hartrees**
**Gibbs free energy: -1860.064382 Hartrees**
**No. img. Freqs= 1**

| | | | |
|---|---|---|---|
| C | -1.18830 | -1.51741 | 1.64510 |
| C | -0.19198 | -0.52264 | 1.35533 |
| C | -0.32065 | 0.35741 | 0.23310 |
| C | -1.50312 | 0.32560 | -0.53389 |
| C | -2.59814 | -0.55001 | -0.09748 |
| C | -2.43820 | -1.39815 | 0.96897 |
| C | 0.71317 | 1.23547 | -0.10370 |
| C | -1.57674 | 1.15272 | -1.66224 |
| H | -3.24584 | -2.03017 | 1.31701 |
| C | -0.52836 | 2.00002 | -2.01003 |
| C | 0.61762 | 2.05553 | -1.22537 |
| H | -2.46157 | 1.13963 | -2.29228 |
| H | -0.61251 | 2.62365 | -2.89437 |
| H | 1.43921 | 2.71958 | -1.47564 |
| C | 1.89973 | 1.28399 | 0.74207 |
| O | 2.84888 | 2.00252 | 0.65814 |
| N | 0.91188 | -0.51104 | 2.10846 |
| O | 1.91355 | 0.41361 | 1.85231 |
| S | -4.10128 | -0.39860 | -0.97255 |
| C | -5.17237 | -1.64629 | -0.22384 |
| H | -6.10690 | -1.59191 | -0.78970 |
| H | -4.74806 | -2.64857 | -0.33196 |
| H | -5.37939 | -1.41706 | 0.82522 |
| O | 1.16198 | -1.22614 | 3.04617 |
| H | -1.17718 | -1.96485 | 2.63197 |
| O | -0.41067 | -3.14752 | 0.93546 |
| H | 0.57570 | -2.95381 | 0.66907 |
| H | -0.87002 | -3.29403 | 0.09779 |
| O | 1.90664 | -2.54548 | 0.28946 |

| | | | |
|---|---|---|---|
| P | 2.34129 | -2.16715 | -1.11591 |
| O | 2.94936 | -0.64684 | -1.05530 |
| O | 0.93729 | -1.87064 | -1.89433 |
| O | 3.25026 | -3.05613 | -1.89924 |
| H | 1.06795 | -1.76437 | -2.84375 |
| H | 3.91088 | -0.65915 | -0.98295 |

**Single point energy: -1860.26277693 Hartrees**
**Gibbs free energy: -1860.065771 Hartrees**
**No. img. Freqs= 1**

| | | | |
|---|---|---|---|
| C | 1.09986 | -1.14072 | -1.73523 |
| C | 0.07318 | -0.13691 | -1.61144 |
| C | 0.17527 | 0.93079 | -0.66152 |
| C | 1.34041 | 1.04589 | 0.11938 |
| C | 2.44001 | 0.10128 | -0.11914 |
| C | 2.31318 | -0.91681 | -1.03294 |
| C | -0.88469 | 1.82462 | -0.48625 |
| C | 1.38387 | 2.05870 | 1.08536 |
| H | 3.12941 | -1.59768 | -1.23817 |
| C | 0.32084 | 2.94014 | 1.26166 |
| C | -0.81905 | 2.83050 | 0.47655 |
| H | 2.25236 | 2.16289 | 1.72912 |
| H | 0.38121 | 3.70671 | 2.02720 |
| H | -1.66001 | 3.50386 | 0.60908 |
| C | -2.07393 | 1.67348 | -1.31179 |
| O | -3.07408 | 2.32675 | -1.31379 |
| N | -1.03113 | -0.29777 | -2.33470 |
| O | -2.04390 | 0.64304 | -2.27608 |
| S | 3.91012 | 0.39524 | 0.76746 |
| C | 4.98346 | -0.98218 | 0.30556 |
| H | 5.88960 | -0.84693 | 0.90288 |
| H | 4.52490 | -1.94054 | 0.56473 |
| H | 5.24811 | -0.94352 | -0.75497 |
| O | -1.28098 | -1.21750 | -3.08687 |
| H | 1.13594 | -1.70665 | -2.65964 |
| O | 0.33090 | -2.68181 | -0.84436 |
| H | 0.01318 | -2.32028 | 0.07455 |
| H | -0.45659 | -2.98635 | -1.31596 |
| O | -0.38004 | -1.72393 | 1.33512 |
| P | -1.72684 | -1.09944 | 1.66055 |
| O | -2.64905 | -2.20771 | 2.42469 |
| O | -2.44288 | -0.97641 | 0.18069 |
| O | -1.82814 | 0.17724 | 2.41798 |
| H | -3.34237 | -0.63126 | 0.24320 |
| H | -2.47161 | -3.10127 | 2.10916 |

**Single point energy: -1860.2604122 Hartrees**
**Gibbs free energy: -1860.064421 Hartrees**
**No. img. Freqs= 1**

| | | | |
|---|---|---|---|
| C | -1.18013 | -1.65812 | 1.18887 |
| C | -0.14052 | -0.68080 | 1.38507 |
| C | -0.24336 | 0.64262 | 0.84663 |
| C | -1.43099 | 1.03005 | 0.19585 |
| C | -2.54357 | 0.07260 | 0.13687 |
| C | -2.41142 | -1.19402 | 0.64358 |
| C | 0.82707 | 1.53463 | 0.94976 |
| C | -1.48477 | 2.31203 | -0.36298 |
| H | -3.23421 | -1.89766 | 0.63109 |
| C | -0.40807 | 3.19036 | -0.27020 |
| C | 0.75229 | 2.80870 | 0.39078 |
| H | -2.37537 | 2.63994 | -0.89124 |
| H | -0.47936 | 4.17624 | -0.71857 |
| H | 1.60162 | 3.48002 | 0.47129 |
| C | 2.03445 | 1.11091 | 1.64680 |
| O | 3.01945 | 1.73920 | 1.89070 |
| N | 0.98538 | -1.09103 | 1.96615 |
| O | 2.03429 | -0.20628 | 2.15398 |
| S | -4.03169 | 0.66678 | -0.55728 |
| C | -5.12107 | -0.77458 | -0.55925 |
| H | -6.04045 | -0.43302 | -1.04336 |
| H | -4.69065 | -1.59127 | -1.14552 |
| H | -5.35520 | -1.09979 | 0.45838 |
| O | 1.22820 | -2.20405 | 2.37548 |
| H | -1.21135 | -2.49487 | 1.87882 |
| O | -0.45434 | -2.77944 | -0.19749 |
| H | 0.39295 | -2.29624 | -0.56008 |
| H | -0.13414 | -3.60356 | 0.19367 |
| O | 1.61320 | -1.59720 | -0.83913 |
| P | 2.07339 | -0.60052 | -1.88857 |
| O | 2.92836 | 0.55842 | -1.10686 |
| O | 0.73553 | 0.24512 | -2.26867 |
| O | 2.81816 | -1.06327 | -3.09878 |
| H | 0.87685 | 0.79949 | -3.04481 |
| H | 3.87550 | 0.39120 | -1.17230 |

**Single point energy: -1860.2596426 Hartrees**
**Gibbs free energy: -1860.063747 Hartrees**
**No. img. Freqs= 1**

| | | | |
|---|---|---|---|
| C | 0.11391 | -1.91681 | -0.94741 |
| C | 0.98172 | -0.87342 | -0.47478 |
| C | 0.73456 | -0.20418 | 0.76701 |
| C | -0.31128 | -0.65726 | 1.59694 |
| C | -1.05598 | -1.85713 | 1.19402 |
| C | -0.80993 | -2.46549 | -0.01115 |
| C | 1.51706 | 0.88808 | 1.15354 |
| C | -0.56405 | 0.04899 | 2.78019 |
| H | -1.33359 | -3.36479 | -0.31042 |
| C | 0.20289 | 1.15160 | 3.14489 |
| C | 1.25607 | 1.56985 | 2.33995 |
| H | -1.37612 | -0.25497 | 3.43428 |

| | | | |
|---|---|---|---|
| H | -0.02123 | 1.68069 | 4.06560 |
| H | 1.87305 | 2.41886 | 2.61811 |
| C | 2.62727 | 1.29621 | 0.29985 |
| O | 3.43019 | 2.15942 | 0.48405 |
| N | 1.97944 | -0.48243 | -1.27425 |
| O | 2.83137 | 0.53500 | -0.86937 |
| S | -2.20751 | -2.47631 | 2.35073 |
| C | -2.96857 | -3.87653 | 1.49942 |
| H | -3.72132 | -4.25640 | 2.19591 |
| H | -3.46480 | -3.55579 | 0.57898 |
| H | -2.23932 | -4.66672 | 1.29963 |
| O | 2.26829 | -0.93486 | -2.35313 |
| H | 0.48277 | -2.55407 | -1.74234 |
| O | -1.01398 | -1.01073 | -2.25108 |
| H | -0.59293 | -0.08187 | -2.45866 |
| H | -1.82157 | -0.80052 | -1.76188 |
| O | 0.01565 | 1.22549 | -2.60242 |
| P | -0.56889 | 2.37674 | -1.80039 |
| O | -0.94503 | 3.61167 | -2.79947 |
| O | 0.71584 | 3.01461 | -1.03570 |
| O | -1.71640 | 2.08872 | -0.87862 |
| H | 0.47996 | 3.77379 | -0.48955 |
| H | -1.89288 | 3.63406 | -2.97257 |

**Single point energy: -1860.25928444 Hartrees**
**Gibbs free energy: -1860.063521 Hartrees**
**No. img. Freqs= 1**

| | | | |
|---|---|---|---|
| C | 0.48226 | -2.00308 | -1.00880 |
| C | 1.39517 | -0.99862 | -0.52488 |
| C | 1.11234 | -0.22312 | 0.64666 |
| C | -0.06185 | -0.48555 | 1.37629 |
| C | -0.94518 | -1.56651 | 0.91965 |
| C | -0.64217 | -2.30328 | -0.19961 |
| C | 1.98464 | 0.79119 | 1.05302 |
| C | -0.32893 | 0.31039 | 2.49682 |
| H | -1.26358 | -3.13174 | -0.51438 |
| C | 0.54302 | 1.31888 | 2.89698 |
| C | 1.70547 | 1.56580 | 2.17828 |
| H | -1.23785 | 0.15696 | 3.07103 |
| H | 0.30349 | 1.92235 | 3.76642 |
| H | 2.39126 | 2.35444 | 2.47182 |
| C | 3.19294 | 1.03469 | 0.27717 |
| O | 4.06481 | 1.82573 | 0.47515 |
| N | 2.46656 | -0.73872 | -1.26764 |
| O | 3.38350 | 0.22006 | -0.86050 |
| S | -2.33871 | -1.89301 | 1.91045 |
| C | -3.27074 | -3.11601 | 0.96206 |
| H | -4.20023 | -3.25991 | 1.52029 |
| H | -3.50690 | -2.73275 | -0.03471 |
| H | -2.73970 | -4.07018 | 0.90422 |
| O | 2.76118 | -1.25259 | -2.32710 |
| H | 0.88583 | -2.78210 | -1.64639 |
| O | -0.30024 | -1.15440 | -2.58818 |
| H | -0.37909 | -0.15743 | -2.32080 |
| H | 0.31185 | -1.19084 | -3.33577 |
| O | -0.31354 | 1.20795 | -1.84097 |
| P | -1.35458 | 2.03728 | -1.11652 |
| O | -2.36994 | 0.92564 | -0.47735 |
| O | -2.27746 | 2.74991 | -2.26454 |
| O | -0.95351 | 3.05567 | -0.09664 |
| H | -2.46036 | 3.66597 | -2.02929 |
| H | -2.98475 | 1.32231 | 0.15007 |

**Single point energy: -1860.26174232 Hartrees**
**Gibbs free energy: -1860.065116 Hartrees**
**No. img. Freqs= 1**

| | | | |
|---|---|---|---|
| C | -1.08471 | -1.53510 | 1.32621 |
| C | -0.04656 | -0.54420 | 1.41127 |
| C | -0.16696 | 0.72176 | 0.75185 |
| C | -1.36770 | 1.04490 | 0.09029 |
| C | -2.48575 | 0.09738 | 0.16825 |
| C | -2.33779 | -1.11498 | 0.79360 |
| C | 0.90529 | 1.61799 | 0.74259 |
| C | -1.43397 | 2.26569 | -0.59487 |
| H | -3.16641 | -1.80618 | 0.88932 |
| C | -0.35745 | 3.14643 | -0.61009 |
| C | 0.81720 | 2.82997 | 0.06394 |
| H | -2.33432 | 2.53913 | -1.13731 |
| H | -0.43737 | 4.08251 | -1.15367 |
| H | 1.66507 | 3.50825 | 0.06435 |
| C | 2.12495 | 1.25853 | 1.45925 |
| O | 3.13452 | 1.88615 | 1.56832 |
| N | 1.06962 | -0.86195 | 2.07382 |
| O | 2.07290 | 0.07691 | 2.22457 |
| S | -3.99969 | 0.63407 | -0.51523 |
| C | -5.08884 | -0.79677 | -0.34376 |
| H | -6.02594 | -0.49812 | -0.82234 |
| H | -4.68209 | -1.66514 | -0.86976 |
| H | -5.28598 | -1.02760 | 0.70691 |
| O | 1.31634 | -1.90740 | 2.62551 |
| H | -1.07737 | -2.32426 | 2.06953 |
| O | -0.33099 | -2.78481 | 0.05003 |
| H | 0.01445 | -2.23459 | -0.76255 |
| H | -1.05017 | -3.34131 | -0.27496 |
| O | 0.49743 | -1.43125 | -1.86251 |
| P | 1.87169 | -0.78935 | -1.80621 |
| O | 1.69568 | 0.61077 | -2.61025 |
| O | 2.88561 | -1.59863 | -2.79902 |
| O | 2.52894 | -0.60409 | -0.46934 |
| H | 3.57734 | -2.03511 | -2.28918 |

| | | | |
|---|---|---|---|
| H | 2.51049 | 1.12668 | -2.60655 |

**Single point energy: -1860.25762506 Hartrees**
**Gibbs free energy: -1860.061851 Hartrees**
**No. img. Freqs= 1**

| | | | |
|---|---|---|---|
| C | 0.53229 | -1.93260 | -0.94412 |
| C | 1.59591 | -1.10391 | -0.43183 |
| C | 1.38741 | -0.23329 | 0.68736 |
| C | 0.15994 | -0.28247 | 1.37578 |
| C | -0.84394 | -1.26797 | 0.95636 |
| C | -0.63193 | -2.06415 | -0.14396 |
| C | 2.38322 | 0.66426 | 1.08594 |
| C | -0.04107 | 0.62024 | 2.42567 |
| H | -1.35529 | -2.81068 | -0.44748 |
| C | 0.94905 | 1.52158 | 2.80907 |
| C | 2.16935 | 1.54466 | 2.14716 |
| H | -0.99114 | 0.63908 | 2.95006 |
| H | 0.76011 | 2.21140 | 3.62533 |
| H | 2.95397 | 2.23688 | 2.43689 |
| C | 3.65662 | 0.66019 | 0.38044 |
| O | 4.62514 | 1.33117 | 0.57956 |
| N | 2.75291 | -1.10534 | -1.09045 |
| O | 3.79054 | -0.27394 | -0.66598 |
| S | -2.25041 | -1.41333 | 1.96731 |
| C | -3.34260 | -2.49887 | 1.02609 |
| H | -4.28340 | -2.51241 | 1.58379 |
| H | -3.51613 | -2.07838 | 0.03144 |
| H | -2.95026 | -3.51803 | 0.96908 |
| O | 3.05272 | -1.76341 | -2.05912 |
| H | 0.81482 | -2.75763 | -1.58876 |
| O | 0.07579 | -0.86992 | -2.50141 |
| H | -0.33605 | -0.00410 | -2.10134 |
| H | -0.63144 | -1.31964 | -2.98144 |
| O | -0.80069 | 1.18136 | -1.43338 |
| P | -2.22382 | 1.59181 | -1.12182 |
| O | -2.20695 | 1.99473 | 0.45987 |
| O | -3.08450 | 0.19142 | -1.16147 |
| O | -2.94326 | 2.63916 | -1.91125 |
| H | -4.00654 | 0.37427 | -1.37435 |
| H | -2.92975 | 2.59852 | 0.66385 |

**Single point energy: -1860.25582053 Hartrees**
**Gibbs free energy: -1860.059425 Hartrees**
**No. img. Freqs= 1**

| | | | |
|---|---|---|---|
| C | -0.39278 | -1.04579 | -1.31556 |
| C | -1.66490 | -0.73144 | -0.71737 |
| C | -1.88868 | 0.53179 | -0.07649 |
| C | -0.89422 | 1.52806 | -0.15980 |
| C | 0.30379 | 1.26309 | -0.97124 |
| C | 0.51231 | 0.02554 | -1.53018 |
| C | -3.07279 | 0.77575 | 0.62860 |
| C | -1.11617 | 2.73436 | 0.51726 |
| H | 1.39532 | -0.18826 | -2.11866 |
| C | -2.28743 | 2.96127 | 1.23548 |
| C | -3.27746 | 1.98774 | 1.28773 |
| H | -0.36514 | 3.51857 | 0.49207 |
| H | -2.42726 | 3.90658 | 1.74997 |
| H | -4.20237 | 2.15550 | 1.83106 |
| C | -4.10126 | -0.25731 | 0.65239 |
| O | -5.17064 | -0.23382 | 1.18343 |
| N | -2.59332 | -1.68757 | -0.68428 |
| O | -3.81931 | -1.43797 | -0.06069 |
| S | 1.37597 | 2.60809 | -1.22362 |
| C | 2.69187 | 1.93249 | -2.26040 |
| H | 3.37774 | 2.76963 | -2.42018 |
| H | 3.22033 | 1.12886 | -1.73915 |
| H | 2.29848 | 1.60498 | -3.22684 |
| O | -2.52682 | -2.80082 | -1.14381 |
| H | -0.34625 | -1.90396 | -1.97630 |
| O | 0.37381 | -2.16899 | 0.10038 |
| H | 1.36531 | -2.21963 | -0.15217 |
| H | 0.40879 | -1.62732 | 0.90398 |
| O | 2.84873 | -2.02883 | -0.37920 |
| P | 3.57030 | -1.26976 | 0.71370 |
| O | 2.34642 | -0.64931 | 1.63140 |
| O | 4.19726 | 0.05543 | -0.01176 |
| O | 4.59840 | -1.89777 | 1.59693 |
| H | 4.96985 | 0.37819 | 0.46596 |
| H | 2.64032 | -0.47544 | 2.53286 |

**Single point energy: -1860.25846782 Hartrees**
**Gibbs free energy: -1860.064047 Hartrees**
**No. img. Freqs= 1**

| | | | |
|---|---|---|---|
| C | -0.22966 | -0.87351 | -1.64015 |
| C | -1.50807 | -0.89032 | -0.97244 |
| C | -1.86560 | 0.12043 | -0.02207 |
| C | -1.00584 | 1.22078 | 0.16257 |
| C | 0.19129 | 1.32984 | -0.68614 |
| C | 0.54294 | 0.31136 | -1.54098 |
| C | -3.04410 | 0.01061 | 0.72320 |
| C | -1.34901 | 2.16215 | 1.14073 |
| H | 1.42331 | 0.38134 | -2.16708 |
| C | -2.51556 | 2.03848 | 1.89233 |
| C | -3.37535 | 0.96782 | 1.68232 |
| H | -0.69838 | 3.01039 | 1.33314 |
| H | -2.75083 | 2.78569 | 2.64341 |
| H | -4.29415 | 0.86392 | 2.25134 |
| C | -3.93099 | -1.11914 | 0.47364 |
| O | -4.98171 | -1.36901 | 0.98411 |
| N | -2.28945 | -1.94648 | -1.16870 |

| | | | |
|---|---|---|---|
| O | -3.51799 | -2.03457 | -0.51274 |
| S | 1.04697 | 2.83897 | -0.60123 |
| C | 2.46798 | 2.61957 | -1.69498 |
| H | 3.00232 | 3.57309 | -1.64639 |
| H | 3.12049 | 1.82790 | -1.31117 |
| H | 2.14757 | 2.44386 | -2.72575 |
| O | -2.06858 | -2.90486 | -1.87625 |
| H | -0.12407 | -1.47966 | -2.53334 |
| O | 0.63627 | -2.23820 | -0.54808 |
| H | 1.58383 | -1.89390 | -0.29923 |
| H | 0.76012 | -3.05245 | -1.05267 |
| O | 2.92218 | -1.45453 | -0.01920 |
| P | 3.44740 | -0.38745 | 0.91548 |
| O | 4.39420 | -1.17609 | 1.98365 |
| O | 2.15706 | 0.08084 | 1.80851 |
| O | 4.16120 | 0.82018 | 0.38727 |
| H | 2.26950 | 0.98451 | 2.12338 |
| H | 5.04613 | -0.58232 | 2.37238 |

**Single point energy: -1860.25817921 Hartrees**
**Gibbs free energy: -1860.02969 Hartrees**
**No. img. Freqs= 1**

| | | | |
|---|---|---|---|
| C | 1.04974 | -1.22991 | -1.67917 |
| C | 0.05455 | -0.18873 | -1.60704 |
| C | 0.15181 | 0.86649 | -0.64207 |
| C | 1.30488 | 0.95758 | 0.16155 |
| C | 2.39751 | 0.00456 | -0.06556 |
| C | 2.26371 | -1.01731 | -0.97425 |
| C | -0.89106 | 1.78419 | -0.48352 |
| C | 1.34237 | 1.94939 | 1.14764 |
| H | 3.07129 | -1.71273 | -1.16480 |
| C | 0.29260 | 2.84971 | 1.31254 |
| C | -0.82563 | 2.77896 | 0.49344 |
| H | 2.19301 | 2.01742 | 1.81814 |
| H | 0.34960 | 3.60197 | 2.09246 |
| H | -1.65009 | 3.47644 | 0.60566 |
| C | -2.04886 | 1.69047 | -1.35996 |
| O | -3.01941 | 2.38634 | -1.39833 |
| N | -1.01193 | -0.29604 | -2.39743 |
| O | -2.02648 | 0.65754 | -2.32024 |
| S | 3.86574 | 0.28917 | 0.82410 |
| C | 4.88888 | -1.14918 | 0.44226 |
| H | 5.77826 | -1.04048 | 1.06971 |
| H | 4.37081 | -2.07431 | 0.71100 |
| H | 5.19586 | -1.15996 | -0.60718 |
| O | -1.23795 | -1.14884 | -3.22408 |
| H | 1.07150 | -1.83375 | -2.57958 |
| O | 0.06690 | -2.62353 | -0.75996 |
| H | -0.06144 | -2.25187 | 0.20110 |
| H | 0.63091 | -3.40422 | -0.68546 |
| O | -0.27570 | -1.59708 | 1.46374 |
| P | 0.52755 | -1.59190 | 2.74608 |
| O | 1.90392 | -2.40176 | 2.36842 |
| O | -0.24140 | -2.61737 | 3.76025 |
| O | 0.85220 | -0.31963 | 3.46275 |
| H | -0.14433 | -2.33129 | 4.67511 |
| H | 2.59106 | -2.23260 | 3.02264 |

**Single point energy: -1860.25676209 Hartrees**
**Gibbs free energy: -1860.061254 Hartrees**
**No. img. Freqs= 1**

| | | | |
|---|---|---|---|
| C | 0.19255 | -0.62224 | -0.30205 |
| C | -1.22011 | -0.89294 | -0.27791 |
| C | -2.17617 | 0.15292 | -0.05927 |
| C | -1.72755 | 1.48755 | 0.02007 |
| C | -0.29632 | 1.76368 | -0.17462 |
| C | 0.59864 | 0.73962 | -0.35815 |
| C | -3.53848 | -0.14058 | 0.07173 |
| C | -2.67963 | 2.48596 | 0.26556 |
| H | 1.65472 | 0.92793 | -0.51854 |
| C | -4.03147 | 2.18415 | 0.40888 |
| C | -4.47057 | 0.86992 | 0.30595 |
| H | -2.37048 | 3.52395 | 0.34982 |
| H | -4.74266 | 2.98182 | 0.59741 |
| H | -5.52226 | 0.61882 | 0.40456 |
| C | -3.97435 | -1.52635 | -0.05147 |
| O | -5.08130 | -1.96872 | 0.02334 |
| N | -1.60981 | -2.16494 | -0.37747 |
| O | -2.97099 | -2.47845 | -0.30863 |
| S | 0.17413 | 3.44153 | -0.19959 |
| C | 1.97237 | 3.37951 | -0.37185 |
| H | 2.29543 | 4.42348 | -0.32846 |
| H | 2.43016 | 2.82408 | 0.45073 |
| H | 2.26086 | 2.95296 | -1.33608 |
| O | -0.91777 | -3.14021 | -0.52305 |
| H | 0.85484 | -1.36801 | -0.73319 |
| O | 0.69083 | -1.27048 | 1.47634 |
| H | 1.72072 | -1.21446 | 1.44110 |
| H | 0.41489 | -0.59451 | 2.10955 |
| O | 3.17042 | -1.01933 | 1.31075 |
| P | 4.01699 | -0.93956 | 0.06056 |
| O | 5.41041 | -1.47087 | -0.00041 |
| O | 3.99350 | 0.64806 | -0.35647 |
| O | 3.09134 | -1.62194 | -1.11367 |
| H | 4.74209 | 0.87255 | -0.92072 |
| H | 3.62982 | -2.00809 | -1.81363 |

**Single point energy: -1860.2608359 Hartrees**
**Gibbs free energy: -1860.064199 Hartrees**
**No. img. Freqs= 1**

| | | | |
|---|---|---|---|
| C | -1.39905 | -1.48951 | 1.92325 |
| C | -0.41981 | -0.43550 | 1.89298 |
| C | -0.38145 | 0.51586 | 0.82207 |
| C | -1.38787 | 0.48629 | -0.16282 |
| C | -2.48017 | -0.48278 | -0.01942 |
| C | -2.48134 | -1.39604 | 1.00656 |
| C | 0.65538 | 1.44950 | 0.73917 |
| C | -1.29178 | 1.38959 | -1.22895 |
| H | -3.29969 | -2.09228 | 1.14148 |
| C | -0.24585 | 2.30345 | -1.31417 |
| C | 0.73294 | 2.34109 | -0.32899 |
| H | -2.03426 | 1.37931 | -2.02166 |
| H | -0.19207 | 2.98108 | -2.15997 |
| H | 1.55857 | 3.04375 | -0.38389 |
| C | 1.67300 | 1.45924 | 1.78063 |
| O | 2.63932 | 2.15559 | 1.87653 |
| N | 0.50758 | -0.42028 | 2.85203 |
| O | 1.47397 | 0.57637 | 2.86018 |
| S | -3.77862 | -0.35781 | -1.17613 |
| C | -4.87838 | -1.72832 | -0.75598 |
| H | -5.67410 | -1.69043 | -1.50543 |
| H | -4.36033 | -2.68807 | -0.83638 |
| H | -5.32009 | -1.59777 | 0.23586 |
| O | 0.61678 | -1.18358 | 3.78176 |
| H | -1.56143 | -1.99075 | 2.87042 |
| O | -0.29504 | -2.99102 | 1.35391 |
| H | 0.15490 | -2.66746 | 0.47651 |
| H | -0.87544 | -3.72717 | 1.12288 |
| O | 0.73495 | -2.08384 | -0.71334 |
| P | 2.15946 | -1.58013 | -0.83659 |
| O | 3.13621 | -2.88141 | -1.00524 |
| O | 2.51843 | -1.08521 | 0.68605 |
| O | 2.51579 | -0.54680 | -1.85551 |
| H | 3.44225 | -0.82131 | 0.77664 |
| H | 3.77583 | -2.73507 | -1.71075 |

**Single point energy: -1860.26462644 Hartrees**
**Gibbs free energy: -1860.068314 Hartrees**
**No. img. Freqs= 1**

| | | | |
|---|---|---|---|
| C | 1.09244 | -1.13364 | -1.70355 |
| C | 0.05465 | -0.14954 | -1.55094 |
| C | 0.16884 | 0.92443 | -0.61014 |
| C | 1.35142 | 1.06068 | 0.14082 |
| C | 2.45232 | 0.12165 | -0.11187 |
| C | 2.31831 | -0.89467 | -1.02459 |
| C | -0.89781 | 1.80568 | -0.41603 |
| C | 1.41235 | 2.08987 | 1.08930 |
| H | 3.13739 | -1.56733 | -1.24572 |
| C | 0.34777 | 2.96679 | 1.27493 |
| C | -0.81342 | 2.83094 | 0.52249 |
| H | 2.29819 | 2.21292 | 1.70566 |
| H | 0.42525 | 3.75425 | 2.01791 |
| H | -1.65372 | 3.50482 | 0.65962 |
| C | -2.11095 | 1.62468 | -1.20638 |
| O | -3.11804 | 2.26567 | -1.17815 |
| N | -1.06364 | -0.31511 | -2.25737 |
| O | -2.06768 | 0.62917 | -2.20203 |
| S | 3.93152 | 0.41779 | 0.76225 |
| C | 4.98602 | -0.98127 | 0.32149 |
| H | 5.89272 | -0.85161 | 0.91929 |
| H | 4.51090 | -1.92806 | 0.59321 |
| H | 5.25443 | -0.96337 | -0.73863 |
| O | -1.31683 | -1.23635 | -3.00356 |
| H | 1.11819 | -1.69241 | -2.63256 |
| O | 0.40681 | -2.71942 | -0.81248 |
| H | 0.07067 | -2.40603 | 0.11472 |
| H | -0.36292 | -3.07054 | -1.28101 |
| O | -0.39631 | -1.88464 | 1.39864 |
| P | -1.74575 | -1.20595 | 1.50591 |
| O | -1.50441 | 0.02824 | 2.54099 |
| O | -2.72248 | -2.21696 | 2.33414 |
| O | -2.44828 | -0.73659 | 0.26350 |
| H | -3.63414 | -2.13935 | 2.03122 |
| H | -2.26921 | 0.61399 | 2.57983 |

**Single point energy: -1860.26052022 Hartrees**
**Gibbs free energy: -1860.063388 Hartrees**
**No. img. Freqs= 1**

| | | | |
|---|---|---|---|
| C | 1.12935 | -1.21444 | -1.67916 |
| C | 0.10605 | -0.20523 | -1.58711 |
| C | 0.20351 | 0.87071 | -0.64574 |
| C | 1.37222 | 1.00473 | 0.12840 |
| C | 2.48800 | 0.08482 | -0.12202 |
| C | 2.35985 | -0.95110 | -1.01469 |
| C | -0.85942 | 1.76205 | -0.47486 |
| C | 1.40831 | 2.01942 | 1.09333 |
| H | 3.18592 | -1.61774 | -1.22993 |
| C | 0.33522 | 2.88638 | 1.27574 |
| C | -0.80297 | 2.76760 | 0.48833 |
| H | 2.27950 | 2.13583 | 1.73154 |
| H | 0.38775 | 3.65222 | 2.04258 |
| H | -1.64970 | 3.43398 | 0.61993 |
| C | -2.04421 | 1.60547 | -1.30654 |
| O | -3.04947 | 2.25132 | -1.30707 |
| N | -0.98271 | -0.35394 | -2.34238 |
| O | -1.99260 | 0.59670 | -2.28925 |
| S | 3.97545 | 0.43196 | 0.71922 |

| | | | |
|---|---|---|---|
| C | 5.08413 | -0.90068 | 0.21266 |
| H | 6.01287 | -0.72093 | 0.76146 |
| H | 4.68487 | -1.87732 | 0.50072 |
| H | 5.29208 | -0.85865 | -0.86041 |
| O | -1.21585 | -1.24057 | -3.12990 |
| H | 1.14430 | -1.82254 | -2.57673 |
| O | 0.26846 | -2.66460 | -0.72961 |
| H | -0.05992 | -2.25016 | 0.16727 |
| H | 0.94944 | -3.31364 | -0.50972 |
| O | -0.48764 | -1.59403 | 1.37013 |
| P | -1.87010 | -1.05596 | 1.70374 |
| O | -2.77271 | -2.26569 | 2.32645 |
| O | -2.54892 | -0.81662 | 0.22424 |
| O | -2.03562 | 0.13415 | 2.58168 |
| H | -3.46106 | -0.50774 | 0.29359 |
| H | -2.59805 | -3.10480 | 1.88452 |

**Single point energy: -1860.25778807 Hartrees**
**Gibbs free energy: -1860.062113 Hartrees**
**No. img. Freqs= 1**

| | | | |
|---|---|---|---|
| C | -0.32145 | -1.04091 | -1.10270 |
| C | -1.65034 | -0.72342 | -0.64630 |
| C | -1.94734 | 0.55695 | -0.07345 |
| C | -0.95933 | 1.56297 | -0.09269 |
| C | 0.31410 | 1.28876 | -0.77725 |
| C | 0.58669 | 0.03682 | -1.27399 |
| C | -3.19794 | 0.80767 | 0.50257 |
| C | -1.26125 | 2.78876 | 0.51521 |
| H | 1.52210 | -0.18174 | -1.77415 |
| C | -2.50014 | 3.02315 | 1.10646 |
| C | -3.48002 | 2.03805 | 1.09538 |
| H | -0.52163 | 3.58399 | 0.53341 |
| H | -2.70003 | 3.98382 | 1.57021 |
| H | -4.45608 | 2.21090 | 1.53848 |
| C | -4.21272 | -0.23872 | 0.46256 |
| O | -5.33037 | -0.20861 | 0.88279 |
| N | -2.56394 | -1.69311 | -0.66359 |
| O | -3.84824 | -1.44154 | -0.16961 |
| S | 1.39517 | 2.63810 | -0.96309 |
| C | 2.77729 | 1.97540 | -1.92184 |
| H | 3.45508 | 2.82457 | -2.05013 |
| H | 3.29747 | 1.17536 | -1.38034 |
| H | 2.43581 | 1.64185 | -2.90626 |
| O | -2.43800 | -2.82165 | -1.07087 |
| H | -0.19913 | -1.92327 | -1.72078 |
| O | 0.28570 | -2.08318 | 0.44017 |
| H | 1.25052 | -2.32559 | 0.18561 |
| H | 0.41741 | -1.44783 | 1.15984 |
| O | 2.71047 | -2.42355 | -0.13949 |
| P | 3.60506 | -1.38495 | 0.50223 |
| O | 4.65675 | -2.18307 | 1.45664 |
| O | 2.60609 | -0.63949 | 1.57656 |
| O | 4.33720 | -0.36246 | -0.31280 |
| H | 2.99011 | 0.17151 | 1.92884 |
| H | 5.52049 | -1.75577 | 1.44978 |

**Single point energy: -1860.26017437 Hartrees**
**Gibbs free energy: -1860.064663 Hartrees**
**No. img. Freqs= 1**

| | | | |
|---|---|---|---|
| C | 0.96574 | -1.46901 | -1.14757 |
| C | 0.24544 | -0.22752 | -1.12595 |
| C | 0.72852 | 0.90241 | -0.39057 |
| C | 2.00178 | 0.83922 | 0.20874 |
| C | 2.82148 | -0.35901 | -0.01487 |
| C | 2.31515 | -1.44119 | -0.69102 |
| C | -0.06181 | 2.04641 | -0.25060 |
| C | 2.42498 | 1.93784 | 0.97084 |
| H | 2.91163 | -2.32594 | -0.87533 |
| C | 1.62333 | 3.06447 | 1.12248 |
| C | 0.37674 | 3.12815 | 0.50691 |
| H | 3.39317 | 1.92042 | 1.46295 |
| H | 1.97585 | 3.89776 | 1.72199 |
| H | -0.25662 | 4.00387 | 0.61138 |
| C | -1.35733 | 2.08757 | -0.92178 |
| O | -2.17347 | 2.95727 | -0.91876 |
| N | -0.92717 | -0.17223 | -1.76333 |
| O | -1.63664 | 1.01418 | -1.79719 |
| S | 4.46037 | -0.29432 | 0.58320 |
| C | 5.13650 | -1.91953 | 0.17605 |
| H | 6.15679 | -1.90848 | 0.56946 |
| H | 4.57385 | -2.71808 | 0.66772 |
| H | 5.17510 | -2.07293 | -0.90604 |
| O | -1.46658 | -1.06128 | -2.37828 |
| H | 0.69081 | -2.19857 | -1.90014 |
| O | 0.08297 | -2.58437 | 0.18879 |
| H | -0.94667 | -2.46602 | 0.30843 |
| H | 0.47212 | -2.32545 | 1.03431 |
| O | -2.36171 | -2.31906 | 0.56798 |
| P | -2.94570 | -0.99899 | 1.03650 |
| O | -3.45398 | -1.25831 | 2.55437 |
| O | -4.38429 | -0.74736 | 0.30828 |
| O | -2.08585 | 0.22795 | 0.92427 |
| H | -4.27526 | -0.28931 | -0.53332 |
| H | -3.81238 | -0.45781 | 2.95559 |

**Single point energy: -1860.25917078 Hartrees**
**Gibbs free energy: -1860.063348 Hartrees**
**No. img. Freqs= 1**

| | | | |
|---|---|---|---|
| C | 0.18486 | -0.60456 | -0.32417 |
| C | -1.21488 | -0.92042 | -0.20646 |

| | | | |
|---|---|---|---|
| C | -2.19753 | 0.10882 | -0.03026 |
| C | -1.79416 | 1.45911 | -0.08097 |
| C | -0.38334 | 1.76307 | -0.36647 |
| C | 0.54053 | 0.75842 | -0.51288 |
| C | -3.54094 | -0.21730 | 0.18749 |
| C | -2.77030 | 2.44304 | 0.12325 |
| H | 1.59141 | 0.95590 | -0.70967 |
| C | -4.10255 | 2.11026 | 0.35621 |
| C | -4.49776 | 0.77858 | 0.38276 |
| H | -2.49633 | 3.49406 | 0.10155 |
| H | -4.83320 | 2.89763 | 0.51085 |
| H | -5.53431 | 0.50235 | 0.55058 |
| C | -3.93152 | -1.62185 | 0.19483 |
| O | -5.01890 | -2.09166 | 0.34970 |
| N | -1.56088 | -2.20711 | -0.17707 |
| O | -2.90564 | -2.55911 | -0.02368 |
| S | 0.02883 | 3.44658 | -0.54539 |
| C | 1.80286 | 3.42076 | -0.89441 |
| H | 2.09115 | 4.47368 | -0.96145 |
| H | 2.36492 | 2.93628 | -0.09203 |
| H | 2.00472 | 2.92984 | -1.85019 |
| O | -0.83859 | -3.16868 | -0.26700 |
| H | 0.84170 | -1.36897 | -0.72714 |
| O | 0.78852 | -1.08456 | 1.47156 |
| H | 1.80107 | -1.24324 | 1.34009 |
| H | 0.70834 | -0.28317 | 2.00546 |
| O | 3.21383 | -1.46708 | 1.03426 |
| P | 4.16845 | -0.55037 | 0.29174 |
| O | 3.68308 | 0.73628 | -0.29931 |
| O | 4.86549 | -1.40186 | -0.91323 |
| O | 5.39274 | -0.29130 | 1.33894 |
| H | 5.07589 | -2.30217 | -0.63796 |
| H | 6.00414 | 0.37548 | 1.00455 |

**Single point energy: -1860.25842626 Hartrees**
**Gibbs free energy: -1860.063185 Hartrees**
**No. img. Freqs= 1**

| | | | |
|---|---|---|---|
| C | -0.21193 | -0.71999 | 0.41739 |
| C | 1.21496 | -0.92009 | 0.33575 |
| C | 2.11053 | 0.16846 | 0.07291 |
| C | 1.59249 | 1.47489 | -0.02780 |
| C | 0.15493 | 1.68225 | 0.21118 |
| C | -0.67835 | 0.62082 | 0.46411 |
| C | 3.47975 | -0.06360 | -0.09460 |
| C | 2.48390 | 2.51100 | -0.33108 |
| H | -1.73077 | 0.77022 | 0.68020 |
| C | 3.84508 | 2.27181 | -0.50873 |
| C | 4.35315 | 0.98522 | -0.38467 |
| H | 2.12054 | 3.52921 | -0.43572 |
| H | 4.50856 | 3.09810 | -0.74279 |
| H | 5.41251 | 0.78367 | -0.51146 |
| C | 3.98646 | -1.42335 | 0.04648 |
| O | 5.11327 | -1.81157 | -0.03078 |
| N | 1.66456 | -2.16808 | 0.40372 |
| O | 3.03292 | -2.42255 | 0.32219 |
| S | -0.40104 | 3.33069 | 0.20999 |
| C | -2.18961 | 3.18439 | 0.43147 |
| H | -2.56683 | 4.20799 | 0.35363 |
| H | -2.63640 | 2.56993 | -0.35409 |
| H | -2.43574 | 2.78237 | 1.41727 |
| O | 1.00692 | -3.18002 | 0.52474 |
| H | -0.80615 | -1.48252 | 0.91209 |
| O | -0.73377 | -1.36949 | -1.33530 |
| H | -1.72535 | -1.07129 | -1.40322 |
| H | -0.76239 | -2.33667 | -1.31697 |
| O | -3.12271 | -0.66282 | -1.39534 |
| P | -3.99044 | -0.71407 | -0.15503 |
| O | -3.33015 | -1.92951 | 0.72796 |
| O | -5.43483 | -1.30755 | -0.61442 |
| O | -4.17887 | 0.49878 | 0.70474 |
| H | -6.15111 | -0.88956 | -0.12355 |
| H | -3.70478 | -1.97235 | 1.61561 |

**Single point energy: -1860.25759901 Hartrees**
**Gibbs free energy: -1860.061922 Hartrees**
**No. img. Freqs= 1**

| | | | |
|---|---|---|---|
| C | -0.50380 | -1.09378 | -1.64560 |
| C | -1.66136 | -0.77568 | -0.84605 |
| C | -1.73191 | 0.43503 | -0.08244 |
| C | -0.69456 | 1.38050 | -0.19892 |
| C | 0.39611 | 1.11690 | -1.14888 |
| C | 0.45268 | -0.06714 | -1.84484 |
| C | -2.80809 | 0.66850 | 0.78037 |
| C | -0.76020 | 2.52619 | 0.60268 |
| H | 1.24357 | -0.26321 | -2.55716 |
| C | -1.82964 | 2.74900 | 1.46740 |
| C | -2.86422 | 1.82629 | 1.55645 |
| H | 0.03743 | 3.26255 | 0.56559 |
| H | -1.85058 | 3.64881 | 2.07391 |
| H | -3.70842 | 1.98942 | 2.21944 |
| C | -3.87997 | -0.31666 | 0.85138 |
| O | -4.88124 | -0.28367 | 1.50264 |
| N | -2.61451 | -1.69630 | -0.75352 |
| O | -3.73936 | -1.45593 | 0.03793 |
| S | 1.53445 | 2.40555 | -1.38955 |
| C | 2.80673 | 1.67125 | -2.43907 |
| H | 3.58084 | 2.43942 | -2.52273 |
| H | 3.23345 | 0.78923 | -1.95433 |
| H | 2.41890 | 1.44603 | -3.43649 |
| O | -2.64403 | -2.78336 | -1.28693 |

| | | | |
|---|---|---|---|
| H | -0.62554 | -1.83973 | -2.42340 |
| O | 0.29558 | -2.39952 | -0.44698 |
| H | 1.26834 | -2.08173 | -0.25522 |
| H | 0.35414 | -3.26802 | -0.86512 |
| O | 2.61584 | -1.63115 | -0.05209 |
| P | 3.11732 | -0.75394 | 1.07767 |
| O | 1.80129 | 0.08959 | 1.56543 |
| O | 4.04861 | 0.36998 | 0.34024 |
| O | 3.83760 | -1.31226 | 2.26399 |
| H | 4.71236 | 0.71612 | 0.94683 |
| H | 1.89168 | 0.36908 | 2.48313 |

**Single point energy: -1860.26383973 Hartrees**
**Gibbs free energy: -1860.066773 Hartrees**
**No. img. Freqs= 1**

| | | | |
|---|---|---|---|
| C | -1.04669 | -0.99904 | 1.84721 |
| C | 0.01807 | -0.06474 | 1.59026 |
| C | -0.09165 | 0.94023 | 0.57485 |
| C | -1.29429 | 1.06134 | -0.14633 |
| C | -2.41287 | 0.17625 | 0.20316 |
| C | -2.28231 | -0.77364 | 1.18715 |
| C | 0.99155 | 1.77394 | 0.28602 |
| C | -1.35788 | 2.02668 | -1.15862 |
| H | -3.11321 | -1.40392 | 1.47784 |
| C | -0.27608 | 2.85678 | -1.43899 |
| C | 0.90582 | 2.73456 | -0.71993 |
| H | -2.26075 | 2.13641 | -1.75230 |
| H | -0.35704 | 3.59364 | -2.23171 |
| H | 1.76174 | 3.36753 | -0.93283 |
| C | 2.22525 | 1.60835 | 1.04181 |
| O | 3.25846 | 2.19674 | 0.92446 |
| N | 1.15018 | -0.21674 | 2.27425 |
| O | 2.19613 | 0.67136 | 2.09478 |
| S | -3.90538 | 0.44818 | -0.65264 |
| C | -4.98628 | -0.88205 | -0.08218 |
| H | -5.90334 | -0.77143 | -0.66788 |
| H | -4.54409 | -1.86004 | -0.29178 |
| H | -5.22801 | -0.77591 | 0.97910 |
| O | 1.39888 | -1.08148 | 3.08743 |
| H | -1.05710 | -1.49582 | 2.81082 |
| O | -0.42178 | -2.66147 | 1.03404 |
| H | -0.11867 | -2.37581 | 0.08745 |
| H | 0.35786 | -3.01104 | 1.48665 |
| O | 0.25824 | -1.83165 | -1.21063 |
| P | 1.66905 | -1.48363 | -1.63573 |
| O | 2.40350 | -1.01127 | -0.23883 |
| O | 1.52490 | -0.09372 | -2.47479 |
| O | 2.55080 | -2.44642 | -2.36375 |
| H | 2.27069 | 0.02399 | -3.07393 |
| H | 3.35816 | -1.14052 | -0.28509 |

**Single point energy: -1860.25719316 Hartrees**
**Gibbs free energy: -1860.061774 Hartrees**
**No. img. Freqs= 1**

| | | | |
|---|---|---|---|
| C | 0.21766 | -0.67958 | -0.29707 |
| C | -1.20138 | -0.92214 | -0.27891 |
| C | -2.13413 | 0.14498 | -0.06094 |
| C | -1.65721 | 1.47010 | 0.01382 |
| C | -0.22296 | 1.71668 | -0.20002 |
| C | 0.64985 | 0.67258 | -0.38081 |
| C | -3.50149 | -0.11944 | 0.07792 |
| C | -2.58611 | 2.48838 | 0.26505 |
| H | 1.70974 | 0.84141 | -0.54301 |
| C | -3.94288 | 2.21521 | 0.41936 |
| C | -4.41030 | 0.91065 | 0.31900 |
| H | -2.25446 | 3.51980 | 0.34332 |
| H | -4.63553 | 3.02788 | 0.61289 |
| H | -5.46644 | 0.68180 | 0.42431 |
| C | -3.96772 | -1.49547 | -0.04453 |
| O | -5.08445 | -1.91296 | 0.03069 |
| N | -1.61781 | -2.18528 | -0.37017 |
| O | -2.98617 | -2.46901 | -0.30237 |
| S | 0.27989 | 3.38320 | -0.25138 |
| C | 2.06194 | 3.29039 | -0.54258 |
| H | 2.40424 | 4.32891 | -0.51750 |
| H | 2.57264 | 2.71802 | 0.23614 |
| H | 2.27317 | 2.86546 | -1.52750 |
| O | -0.94696 | -3.17680 | -0.50978 |
| H | 0.86416 | -1.44892 | -0.70990 |
| O | 0.66849 | -1.29952 | 1.49729 |
| H | 1.70263 | -1.33408 | 1.47663 |
| H | 0.44049 | -0.58258 | 2.10398 |
| O | 3.15930 | -1.30978 | 1.36465 |
| P | 3.96467 | -0.80855 | 0.18615 |
| O | 3.29771 | -1.57481 | -1.10128 |
| O | 5.44491 | -1.47465 | 0.30630 |
| O | 4.08309 | 0.65933 | -0.10169 |
| H | 6.12517 | -0.84202 | 0.04988 |
| H | 3.67279 | -1.26757 | -1.93488 |

**Single point energy: -1860.25834736 Hartrees**
**Gibbs free energy: -1860.061930 Hartrees**
**No. img. Freqs= 1**

| | | | |
|---|---|---|---|
| C | 0.16625 | -0.66145 | -0.40914 |
| C | -1.25188 | -0.91422 | -0.33819 |
| C | -2.18839 | 0.13756 | -0.06863 |
| C | -1.71876 | 1.46055 | 0.05162 |
| C | -0.28777 | 1.72260 | -0.17109 |
| C | 0.58494 | 0.69568 | -0.43017 |
| C | -3.54991 | -0.14517 | 0.08375 |
| C | -2.64949 | 2.46047 | 0.35848 |

| | | | |
|---|---|---|---|
| H | 1.63291 | 0.88280 | -0.63191 |
| C | -4.00273 | 2.17075 | 0.52032 |
| C | -4.46305 | 0.86825 | 0.37710 |
| H | -2.32374 | 3.48965 | 0.47875 |
| H | -4.69762 | 2.97006 | 0.75690 |
| H | -5.51563 | 0.62762 | 0.49131 |
| C | -4.00619 | -1.52022 | -0.07874 |
| O | -5.11865 | -1.94993 | -0.01628 |
| N | -1.65603 | -2.17709 | -0.42785 |
| O | -3.01473 | -2.48111 | -0.35884 |
| S | 0.20666 | 3.39120 | -0.14166 |
| C | 2.00319 | 3.31233 | -0.32649 |
| H | 2.34132 | 4.34793 | -0.22941 |
| H | 2.45393 | 2.70683 | 0.46398 |
| H | 2.28305 | 2.93277 | -1.31231 |
| O | -0.96243 | -3.16284 | -0.55910 |
| H | 0.79731 | -1.39195 | -0.90684 |
| O | 0.71561 | -1.30599 | 1.34129 |
| H | 1.70410 | -1.00236 | 1.37974 |
| H | 0.75038 | -2.27300 | 1.32960 |
| O | 3.10083 | -0.56669 | 1.31952 |
| P | 4.01853 | -0.77451 | 0.13376 |
| O | 5.40095 | -1.32073 | 0.27004 |
| O | 4.05624 | 0.67412 | -0.63208 |
| O | 3.13010 | -1.69967 | -0.89527 |
| H | 4.86845 | 0.77999 | -1.14011 |
| H | 3.68521 | -2.15326 | -1.53998 |

**Single point energy: -1860.25933828 Hartrees**
**Gibbs free energy: -1860.063837 Hartrees**
**No. img. Freqs= 1**

| | | | |
|---|---|---|---|
| C | -0.22166 | -0.68697 | 0.44778 |
| C | 1.19717 | -0.91592 | 0.32154 |
| C | 2.10937 | 0.15818 | 0.05288 |
| C | 1.61825 | 1.47761 | -0.01486 |
| C | 0.19265 | 1.71163 | 0.26421 |
| C | -0.65273 | 0.66452 | 0.52250 |
| C | 3.46887 | -0.09934 | -0.15238 |
| C | 2.52386 | 2.49943 | -0.32342 |
| H | -1.69121 | 0.82175 | 0.78093 |
| C | 3.87487 | 2.23446 | -0.53858 |
| C | 4.35742 | 0.93543 | -0.44731 |
| H | 2.17956 | 3.52665 | -0.40236 |
| H | 4.55040 | 3.05011 | -0.77548 |
| H | 5.40879 | 0.71333 | -0.60292 |
| C | 3.95013 | -1.47151 | -0.04589 |
| O | 5.06666 | -1.88100 | -0.15716 |
| N | 1.62209 | -2.17457 | 0.35591 |
| O | 2.98306 | -2.45534 | 0.23483 |
| S | -0.33355 | 3.37181 | 0.30452 |
| C | -2.11938 | 3.24689 | 0.56066 |
| H | -2.48331 | 4.27733 | 0.51811 |
| H | -2.59124 | 2.66400 | -0.23486 |
| H | -2.34909 | 2.82633 | 1.54343 |
| O | 0.94756 | -3.17462 | 0.47347 |
| H | -0.83489 | -1.42891 | 0.95248 |
| O | -0.81086 | -1.28965 | -1.30033 |
| H | -1.80276 | -0.99316 | -1.33896 |
| H | -0.83854 | -2.25709 | -1.29923 |
| O | -3.21999 | -0.60288 | -1.29478 |
| P | -3.96373 | -0.78043 | 0.01924 |
| O | -3.22471 | -1.26625 | 1.22544 |
| O | -5.24047 | -1.76316 | -0.21776 |
| O | -4.64766 | 0.67818 | 0.29852 |
| H | -5.66998 | -1.59591 | -1.06519 |
| H | -5.08983 | 0.69923 | 1.15561 |

## Intemediate after the phosphate catalysed water addition to C-7 of the naphtho oxazinium cation (B).

**Single point energy: -1860.28770933 Hartrees**
**Gibbs free energy: -1860.089601 Hartrees**
**No. img. Freqs= 0**

| | | | |
|---|---|---|---|
| C | -0.24825 | -1.11149 | -1.04289 |
| C | -1.60206 | -0.77039 | -0.48849 |
| C | -1.92718 | 0.54234 | 0.00286 |
| C | -1.01084 | 1.60179 | -0.17437 |
| C | 0.23648 | 1.35079 | -0.93458 |
| C | 0.55004 | 0.11616 | -1.36547 |
| C | -3.14469 | 0.76967 | 0.65409 |
| C | -1.34463 | 2.84822 | 0.35545 |
| H | 1.45430 | -0.07423 | -1.93467 |
| C | -2.55260 | 3.06212 | 1.02566 |
| C | -3.46355 | 2.02858 | 1.17014 |
| H | -0.65767 | 3.68311 | 0.25110 |
| H | -2.77638 | 4.04683 | 1.42446 |
| H | -4.41523 | 2.17804 | 1.67070 |
| C | -4.10048 | -0.32902 | 0.76816 |
| O | -5.18189 | -0.30411 | 1.29047 |
| N | -2.46300 | -1.75645 | -0.40347 |
| O | -3.73902 | -1.53302 | 0.18881 |
| S | 1.22971 | 2.78255 | -1.25170 |
| C | 2.67878 | 2.09734 | -2.08399 |
| H | 3.34276 | 2.94972 | -2.25594 |

| | | | |
|---|---|---|---|
| H | 3.19221 | 1.36977 | -1.44793 |
| H | 2.41428 | 1.65570 | -3.04957 |
| O | -2.33738 | -2.91332 | -0.77649 |
| H | -0.37416 | -1.72843 | -1.93868 |
| O | 0.46842 | -1.95261 | -0.12520 |
| H | 2.13666 | -2.31820 | -0.48539 |
| H | 0.71047 | -1.42518 | 0.65778 |
| O | 3.12163 | -2.27675 | -0.41313 |
| P | 3.55189 | -1.33632 | 0.79422 |
| O | 4.39675 | -0.15916 | 0.12743 |
| O | 4.71160 | -2.12010 | 1.56738 |
| O | 2.39933 | -0.88345 | 1.61175 |
| H | 4.39365 | -2.82208 | 2.15149 |
| H | 5.34110 | -0.16590 | 0.32979 |

**TS for the ring-opening of the intermediate B (TS-BC).**

**Single point energy: -1859.7813876 Hartrees**
**Gibbs free energy: -1859.601726 Hartrees**
**No. img. Freqs= 1**

| | | | |
|---|---|---|---|
| C | -0.75259 | -0.40737 | 1.88829 |
| C | 0.55801 | -1.18646 | 0.69140 |
| C | 1.53163 | -0.17275 | 0.32134 |
| C | 1.05460 | 1.15701 | 0.21074 |
| C | -0.40109 | 1.42962 | 0.28509 |
| C | -1.24307 | 0.64941 | 1.00150 |
| C | 2.89102 | -0.48117 | 0.16224 |
| C | 1.97345 | 2.17579 | -0.05078 |
| H | -2.32198 | 0.72264 | 0.91385 |
| C | 3.32560 | 1.88270 | -0.20064 |
| C | 3.77395 | 0.57155 | -0.09471 |
| H | 1.63449 | 3.20547 | -0.12199 |
| H | 4.03168 | 2.68554 | -0.39485 |
| H | 4.82910 | 0.33669 | -0.19761 |
| C | 3.45826 | -1.91243 | 0.24081 |
| O | 4.63566 | -2.00320 | 0.64150 |
| N | 0.35180 | -2.36365 | 0.66760 |
| O | 2.69015 | -2.83217 | -0.12619 |
| S | -0.94625 | 2.74417 | -0.74687 |
| C | -2.71414 | 2.84357 | -0.39030 |
| H | -3.08092 | 3.70251 | -0.95995 |
| H | -3.23699 | 1.94472 | -0.73038 |
| H | -2.89407 | 3.01614 | 0.67506 |
| O | -0.10908 | -3.43377 | 0.82348 |
| H | 0.06248 | -0.16167 | 2.57740 |
| O | -1.56855 | -1.29894 | 2.35681 |
| H | -2.44109 | -1.41310 | 1.76022 |
| O | -3.57752 | -1.50447 | 0.97085 |
| P | -3.62625 | -2.25816 | -0.35622 |
| O | -2.22413 | -1.83877 | -1.08734 |
| O | -4.77550 | -1.53152 | -1.25838 |
| O | -3.84804 | -3.72849 | -0.42288 |
| H | -1.96080 | -2.49151 | -1.74673 |
| H | -4.83255 | -0.58841 | -1.07046 |

**Single point energy: -1859.78627861 Hartrees**
**Gibbs free energy: -1859.605694 Hartrees**
**No. img. Freqs= 1**

| | | | |
|---|---|---|---|
| C | 0.09918 | -1.61397 | 0.06229 |
| C | 0.15776 | 0.11563 | 0.82749 |
| C | 1.11118 | 0.93724 | 0.10413 |
| C | 2.25808 | 0.28383 | -0.40629 |
| C | 2.51449 | -1.13429 | -0.05434 |
| C | 1.50479 | -1.98796 | 0.23625 |
| C | 0.85337 | 2.29076 | -0.14968 |
| C | 3.15277 | 1.02601 | -1.18004 |
| H | 1.67219 | -2.97711 | 0.65069 |
| C | 2.90855 | 2.37204 | -1.43954 |
| C | 1.77171 | 2.99280 | -0.93430 |
| H | 4.03322 | 0.54840 | -1.60031 |
| H | 3.60758 | 2.93531 | -2.05186 |
| H | 1.56704 | 4.03701 | -1.15151 |
| C | -0.37788 | 3.03226 | 0.39996 |
| O | -0.82180 | 3.95108 | -0.31427 |
| N | -0.75098 | 0.21509 | 1.60141 |
| O | -0.79484 | 2.65016 | 1.52080 |
| S | 4.21531 | -1.55928 | 0.07787 |
| C | 4.17273 | -3.33434 | 0.41199 |
| H | 5.21838 | -3.65568 | 0.42129 |
| H | 3.72942 | -3.54576 | 1.38965 |
| H | 3.63983 | -3.87048 | -0.37900 |
| O | -1.68722 | -0.05148 | 2.26960 |
| H | -0.20335 | -1.15797 | -0.89110 |
| O | -0.78660 | -2.37663 | 0.63022 |
| H | -1.74442 | -2.19409 | 0.19470 |
| O | -2.92016 | -1.92819 | -0.44863 |
| P | -3.55065 | -0.58563 | -0.80520 |
| O | -4.26332 | 0.03807 | 0.52024 |
| O | -2.24720 | 0.38555 | -1.01523 |
| O | -4.52034 | -0.49876 | -1.93100 |
| H | -3.66476 | 0.04890 | 1.27891 |
| H | -2.48901 | 1.29458 | -1.23334 |

**Single point energy: -1859.78215123 Hartrees**
**Gibbs free energy: -1859.601722 Hartrees**
**No. img. Freqs= 1**

| | | | |
|---|---|---|---|
| C | -0.85810 | -0.29459 | 1.56087 |
| C | 0.47442 | -0.83526 | 0.48687 |
| C | 1.54616 | 0.12390 | 0.28147 |
| C | 1.18525 | 1.48981 | 0.34398 |
| C | -0.24836 | 1.86943 | 0.44065 |
| C | -1.17771 | 1.02985 | 0.93543 |
| C | 2.87356 | -0.28379 | 0.07916 |
| C | 2.19798 | 2.44587 | 0.23731 |
| H | -2.23729 | 1.26842 | 0.91793 |
| C | 3.52206 | 2.05447 | 0.06103 |
| C | 3.85311 | 0.70697 | -0.02261 |
| H | 1.95738 | 3.50320 | 0.30161 |
| H | 4.29960 | 2.81008 | -0.01290 |
| H | 4.88332 | 0.39480 | -0.16322 |
| C | 3.30634 | -1.75104 | -0.06942 |
| O | 4.51090 | -1.99737 | 0.10430 |
| N | 0.32025 | -2.00630 | 0.20684 |
| O | 2.40537 | -2.57790 | -0.38552 |
| S | -0.63448 | 3.45660 | -0.24709 |
| C | -2.40258 | 3.62556 | 0.08257 |
| H | -2.67491 | 4.61911 | -0.28557 |
| H | -2.98766 | 2.87682 | -0.46055 |
| H | -2.61333 | 3.57227 | 1.15505 |
| O | -0.27941 | -3.02424 | 0.20337 |
| H | -0.18146 | -0.21965 | 2.43409 |
| O | -1.80130 | -1.16429 | 1.67179 |
| H | -2.83155 | -1.11817 | 0.69905 |
| O | -3.59083 | -1.08236 | -0.03417 |
| P | -3.20627 | -1.55117 | -1.48062 |
| O | -2.74370 | -3.08344 | -1.42211 |
| O | -1.78222 | -0.85299 | -1.81943 |
| O | -4.27349 | -1.33454 | -2.48129 |
| H | -1.94224 | -3.22928 | -0.89505 |
| H | -1.83539 | 0.10709 | -1.90334 |

**Single point energy: -1859.7799491 Hartrees**
**Gibbs free energy: -1859.600279 Hartrees**
**No. img. Freqs= 1**

| | | | |
|---|---|---|---|
| C | 1.53631 | -2.39277 | -0.60675 |
| C | 0.33786 | -1.28576 | -1.15032 |
| C | 0.19565 | -0.04246 | -0.39078 |
| C | 1.37097 | 0.42592 | 0.24150 |
| C | 2.67367 | -0.25396 | 0.00763 |
| C | 2.74477 | -1.52041 | -0.42941 |
| C | -1.01588 | 0.66229 | -0.28575 |
| C | 1.28401 | 1.56387 | 1.04657 |
| H | 3.68966 | -2.00637 | -0.65420 |
| C | 0.07814 | 2.24106 | 1.19367 |
| C | -1.05179 | 1.80595 | 0.51512 |
| H | 2.16242 | 1.92370 | 1.57408 |
| H | 0.02715 | 3.11924 | 1.83152 |
| H | -1.99188 | 2.34237 | 0.59412 |
| C | -2.30992 | 0.27331 | -1.00228 |
| O | -3.22146 | 1.10222 | -1.03392 |
| N | -0.37346 | -1.81460 | -1.99684 |
| O | -2.34697 | -0.89472 | -1.51652 |
| S | 4.09916 | 0.77027 | 0.27921 |
| C | 5.47363 | -0.36917 | 0.00875 |
| H | 6.37905 | 0.20994 | 0.21388 |
| H | 5.42827 | -1.21596 | 0.70053 |
| H | 5.50688 | -0.72014 | -1.02743 |
| O | -0.73076 | -2.57455 | -2.80995 |
| H | 1.64926 | -3.05930 | -1.47971 |
| O | 1.05025 | -2.94344 | 0.47709 |
| H | 0.04390 | -3.77895 | 0.19223 |
| O | -0.76526 | -4.43666 | -0.12097 |
| P | -2.22779 | -4.22310 | 0.39194 |
| O | -2.48309 | -2.64611 | 0.50538 |
| O | -2.25677 | -4.62809 | 1.95938 |
| O | -3.24907 | -5.00104 | -0.34895 |
| H | -2.49662 | -2.11422 | -0.32827 |
| H | -1.63763 | -4.11353 | 2.49154 |

**Single point energy: -1859.77993828 Hartrees**
**Gibbs free energy: -1859.601062 Hartrees**
**No. img. Freqs= 1**

| | | | |
|---|---|---|---|
| C | 0.99831 | 2.24134 | 0.82078 |
| C | 0.39052 | 0.64267 | 1.28762 |
| C | 0.75182 | -0.44331 | 0.37497 |
| C | 1.97236 | -0.28648 | -0.32157 |
| C | 2.88861 | 0.83509 | 0.01697 |
| C | 2.44670 | 1.96144 | 0.60088 |
| C | -0.05563 | -1.57617 | 0.18933 |
| C | 2.31518 | -1.23964 | -1.28139 |
| H | 3.11606 | 2.74551 | 0.94229 |
| C | 1.49522 | -2.34079 | -1.51142 |
| C | 0.34168 | -2.52055 | -0.76085 |
| H | 3.22568 | -1.12208 | -1.86145 |
| H | 1.77262 | -3.06976 | -2.26791 |
| H | -0.27869 | -3.40109 | -0.89679 |
| C | -1.33254 | -1.86864 | 0.98603 |
| O | -1.59932 | -3.05981 | 1.18148 |
| N | -0.40636 | 0.77164 | 2.19421 |
| O | -2.00053 | -0.86589 | 1.38130 |
| S | 4.59735 | 0.51065 | -0.31273 |
| C | 5.39294 | 2.08211 | 0.08643 |

| | | | |
|---|---|---|---|
| H | 6.44807 | 1.95066 | -0.17144 |
| H | 4.97788 | 2.89766 | -0.51377 |
| H | 5.31711 | 2.31127 | 1.15388 |
| O | -1.04602 | 1.15250 | 3.09140 |
| H | 0.80894 | 2.82263 | 1.73493 |
| O | 0.29857 | 2.58182 | -0.23091 |
| H | -0.78061 | 2.89272 | 0.09549 |
| O | -1.87392 | 3.18178 | 0.54134 |
| P | -3.18266 | 2.44539 | 0.15659 |
| O | -2.78650 | 0.97119 | -0.34300 |
| O | -3.70480 | 3.09227 | -1.24270 |
| O | -4.28357 | 2.49833 | 1.15477 |
| H | -2.51932 | 0.31575 | 0.35574 |
| H | -3.00987 | 3.12513 | -1.91014 |

**Single point energy: -1859.78290401 Hartrees**
**Gibbs free energy: -1859.601719 Hartrees**
**No. img. Freqs= 1**

| | | | |
|---|---|---|---|
| C | 0.10190 | 1.84550 | 0.60431 |
| C | 0.22986 | 0.14717 | 1.05458 |
| C | 1.12823 | -0.63196 | 0.19955 |
| C | 2.24791 | 0.05034 | -0.31955 |
| C | 2.51196 | 1.45689 | 0.08501 |
| C | 1.53480 | 2.25211 | 0.55679 |
| C | 0.87651 | -1.97788 | -0.09499 |
| C | 3.09613 | -0.64297 | -1.18393 |
| H | 1.72993 | 3.24704 | 0.94552 |
| C | 2.85584 | -1.98100 | -1.48957 |
| C | 1.77102 | -2.64712 | -0.93359 |
| H | 3.94622 | -0.13750 | -1.63248 |
| H | 3.52699 | -2.50495 | -2.16454 |
| H | 1.59537 | -3.69686 | -1.15036 |
| C | -0.33420 | -2.73871 | 0.44915 |
| O | -0.12536 | -3.86146 | 0.92078 |
| N | -0.58125 | -0.13446 | 1.90637 |
| O | -1.44421 | -2.13356 | 0.35212 |
| S | 4.20581 | 1.94830 | -0.01974 |
| C | 4.14520 | 3.70726 | 0.38699 |
| H | 5.16910 | 4.07219 | 0.26327 |
| H | 3.48773 | 4.24759 | -0.30088 |
| H | 3.83658 | 3.86952 | 1.42435 |
| O | -1.38745 | -0.22238 | 2.74148 |
| H | -0.43691 | 2.26987 | 1.46133 |
| O | -0.55149 | 1.84553 | -0.53690 |
| H | -1.59144 | 1.57058 | -0.32722 |
| O | -2.74305 | 1.14297 | 0.14657 |
| P | -3.66568 | 0.08116 | -0.48377 |
| O | -3.98762 | -1.02080 | 0.66786 |
| O | -2.78117 | -0.75011 | -1.56290 |
| O | -4.93882 | 0.54817 | -1.10062 |
| H | -3.19141 | -1.54042 | 0.88165 |
| H | -2.09946 | -1.26748 | -1.08270 |

**Single point energy: -1859.782904032 Hartrees**
**Gibbs free energy: -1859.601705 Hartrees**
**No. img. Freqs= 1**

| | | | |
|---|---|---|---|
| C | 0.55763 | 1.78168 | 0.50563 |
| C | 0.22429 | 0.14020 | 1.05093 |
| C | 0.87181 | -0.89974 | 0.24820 |
| C | 2.13063 | -0.57632 | -0.29902 |
| C | 2.76858 | 0.72661 | 0.02836 |
| C | 2.04673 | 1.78189 | 0.44680 |
| C | 0.26287 | -2.14153 | 0.02732 |
| C | 2.75305 | -1.52066 | -1.11640 |
| H | 2.50680 | 2.70720 | 0.78029 |
| C | 2.15709 | -2.75851 | -1.34886 |
| C | 0.93648 | -3.07376 | -0.76543 |
| H | 3.70502 | -1.29026 | -1.58546 |
| H | 2.65628 | -3.48154 | -1.98809 |
| H | 0.48144 | -4.04707 | -0.92483 |
| C | -1.10473 | -2.51439 | 0.60301 |
| O | -1.20454 | -3.62371 | 1.13784 |
| N | -0.62689 | 0.13672 | 1.91016 |
| O | -2.00993 | -1.63748 | 0.46361 |
| S | 4.53142 | 0.73440 | -0.08988 |
| C | 4.95227 | 2.46408 | 0.21692 |
| H | 6.03577 | 2.53066 | 0.08113 |
| H | 4.46052 | 3.12262 | -0.50550 |
| H | 4.70685 | 2.76192 | 1.24101 |
| O | -1.42115 | 0.31731 | 2.74180 |
| H | 0.16022 | 2.38309 | 1.33344 |
| O | -0.07972 | 1.89492 | -0.63902 |
| H | -1.15373 | 1.92364 | -0.42311 |
| O | -2.37504 | 1.85043 | 0.06311 |
| P | -3.55530 | 1.04458 | -0.51440 |
| O | -2.93674 | -0.05458 | -1.53772 |
| O | -4.15571 | 0.13768 | 0.69441 |
| O | -4.65859 | 1.80351 | -1.16732 |
| H | -2.41711 | -0.70918 | -1.02344 |
| H | -3.52825 | -0.56488 | 0.94417 |

**Single point energy: -1859.78343505 Hartrees**
**Gibbs free energy: -1859.603321 Hartrees**
**No. img. Freqs= 1**

| | | | |
|---|---|---|---|
| C | -0.37337 | 0.54640 | 1.59744 |
| C | 0.24351 | -0.71687 | 0.29641 |
| C | 1.67185 | -0.53555 | 0.09793 |
| C | 2.16049 | 0.79043 | 0.20748 |
| C | 1.21185 | 1.92508 | 0.33292 |
| C | -0.00465 | 1.77945 | 0.91035 |

| | | | |
|---|---|---|---|
| C | 2.53062 | -1.62368 | -0.11388 |
| C | 3.53805 | 1.00032 | 0.11606 |
| H | -0.77659 | 2.54080 | 0.85968 |
| C | 4.40157 | -0.07275 | -0.08197 |
| C | 3.90139 | -1.36425 | -0.19596 |
| H | 3.94094 | 2.00374 | 0.22014 |
| H | 5.47250 | 0.10263 | -0.13826 |
| H | 4.56961 | -2.20840 | -0.33705 |
| C | 2.05094 | -3.08098 | -0.27169 |
| O | 2.88451 | -3.95929 | 0.01984 |
| N | -0.65529 | -1.47074 | 0.05690 |
| O | 0.88142 | -3.24025 | -0.70075 |
| S | 1.74202 | 3.40924 | -0.44636 |
| C | 0.44854 | 4.59204 | -0.00643 |
| H | 0.79089 | 5.55772 | -0.38990 |
| H | -0.50333 | 4.33957 | -0.48331 |
| H | 0.33159 | 4.65872 | 1.07944 |
| O | -1.71278 | -1.98609 | 0.01083 |
| H | 0.33894 | 0.09892 | 2.30941 |
| O | -1.63517 | 0.38044 | 1.87793 |
| H | -1.72071 | -0.41966 | 2.55321 |
| O | -1.67854 | -1.53324 | 3.43067 |
| P | -0.38431 | -2.19193 | 3.90702 |
| O | -0.32969 | -3.71438 | 3.33115 |
| O | -0.54575 | -2.50029 | 5.49091 |
| O | 0.90163 | -1.47058 | 3.65057 |
| H | 0.09671 | -3.75881 | 2.46623 |

## Structure of the aldehyde C obtained after the aperture reaction

**Single point energy: -1859.83185441 Hartrees**
**Gibbs free energy: -1859.659907 Hartrees**
**No. img. Freqs= 0**

| | | | |
|---|---|---|---|
| C | 0.68247 | -1.73412 | 1.59809 |
| C | 0.21162 | 0.24990 | -1.05973 |
| C | 0.95436 | 1.11003 | -0.18467 |
| C | 2.10793 | 0.58490 | 0.43542 |
| C | 2.51715 | -0.83319 | 0.20370 |
| C | 1.84612 | -1.88844 | 0.72354 |
| C | 0.54351 | 2.43234 | 0.05116 |
| C | 2.86241 | 1.40580 | 1.27157 |
| H | 2.14513 | -2.91299 | 0.51841 |
| C | 2.46978 | 2.72377 | 1.49626 |
| C | 1.32532 | 3.22924 | 0.88677 |
| H | 3.74745 | 1.00484 | 1.75846 |
| H | 3.05745 | 3.35569 | 2.15639 |
| H | 1.01475 | 4.25570 | 1.05782 |
| C | -0.74243 | 3.00616 | -0.54487 |
| O | -0.76107 | 4.21504 | -0.79612 |
| N | -0.35791 | -0.50676 | -1.71953 |
| O | -1.69478 | 2.18110 | -0.70358 |
| S | 3.93419 | -0.94085 | -0.82016 |
| C | 4.24783 | -2.71703 | -0.93667 |
| H | 5.13552 | -2.81848 | -1.56801 |
| H | 4.45730 | -3.14502 | 0.04831 |
| H | 3.40996 | -3.23409 | -1.41376 |
| O | -0.96220 | -1.30960 | -2.41959 |
| H | 0.36184 | -0.70327 | 1.83824 |
| O | 0.08974 | -2.68492 | 2.08090 |
| H | -2.32403 | -1.79953 | -1.35831 |
| O | -2.85430 | -2.02642 | -0.56347 |
| P | -3.52079 | -0.76951 | 0.17763 |
| O | -2.33252 | 0.16389 | 0.61526 |
| O | -4.01236 | -1.46121 | 1.54394 |
| O | -4.64521 | -0.12745 | -0.54085 |
| H | -2.11577 | 1.01811 | 0.06128 |
| H | -3.29428 | -1.89706 | 2.02072 |

## TSs for the cyclization reaction to obtain product D (TS-CD)

**Single point energy: -1859.81493399 Hartrees**
**Gibbs free energy: -1859.636941 Hartrees**
**No. img. Freqs= 1**

| | | | |
|---|---|---|---|
| C | 0.75460 | -1.34414 | 2.27196 |
| C | -1.74124 | 1.23545 | 0.32607 |
| C | -1.78124 | -0.13275 | -0.14301 |
| C | -0.64041 | -0.93549 | -0.29750 |
| C | 0.73319 | -0.41837 | -0.01286 |
| C | 1.37339 | -0.62986 | 1.16213 |
| C | -3.05347 | -0.62348 | -0.42739 |
| C | -0.81918 | -2.24325 | -0.75555 |
| H | 2.38192 | -0.26332 | 1.33402 |
| C | -2.09382 | -2.73682 | -1.03978 |
| C | -3.21693 | -1.92782 | -0.87714 |
| H | 0.05323 | -2.87881 | -0.88557 |
| H | -2.20419 | -3.75948 | -1.39024 |
| H | -4.21586 | -2.29577 | -1.09446 |
| C | -4.16847 | 0.37626 | -0.20157 |

| | | | |
|---|---|---|---|
| O | -5.34255 | 0.06373 | -0.42087 |
| N | -1.04328 | 2.11917 | 0.68160 |
| O | -3.73066 | 1.50024 | 0.21622 |
| S | 1.39570 | 0.44848 | -1.37946 |
| C | 3.00575 | 1.01991 | -0.78828 |
| H | 3.41134 | 1.64342 | -1.58991 |
| H | 2.89491 | 1.62912 | 0.11288 |
| H | 3.67939 | 0.17650 | -0.60921 |
| O | -0.76761 | 3.29105 | 1.08024 |
| H | -0.29184 | -1.67584 | 2.13228 |
| O | 1.33326 | -1.58027 | 3.32148 |
| H | 0.84284 | 3.34770 | 1.43427 |
| O | 1.82113 | 3.52757 | 1.32949 |
| P | 2.09085 | 4.65819 | 0.25575 |
| O | 1.79415 | 5.99594 | 1.10590 |
| O | 0.90682 | 4.55206 | -0.82003 |
| O | 3.42205 | 4.62635 | -0.38305 |
| H | 2.04906 | 6.79964 | 0.63485 |
| H | 0.05979 | 4.28331 | -0.41446 |

**Single point energy: -1859.8117271 Hartrees**
**Gibbs free energy: -1859.633331 Hartrees**
**No. img. Freqs= 1**

| | | | |
|---|---|---|---|
| C | -2.03320 | 2.31170 | 1.39953 |
| C | 0.21079 | -0.87987 | 0.56361 |
| C | 0.70555 | 0.36028 | -0.00770 |
| C | -0.08861 | 1.34669 | -0.60298 |
| C | -1.56596 | 1.16253 | -0.72970 |
| C | -2.45160 | 1.61320 | 0.18963 |
| C | 2.08462 | 0.51609 | 0.09405 |
| C | 0.55034 | 2.48631 | -1.09810 |
| H | -3.52160 | 1.46644 | 0.06986 |
| C | 1.93572 | 2.64070 | -0.99284 |
| C | 2.71637 | 1.65219 | -0.39498 |
| H | -0.04791 | 3.26349 | -1.56758 |
| H | 2.40255 | 3.54062 | -1.38341 |
| H | 3.79420 | 1.75795 | -0.30830 |
| C | 2.71884 | -0.66212 | 0.76695 |
| O | 3.91689 | -0.77893 | 0.97455 |
| N | -0.67988 | -1.61859 | 0.83152 |
| O | 1.79576 | -1.52379 | 1.08993 |
| S | -1.97732 | 0.29990 | -2.19436 |
| C | -3.76429 | 0.06264 | -2.06418 |
| H | -4.05075 | -0.51853 | -2.94552 |
| H | -4.01052 | -0.50704 | -1.16348 |
| H | -4.29462 | 1.01943 | -2.07842 |
| O | -1.88796 | -2.03620 | 0.89740 |
| H | -0.94414 | 2.41657 | 1.56542 |
| O | -2.82025 | 2.77193 | 2.21168 |
| H | -1.64054 | -3.33655 | 1.60407 |
| O | -1.37673 | -4.21389 | 2.07543 |
| P | -0.02185 | -4.29355 | 2.86233 |
| O | -0.07104 | -2.95230 | 3.76892 |
| O | 1.20456 | -4.07390 | 1.85999 |
| O | 0.14538 | -5.55763 | 3.61673 |
| H | 0.69095 | -2.87340 | 4.35623 |
| H | 1.41042 | -3.15118 | 1.59129 |

## Structure of product D:

**Single point energy: -1859.86873982 Hartrees**
**Gibbs free energy: -1859.685891 Hartrees**
**No. img. Freqs= 0**

| | | | |
|---|---|---|---|
| C | 1.23352 | 2.68689 | 1.86009 |
| C | 1.19704 | -1.24413 | 0.55123 |
| C | 2.12847 | -0.28975 | -0.04631 |
| C | 1.98923 | 1.03832 | -0.45824 |
| C | 0.68851 | 1.76786 | -0.35881 |
| C | 0.35724 | 2.53560 | 0.70644 |
| C | 3.35051 | -0.94727 | -0.15576 |
| C | 3.12407 | 1.65541 | -0.99226 |
| H | -0.59327 | 3.05959 | 0.75535 |
| C | 4.35028 | 0.98614 | -1.10207 |
| C | 4.48174 | -0.33424 | -0.68173 |
| H | 3.04804 | 2.68808 | -1.32427 |
| H | 5.20467 | 1.51121 | -1.51923 |
| H | 5.42528 | -0.86679 | -0.75771 |
| C | 3.17142 | -2.31330 | 0.37297 |
| O | 3.94548 | -3.22515 | 0.47347 |
| N | -0.02946 | -1.04069 | 0.81232 |
| O | 1.86417 | -2.43249 | 0.78356 |
| S | -0.30855 | 1.50800 | -1.76896 |
| C | -1.87923 | 2.28424 | -1.32862 |
| H | -2.55332 | 2.08207 | -2.16488 |
| H | -2.29547 | 1.82825 | -0.42625 |
| H | -1.76539 | 3.36605 | -1.21092 |
| O | -0.68341 | -2.08750 | 1.35553 |
| H | 2.17386 | 2.10253 | 1.85345 |
| O | 0.97941 | 3.41387 | 2.80816 |
| H | -1.63557 | -1.76233 | 1.41506 |
| O | -3.08778 | -1.23714 | 1.38745 |
| P | -3.99553 | -0.75889 | 0.27773 |

| | | | |
|---|---|---|---|
| O | -5.10255 | -1.95716 | 0.08956 |
| O | -3.16259 | -0.80318 | -1.13596 |
| O | -4.68262 | 0.56824 | 0.32893 |
| H | -5.85698 | -1.64159 | -0.41987 |
| H | -2.50298 | -1.50554 | -1.14303 |

**Dimethylamine catalysed water addition to carbonyl C of the naphtho oxazinium cation (A) to yield carboxylate E (TS-AE analogue).**

**Single point energy: -1351.76729244 Hartrees**
**Gibbs free energy: -1351.515088 Hartrees**
**No. img. Freqs= 1**

| | | | |
|---|---|---|---|
| C | 0.60429 | -1.27292 | -1.59601 |
| C | -0.44567 | -0.41072 | -1.27701 |
| C | -0.23739 | 0.74931 | -0.47520 |
| C | 1.08100 | 1.08496 | -0.08648 |
| C | 2.15782 | 0.19032 | -0.43992 |
| C | 1.88458 | -0.98458 | -1.15766 |
| C | -1.31829 | 1.52232 | -0.01712 |
| C | 1.27040 | 2.26810 | 0.67195 |
| H | 2.67878 | -1.67426 | -1.41554 |
| C | 0.20098 | 3.04946 | 1.05113 |
| C | -1.11174 | 2.66245 | 0.72979 |
| H | 2.27037 | 2.57293 | 0.96646 |
| H | 0.36983 | 3.95592 | 1.62367 |
| H | -1.96232 | 3.24232 | 1.07615 |
| C | -2.68008 | 1.01315 | -0.26050 |
| O | -3.73926 | 1.49598 | -0.03612 |
| N | -1.70624 | -0.68231 | -1.78924 |
| O | -2.69714 | 0.14984 | -1.55138 |
| S | 3.75963 | 0.61809 | 0.07014 |
| C | 4.82248 | -0.67623 | -0.61100 |
| H | 5.83337 | -0.39222 | -0.30499 |
| H | 4.58536 | -1.65490 | -0.18583 |
| H | 4.77303 | -0.69262 | -1.70298 |
| O | -1.95211 | -1.61530 | -2.50268 |
| H | 0.41835 | -2.15871 | -2.19502 |
| O | -2.47497 | -0.63277 | 0.82976 |
| H | -1.50157 | -1.15201 | 1.35777 |
| H | -3.29263 | -0.71959 | 1.32940 |
| N | -0.50015 | -1.74283 | 1.84151 |
| C | -0.56075 | -3.15612 | 1.43482 |
| H | -0.53556 | -3.21326 | 0.34307 |
| H | 0.27512 | -3.72688 | 1.85387 |
| H | -1.50294 | -3.58517 | 1.78746 |
| C | -0.50353 | -1.55113 | 3.30083 |
| H | 0.33375 | -2.07643 | 3.77278 |
| H | -0.43670 | -0.48207 | 3.51960 |
| H | -1.44372 | -1.93804 | 3.70449 |

| | | | |
|---|---|---|---|
| H | 0.35141 | -1.33060 | 1.45910 |

**Single point energy: -1351.76886251 Hartrees**
**Gibbs free energy: -1351.514773 Hartrees**
**No. img. Freqs= 1**

| | | | |
|---|---|---|---|
| C | 0.69274 | -1.11175 | -1.88165 |
| C | -0.38270 | -0.32906 | -1.46331 |
| C | -0.22432 | 0.67658 | -0.46559 |
| C | 1.07471 | 0.96425 | 0.01621 |
| C | 2.17946 | 0.15342 | -0.44039 |
| C | 1.95328 | -0.88485 | -1.35546 |
| C | -1.33335 | 1.34931 | 0.07712 |
| C | 1.21873 | 2.01669 | 0.95549 |
| H | 2.76858 | -1.51056 | -1.69845 |
| C | 0.12405 | 2.71476 | 1.41633 |
| C | -1.17151 | 2.36137 | 0.99937 |
| H | 2.20319 | 2.28269 | 1.32881 |
| H | 0.25814 | 3.52231 | 2.12898 |
| H | -2.04309 | 2.86592 | 1.40642 |
| C | -2.67812 | 0.87220 | -0.29675 |
| O | -3.75178 | 1.30524 | -0.04317 |
| N | -1.61527 | -0.50639 | -2.08023 |
| O | -2.62562 | 0.25877 | -1.73886 |
| S | 3.75434 | 0.51343 | 0.19477 |
| C | 4.85796 | -0.66939 | -0.61095 |
| H | 5.85105 | -0.43337 | -0.21812 |
| H | 4.60616 | -1.69845 | -0.34136 |
| H | 4.86196 | -0.53113 | -1.69550 |
| O | -1.80895 | -1.28720 | -2.97075 |
| H | 0.54521 | -1.88455 | -2.62941 |
| O | -2.51326 | -0.93124 | 0.45427 |
| H | -1.57566 | -1.36395 | 1.05930 |
| H | -3.35654 | -1.10329 | 0.88623 |
| N | -0.53917 | -1.82605 | 1.66872 |
| C | -0.74631 | -3.25393 | 1.95704 |
| H | -0.88097 | -3.79308 | 1.01562 |
| H | 0.10242 | -3.67778 | 2.50554 |
| H | -1.65268 | -3.36551 | 2.55960 |
| C | -0.31733 | -1.01053 | 2.87201 |
| H | 0.55264 | -1.36044 | 3.43915 |
| H | -0.16541 | 0.02988 | 2.57321 |
| H | -1.20616 | -1.06866 | 3.50740 |

| | | | |
|---|---|---|---|
| H | 0.26970 | -1.73058 | 1.05443 |

**Single point energy: -1351.77084715 Hartrees**
**Gibbs free energy: -1351.518398 Hartrees**
**No. img. Freqs= 1**

| | | | |
|---|---|---|---|
| C | 1.12696 | 1.85659 | -1.11321 |
| C | 0.01148 | 1.56860 | -0.32500 |
| C | 0.03045 | 0.51227 | 0.63160 |
| C | 1.24063 | -0.18848 | 0.84960 |
| C | 2.39156 | 0.13862 | 0.03925 |
| C | 2.29708 | 1.14026 | -0.93744 |
| C | -1.12396 | 0.15142 | 1.34784 |
| C | 1.25734 | -1.19660 | 1.84498 |
| H | 3.14739 | 1.39267 | -1.55854 |
| C | 0.12684 | -1.49941 | 2.57296 |
| C | -1.08261 | -0.83341 | 2.31298 |
| H | 2.17193 | -1.74613 | 2.04776 |
| H | 0.16560 | -2.26756 | 3.33865 |
| H | -1.98541 | -1.08937 | 2.85999 |
| C | -2.40089 | 0.80836 | 1.00584 |
| O | -3.45039 | 0.77716 | 1.56122 |
| N | -1.09839 | 2.39096 | -0.42763 |
| O | -2.11794 | 2.21813 | 0.39057 |
| S | 3.85379 | -0.74996 | 0.32210 |
| C | 5.03641 | -0.07725 | -0.86863 |
| H | 5.95659 | -0.64159 | -0.69295 |
| H | 4.70470 | -0.24708 | -1.89659 |
| H | 5.22754 | 0.98259 | -0.68065 |
| O | -1.17498 | 3.32421 | -1.17961 |
| H | 1.08266 | 2.65648 | -1.84491 |
| O | -2.58521 | 0.08260 | -0.78783 |
| H | -2.48215 | -1.07578 | -0.94644 |
| H | -3.47188 | 0.35789 | -1.05091 |
| N | -2.30058 | -2.36455 | -1.13280 |
| C | -3.03614 | -3.11900 | -0.10797 |
| H | -4.10016 | -2.87793 | -0.17783 |
| H | -2.89887 | -4.20002 | -0.22857 |
| H | -2.67307 | -2.82143 | 0.88072 |
| C | -0.85835 | -2.64793 | -1.13472 |
| H | -0.65761 | -3.70671 | -1.33638 |
| H | -0.37181 | -2.03430 | -1.89824 |
| H | -0.44636 | -2.39050 | -0.15484 |
| H | -2.68069 | -2.59744 | -2.04850 |

**Single point energy: -1351.77045343 Hartrees**
**Gibbs free energy: -1351.519081 Hartrees**
**No. img. Freqs= 1**

| | | | |
|---|---|---|---|
| C | 1.19703 | -2.01543 | -0.32515 |
| C | 0.29836 | -0.96767 | -0.56229 |
| C | 0.67473 | 0.39270 | -0.36319 |
| C | 2.01026 | 0.68835 | -0.00575 |
| C | 2.93993 | -0.40410 | 0.17363 |
| C | 2.49672 | -1.73212 | 0.04122 |
| C | -0.26638 | 1.42794 | -0.48215 |
| C | 2.36383 | 2.04655 | 0.18282 |
| H | 3.17560 | -2.56008 | 0.20398 |
| C | 1.43182 | 3.05224 | 0.03536 |
| C | 0.10093 | 2.74525 | -0.28867 |
| H | 3.38072 | 2.31293 | 0.45583 |
| H | 1.72484 | 4.08672 | 0.18368 |
| H | -0.64276 | 3.53145 | -0.38242 |
| C | -1.66692 | 1.07125 | -0.77034 |
| O | -2.58942 | 1.77869 | -1.03389 |
| N | -0.95395 | -1.26968 | -1.04033 |
| O | -1.77660 | -0.28449 | -1.40966 |
| S | 4.58130 | -0.00825 | 0.54824 |
| C | 5.41661 | -1.60308 | 0.71249 |
| H | 6.45581 | -1.34860 | 0.93965 |
| H | 5.00296 | -2.18203 | 1.54221 |
| H | 5.38425 | -2.16592 | -0.22420 |
| O | -1.36306 | -2.38034 | -1.24140 |
| H | 0.87755 | -3.04442 | -0.45492 |
| O | -2.04723 | 0.14909 | 1.02320 |
| H | -3.29559 | 0.00577 | 0.95707 |
| H | -1.82269 | 0.74387 | 1.74596 |
| N | -4.50469 | -0.19209 | 0.83452 |
| C | -5.11442 | -0.29220 | 2.16966 |
| H | -4.93398 | 0.63703 | 2.71654 |
| H | -6.19273 | -0.47061 | 2.09930 |
| H | -4.64609 | -1.12044 | 2.70933 |
| C | -4.71218 | -1.39250 | 0.00783 |
| H | -5.77920 | -1.61887 | -0.09342 |
| H | -4.27900 | -1.22504 | -0.98065 |
| H | -4.20637 | -2.23844 | 0.48207 |
| H | -4.89083 | 0.61550 | 0.34768 |

**Single point energy: -1351.77046535 Hartrees**
**Gibbs free energy: -1351.519315 Hartrees**
**No. img. Freqs= 1**

| | | | |
|---|---|---|---|
| C | -1.47394 | 0.21651 | -0.01159 |
| C | -0.08891 | 0.01322 | -0.04466 |
| C | 0.47107 | -1.20653 | -0.52559 |
| C | -0.39255 | -2.20100 | -1.04089 |
| C | -1.81768 | -1.95902 | -1.02929 |
| C | -2.32078 | -0.75931 | -0.49606 |
| C | 1.85432 | -1.44059 | -0.46456 |
| C | 0.18705 | -3.39929 | -1.52511 |
| H | -3.38628 | -0.56763 | -0.47238 |
| C | 1.55146 | -3.59586 | -1.49047 |
| C | 2.39646 | -2.61609 | -0.94541 |

| | | | |
|---|---|---|---|
| H | -0.44256 | -4.18606 | -1.93023 |
| H | 1.97469 | -4.51889 | -1.87376 |
| H | 3.46947 | -2.77619 | -0.89124 |
| C | 2.71039 | -0.42649 | 0.17735 |
| O | 3.89928 | -0.36030 | 0.22350 |
| N | 0.73708 | 1.04117 | 0.34634 |
| O | 2.05614 | 0.92934 | 0.18740 |
| S | -2.84878 | -3.18580 | -1.68278 |
| C | -4.52485 | -2.54127 | -1.47128 |
| H | -5.17336 | -3.31611 | -1.88965 |
| H | -4.76600 | -2.40655 | -0.41324 |
| H | -4.66813 | -1.61545 | -2.03537 |
| O | 0.36035 | 2.10352 | 0.76094 |
| H | -1.87879 | 1.14752 | 0.37207 |
| O | 1.99215 | -0.70030 | 2.06799 |
| H | 2.72541 | -0.03899 | 2.84125 |
| H | 2.05033 | -1.63559 | 2.28908 |
| N | 3.46044 | 0.66267 | 3.54310 |
| C | 3.49233 | 2.02621 | 2.98797 |
| H | 2.47487 | 2.42425 | 2.95770 |
| H | 4.12494 | 2.68146 | 3.59631 |
| H | 3.88860 | 1.98246 | 1.97030 |
| C | 4.78890 | 0.03198 | 3.60502 |
| H | 5.48366 | 0.63334 | 4.20106 |
| H | 4.69280 | -0.96192 | 4.05012 |
| H | 5.17542 | -0.06795 | 2.58693 |
| H | 3.06224 | 0.69695 | 4.47980 |

**Single point energy: -1351.77000125 Hartrees**
**Gibbs free energy: -1351.517808 Hartrees**
**No. img. Freqs= 1**

| | | | |
|---|---|---|---|
| C | -1.07718 | -2.15415 | -1.13912 |
| C | 0.05475 | -1.68676 | -0.46956 |
| C | -0.02328 | -0.60657 | 0.45720 |
| C | -1.29468 | -0.07035 | 0.77319 |
| C | -2.45836 | -0.58405 | 0.08764 |
| C | -2.31495 | -1.59816 | -0.86959 |
| C | 1.13033 | -0.05831 | 1.04432 |
| C | -1.36107 | 0.96648 | 1.73615 |
| H | -3.17470 | -1.98325 | -1.40414 |
| C | -0.22054 | 1.46104 | 2.33168 |
| C | 1.04149 | 0.96098 | 1.96983 |
| H | -2.32177 | 1.38758 | 2.01767 |
| H | -0.29497 | 2.25097 | 3.07245 |
| H | 1.94711 | 1.36820 | 2.41074 |
| C | 2.45099 | -0.54934 | 0.60121 |
| O | 3.53001 | -0.36039 | 1.06110 |
| N | 1.25747 | -2.34480 | -0.66753 |
| O | 2.31221 | -2.00524 | 0.04681 |
| S | -3.99822 | 0.10645 | 0.48739 |
| C | -5.18665 | -0.81625 | -0.51440 |
| H | -6.15995 | -0.39610 | -0.24527 |
| H | -5.01446 | -0.66038 | -1.58248 |
| H | -5.17528 | -1.87947 | -0.25928 |
| O | 1.39779 | -3.27766 | -1.41044 |
| H | -0.98849 | -2.96658 | -1.85303 |
| O | 2.37513 | 0.13974 | -1.20761 |
| H | 2.07416 | 1.26548 | -1.33304 |
| H | 3.26989 | 0.00992 | -1.54451 |
| N | 1.65292 | 2.49220 | -1.56000 |
| C | 0.18514 | 2.50308 | -1.49315 |
| H | -0.12805 | 2.27345 | -0.47169 |
| H | -0.22398 | 3.47792 | -1.78365 |
| H | -0.20724 | 1.73516 | -2.16765 |
| C | 2.17230 | 2.91374 | -2.86972 |
| H | 1.82988 | 3.92159 | -3.13201 |
| H | 3.26536 | 2.89779 | -2.84567 |
| H | 1.82508 | 2.20892 | -3.63169 |
| H | 2.02247 | 3.11366 | -0.84327 |

**Single point energy: -1351.76777547 Hartrees**
**Gibbs free energy: -1351.513505 Hartrees**
**No. img. Freqs= 1**

| | | | |
|---|---|---|---|
| C | -0.66707 | -1.32847 | 1.45956 |
| C | 0.28523 | -0.35380 | 1.18633 |
| C | -0.01854 | 0.77958 | 0.37499 |
| C | -1.35722 | 0.93552 | -0.07709 |
| C | -2.33826 | -0.07600 | 0.24152 |
| C | -1.96225 | -1.19799 | 0.98161 |
| C | 0.93714 | 1.74636 | -0.01239 |
| C | -1.68583 | 2.08234 | -0.84289 |
| H | -2.67471 | -1.97741 | 1.22184 |
| C | -0.74216 | 3.02981 | -1.16536 |
| C | 0.58946 | 2.85290 | -0.76331 |
| H | -2.70456 | 2.22655 | -1.18979 |
| H | -1.01952 | 3.90319 | -1.74642 |
| H | 1.35210 | 3.57120 | -1.04888 |
| C | 2.35577 | 1.56394 | 0.28433 |
| O | 3.30053 | 2.24467 | 0.34861 |
| N | 1.55041 | -0.49966 | 1.78496 |
| O | 2.42209 | 0.42171 | 1.63417 |
| S | -3.96551 | 0.16284 | -0.33148 |
| C | -4.88655 | -1.25220 | 0.31407 |
| H | -5.91655 | -1.08118 | -0.01217 |
| H | -4.53088 | -2.19284 | -0.11467 |
| H | -4.86139 | -1.27666 | 1.40698 |
| O | 1.82806 | -1.45217 | 2.47167 |
| H | -0.40142 | -2.18342 | 2.07266 |
| O | 2.75657 | 0.15144 | -0.95919 |
| H | 2.28425 | -0.81124 | -1.01778 |

| | | | |
|---|---|---|---|
| H | 3.70934 | 0.00876 | -0.88861 |
| N | 1.67996 | -2.15259 | -1.23722 |
| C | 2.71709 | -2.96995 | -1.88154 |
| H | 3.56535 | -3.08176 | -1.19978 |
| H | 2.34450 | -3.96550 | -2.15669 |
| H | 3.06042 | -2.46136 | -2.78871 |
| C | 0.47954 | -2.00676 | -2.06862 |
| H | 0.07467 | -2.97956 | -2.37868 |
| H | -0.29140 | -1.46492 | -1.51307 |
| H | 0.73214 | -1.43041 | -2.96468 |
| H | 1.41721 | -2.60013 | -0.36152 |

**Single point energy: -1351.77011204 Hartrees**
**Gibbs free energy: -1351.518387 Hartrees**
**No. img. Freqs= 1**

| | | | |
|---|---|---|---|
| C | 1.08826 | -1.92703 | -0.53371 |
| C | 0.20558 | -0.84410 | -0.62396 |
| C | 0.62960 | 0.48561 | -0.33398 |
| C | 1.98703 | 0.71534 | -0.00867 |
| C | 2.88617 | -0.41394 | 0.06618 |
| C | 2.40671 | -1.71015 | -0.18927 |
| C | -0.27945 | 1.55573 | -0.34309 |
| C | 2.39425 | 2.04686 | 0.25041 |
| H | 3.06851 | -2.56579 | -0.13915 |
| C | 1.49432 | 3.08993 | 0.19800 |
| C | 0.14175 | 2.84575 | -0.08808 |
| H | 3.42982 | 2.26251 | 0.49641 |
| H | 1.82908 | 4.10408 | 0.39145 |
| H | -0.57731 | 3.65980 | -0.10350 |
| C | -1.70797 | 1.26997 | -0.57325 |
| O | -2.60713 | 2.02358 | -0.77122 |
| N | -1.07720 | -1.07926 | -1.06329 |
| O | -1.90271 | -0.06053 | -1.28588 |
| S | 4.53657 | -0.10413 | 0.48508 |
| C | 5.31649 | -1.73476 | 0.52555 |
| H | -0.35755 | -2.39814 | -0.17071 |
| H | -0.49700 | -3.59928 | -1.48276 |
| H | -0.27218 | -1.87300 | -1.87178 |
| C | -2.82151 | -2.65708 | -2.47774 |
| H | -2.63199 | -3.69419 | -2.77787 |
| H | -3.90006 | -2.49323 | -2.40411 |
| H | -2.41294 | -1.98371 | -3.23755 |
| H | -2.62360 | -2.92779 | -0.46323 |

**Single point energy: -1351.77042006Hartrees**
**Gibbs free energy: -1351.519086 Hartrees**
**No. img. Freqs= 1**

| | | | |
|---|---|---|---|
| C | 1.15606 | -1.98738 | -0.41952 |
| C | 0.27217 | -0.92467 | -0.64255 |
| C | 0.66363 | 0.42714 | -0.41657 |
| C | 1.99653 | 0.69840 | -0.03014 |
| C | 2.90528 | -0.40992 | 0.15951 |
| C | 2.45151 | -1.72812 | -0.02179 |
| C | -0.26080 | 1.47753 | -0.53544 |
| C | 2.36707 | 2.04921 | 0.17881 |
| H | 3.11719 | -2.56876 | 0.12936 |
| C | 1.45397 | 3.07067 | 0.02281 |
| C | 0.12348 | 2.78696 | -0.32390 |
| H | 3.38291 | 2.29751 | 0.47180 |
| H | 1.76059 | 4.09935 | 0.18346 |
| H | -0.60737 | 3.58491 | -0.41993 |
| C | -1.66599 | 1.14534 | -0.83205 |
| O | -2.57569 | 1.86796 | -1.09695 |
| N | -0.98062 | -1.20483 | -1.13424 |
| O | -1.79362 | -0.20716 | -1.48569 |
| S | 4.53430 | -0.04633 | 0.61529 |
| C | 5.33170 | -1.65777 | 0.80686 |
| H | 6.35733 | -1.42527 | 1.10704 |
| H | 4.85332 | -2.24190 | 1.59768 |
| H | 5.35240 | -2.20619 | -0.13863 |
| O | -1.39758 | -2.30851 | -1.35891 |
| H | 0.82788 | -3.00921 | -0.58008 |
| O | -2.05277 | 0.22173 | 0.94889 |
| H | -3.28855 | 0.01125 | 0.90833 |
| H | -1.84618 | 0.82737 | 1.66804 |
| N | -4.48810 | -0.26127 | 0.82160 |
| C | -5.06276 | -0.33900 | 2.17378 |
| H | -4.92068 | 0.62006 | 2.67884 |
| H | -6.13131 | -0.57630 | 2.13615 |
| H | -4.53965 | -1.11895 | 2.73469 |
| C | -4.63922 | -1.50596 | 0.05071 |
| H | -5.69309 | -1.79484 | -0.02612 |
| H | -4.22710 | -1.35903 | -0.95015 |
| H | -4.08242 | -2.30164 | 0.55396 |
| H | -4.93248 | 0.50068 | 0.31172 |

**TS structures for the dimethylamine catalysed water addition to C-7 of the naphtho oxazinium cation A to yield intermediate B (TS-AB analogue).**

**Single point energy: -1351.7691593 Hartrees**
**Gibbs free energy: -1351.513393 Hartrees**
**No. img. Freqs= 1**

| | | | |
|---|---|---|---|
| C | -0.32850 | 1.08727 | -1.69339 |
| C | 0.56224 | -0.02535 | -1.50923 |
| C | 0.32798 | -0.99255 | -0.48065 |
| C | -0.86764 | -0.92670 | 0.26398 |
| C | -1.87886 | 0.06959 | -0.11553 |
| C | -1.60273 | 1.01771 | -1.07120 |
| C | 1.28427 | -1.97234 | -0.19074 |
| C | -1.03310 | -1.83018 | 1.32330 |
| H | -2.33600 | 1.75919 | -1.36359 |
| C | -0.06184 | -2.78005 | 1.62641 |
| C | 1.09699 | -2.86582 | 0.86296 |
| H | -1.93185 | -1.79830 | 1.93317 |
| H | -0.21658 | -3.46153 | 2.45661 |
| H | 1.85539 | -3.61282 | 1.07594 |
| C | 2.49197 | -2.03836 | -1.00562 |
| O | 3.40220 | -2.80540 | -0.93087 |
| N | 1.68392 | -0.05326 | -2.23218 |
| O | 2.60669 | -1.07851 | -2.03439 |
| S | -3.42001 | -0.05537 | 0.68375 |
| C | -4.39834 | 1.28131 | -0.03684 |
| H | -5.36951 | 1.22005 | 0.46229 |
| H | -3.95357 | 2.25840 | 0.17059 |
| H | -4.53933 | 1.13034 | -1.11085 |
| O | 2.03028 | 0.72853 | -3.08190 |
| H | -0.22475 | 1.66954 | -2.60215 |
| O | 0.73901 | 2.40338 | -0.69844 |
| H | 0.59135 | 2.21624 | 0.34825 |
| H | 0.43789 | 3.29881 | -0.89843 |
| N | 0.48708 | 1.95078 | 1.81132 |
| C | 0.46699 | 3.25689 | 2.48201 |
| H | -0.40369 | 3.82728 | 2.14373 |
| H | 0.42718 | 3.15903 | 3.57511 |
| H | 1.37274 | 3.81114 | 2.21375 |
| C | 1.60739 | 1.11191 | 2.25376 |
| H | 1.63840 | 1.01137 | 3.34713 |
| H | 1.51815 | 0.11543 | 1.81147 |
| H | 2.54761 | 1.56063 | 1.91629 |
| H | -0.38357 | 1.46648 | 2.02004 |

**Single point energy: -1351.76817622 Hartrees**
**Gibbs free energy: -1351.516143 Hartrees**
**No. img. Freqs= 1**

| | | | |
|---|---|---|---|
| C | 0.06293 | -1.34150 | -0.95952 |
| C | -0.34017 | 0.02694 | -0.80194 |
| C | 0.52064 | 0.99443 | -0.18815 |
| C | 1.82566 | 0.61119 | 0.18249 |
| C | 2.27785 | -0.74987 | -0.13786 |
| C | 1.41623 | -1.66139 | -0.70903 |
| C | 0.07363 | 2.30001 | 0.04622 |
| C | 2.63499 | 1.56660 | 0.81440 |
| H | 1.74162 | -2.66046 | -0.96949 |
| C | 2.17939 | 2.85919 | 1.05569 |
| C | 0.89950 | 3.23725 | 0.66639 |
| H | 3.64219 | 1.30874 | 1.12853 |
| H | 2.83211 | 3.57538 | 1.54409 |
| H | 0.53697 | 4.24637 | 0.83707 |
| C | -1.27116 | 2.67084 | -0.37859 |
| O | -1.82552 | 3.72209 | -0.28267 |
| N | -1.58088 | 0.35158 | -1.16459 |
| O | -2.03544 | 1.65421 | -0.99735 |
| S | 3.94498 | -1.10790 | 0.19563 |
| C | 4.13017 | -2.84552 | -0.26398 |
| H | 5.17208 | -3.08592 | -0.03378 |
| H | 3.47435 | -3.48482 | 0.33352 |
| H | 3.95894 | -2.99249 | -1.33378 |
| O | -2.42111 | -0.38221 | -1.63186 |
| H | -0.50965 | -1.96960 | -1.63034 |
| O | -0.93571 | -2.22100 | 0.54802 |
| H | -1.99655 | -2.08095 | 0.80791 |
| H | -0.41060 | -2.06057 | 1.34134 |
| N | -3.36272 | -1.98891 | 1.22111 |
| C | -4.25629 | -2.62482 | 0.24296 |
| H | -3.93164 | -3.65579 | 0.07595 |
| H | -5.29862 | -2.62714 | 0.58725 |
| H | -4.19697 | -2.07976 | -0.70325 |
| C | -3.74609 | -0.60484 | 1.52873 |
| H | -4.74070 | -0.55082 | 1.98995 |
| H | -3.01345 | -0.16667 | 2.21381 |
| H | -3.76561 | -0.02038 | 0.60423 |
| H | -3.40645 | -2.52725 | 2.08465 |

**Single point energy: -1351.7689745 Hartrees**
**Gibbs free energy: -1351.516161 Hartrees**
**No. img. Freqs= 1**

| | | | |
|---|---|---|---|
| C | 0.71359 | 0.76690 | -0.70473 |
| C | 0.19492 | -0.56490 | -0.56599 |
| C | -1.13540 | -0.79956 | -0.09016 |
| C | -1.98342 | 0.30101 | 0.14730 |
| C | -1.49461 | 1.64751 | -0.17641 |
| C | -0.19711 | 1.84221 | -0.60112 |
| C | -1.58489 | -2.10245 | 0.15216 |
| C | -3.26158 | 0.04505 | 0.66496 |
| H | 0.16790 | 2.82652 | -0.86766 |

| | | | |
|---|---|---|---|
| C | -3.69606 | -1.25274 | 0.91723 |
| C | -2.86440 | -2.33547 | 0.65470 |
| H | -3.93712 | 0.86640 | 0.88582 |
| H | -4.69069 | -1.41648 | 1.31903 |
| H | -3.19343 | -3.35385 | 0.83812 |
| C | -0.69246 | -3.21985 | -0.13257 |
| O | -0.89624 | -4.38917 | -0.01798 |
| N | 1.03176 | -1.57938 | -0.77615 |
| O | 0.59064 | -2.88789 | -0.62442 |
| S | -2.65329 | 2.93591 | -0.05158 |
| C | -1.71534 | 4.42302 | -0.46658 |
| H | -2.43227 | 5.24301 | -0.36791 |
| H | -0.89341 | 4.58176 | 0.23716 |
| H | -1.35446 | 4.38774 | -1.49814 |
| O | 2.19756 | -1.51174 | -1.09553 |
| H | 1.62164 | 0.90387 | -1.28172 |
| O | 1.80614 | 0.75543 | 0.98474 |
| H | 2.87383 | 0.59285 | 0.80529 |
| H | 1.68350 | 1.61029 | 1.41480 |
| N | 4.26557 | 0.36250 | 0.54085 |
| C | 5.06616 | 0.92003 | 1.63712 |
| H | 4.79687 | 0.42116 | 2.57247 |
| H | 6.14304 | 0.79826 | 1.46177 |
| H | 4.84677 | 1.98899 | 1.73412 |
| C | 4.60078 | 0.94563 | -0.76264 |
| H | 5.66695 | 0.83488 | -1.00028 |
| H | 4.01100 | 0.45531 | -1.54251 |
| H | 4.35668 | 2.01390 | -0.75151 |
| H | 4.41873 | -0.64240 | 0.49924 |

**Single point energy: -1351.7674298 Hartrees**
**Gibbs free energy: -1351.511487 Hartrees**
**No. img. Freqs= 1**

| | | | |
|---|---|---|---|
| C | 0.44614 | -0.75761 | -1.34268 |
| C | -0.91140 | -0.80768 | -0.87051 |
| C | -1.46767 | 0.24627 | -0.07612 |
| C | -0.70093 | 1.40404 | 0.16269 |
| C | 0.62538 | 1.50847 | -0.46164 |
| C | 1.14447 | 0.46518 | -1.19434 |
| C | -2.75473 | 0.12867 | 0.45993 |
| C | -1.26083 | 2.40786 | 0.96532 |
| H | 2.11043 | 0.54492 | -1.67693 |
| C | -2.53669 | 2.27766 | 1.50698 |
| C | -3.29435 | 1.14031 | 1.25371 |
| H | -0.70072 | 3.31316 | 1.18166 |
| H | -2.94082 | 3.07277 | 2.12511 |
| H | -4.29459 | 1.02907 | 1.66131 |
| C | -3.53328 | -1.06901 | 0.16840 |
| O | -4.64150 | -1.34348 | 0.51252 |
| N | -1.59982 | -1.92435 | -1.09723 |
| O | -2.90606 | -2.04263 | -0.64162 |
| S | 1.44297 | 3.03210 | -0.26820 |
| C | 2.97559 | 2.83962 | -1.20505 |
| H | 3.50123 | 3.79089 | -1.08350 |
| H | 3.59367 | 2.03675 | -0.79571 |
| H | 2.76826 | 2.67959 | -2.26666 |
| O | -1.21006 | -2.91684 | -1.67217 |
| H | 0.72455 | -1.41799 | -2.15592 |
| O | 1.22492 | -2.01743 | -0.02825 |
| H | 2.26758 | -1.88636 | 0.26813 |
| H | 1.12966 | -2.92045 | -0.35827 |
| N | 3.64326 | -1.77947 | 0.70349 |
| C | 4.20410 | -0.47880 | 0.32575 |
| H | 3.63145 | 0.31527 | 0.81430 |
| H | 5.25942 | -0.38706 | 0.61499 |
| H | 4.12754 | -0.35738 | -0.76077 |
| C | 4.40100 | -2.90790 | 0.14879 |
| H | 5.45734 | -2.87832 | 0.44567 |
| H | 3.95927 | -3.84672 | 0.49545 |
| H | 4.34280 | -2.87586 | -0.94442 |
| H | 3.64596 | -1.85164 | 1.71855 |

**Single point energy: -1351.76883651 Hartrees**
**Gibbs free energy: -1351.515218 Hartrees**
**No. img. Freqs= 1**

| | | | |
|---|---|---|---|
| C | -0.32655 | 1.07906 | -1.64711 |
| C | 0.81227 | 0.29081 | -1.26320 |
| C | 0.67919 | -0.81592 | -0.36272 |
| C | -0.60966 | -1.21583 | 0.04922 |
| C | -1.77540 | -0.52250 | -0.51548 |
| C | -1.61315 | 0.56507 | -1.34056 |
| C | 1.81055 | -1.48548 | 0.11754 |
| C | -0.70805 | -2.26692 | 0.97234 |
| H | -2.46315 | 1.07168 | -1.78060 |
| C | 0.42351 | -2.91622 | 1.45789 |
| C | 1.68930 | -2.53569 | 1.02666 |
| H | -1.68190 | -2.59147 | 1.32828 |
| H | 0.31371 | -3.72645 | 2.17145 |
| H | 2.58039 | -3.04065 | 1.38675 |
| C | 3.12704 | -1.06898 | -0.35251 |
| O | 4.19635 | -1.51332 | -0.06672 |
| N | 2.01538 | 0.68860 | -1.68535 |
| O | 3.15420 | -0.00624 | -1.28020 |
| S | -3.33442 | -1.19068 | -0.12159 |
| C | -4.50711 | -0.07216 | -0.92032 |
| H | -5.49380 | -0.46248 | -0.65505 |
| H | -4.41066 | 0.94609 | -0.53300 |
| H | -4.39630 | -0.09431 | -2.00808 |
| O | 2.27070 | 1.61965 | -2.40611 |
| H | -0.21801 | 1.73508 | -2.50295 |

| | | | |
|---|---|---|---|
| O | 0.00364 | 2.61282 | -0.45486 |
| H | -0.15225 | 2.28699 | 0.55457 |
| H | -0.61565 | 3.32628 | -0.65477 |
| N | -0.27361 | 1.83110 | 1.96753 |
| C | -1.66941 | 1.92520 | 2.40660 |
| H | -2.29323 | 1.27656 | 1.78384 |
| H | -1.79335 | 1.63344 | 3.45801 |
| H | -2.01431 | 2.95826 | 2.29006 |
| C | 0.63553 | 2.64122 | 2.78783 |
| H | 0.57568 | 2.37748 | 3.85226 |
| H | 1.66281 | 2.49913 | 2.43974 |
| H | 0.37494 | 3.69869 | 2.67376 |
| H | 0.01894 | 0.85818 | 2.02725 |

**Single point energy: -1351.76880385 Hartrees**
**Gibbs free energy: -1351.513997 Hartrees**
**No. img. Freqs= 1**

| | | | |
|---|---|---|---|
| C | -0.47147 | 1.10037 | -1.63103 |
| C | 0.67731 | 0.35095 | -1.20011 |
| C | 0.54569 | -0.78799 | -0.34028 |
| C | -0.74338 | -1.23015 | 0.02456 |
| C | -1.91097 | -0.53972 | -0.54045 |
| C | -1.75176 | 0.54926 | -1.36400 |
| C | 1.67958 | -1.45131 | 0.14300 |
| C | -0.83983 | -2.32279 | 0.89873 |
| H | -2.60243 | 1.04040 | -1.82038 |
| C | 0.29354 | -2.96714 | 1.38566 |
| C | 1.56059 | -2.53983 | 1.00560 |
| H | -1.81333 | -2.68797 | 1.21312 |
| H | 0.18433 | -3.81017 | 2.06010 |
| H | 2.45396 | -3.03891 | 1.36845 |
| C | 2.99833 | -0.98321 | -0.26798 |
| O | 4.06910 | -1.42023 | 0.02347 |
| N | 1.88268 | 0.80379 | -1.55702 |
| O | 3.02792 | 0.13324 | -1.13079 |
| S | -3.46908 | -1.20773 | -0.14234 |
| C | -4.64595 | -0.09393 | -0.94212 |
| H | -5.63094 | -0.48340 | -0.66943 |
| H | -4.54760 | 0.92628 | -0.56089 |
| H | -4.54239 | -0.12198 | -2.03016 |
| O | 2.13292 | 1.76865 | -2.23310 |
| H | -0.35063 | 1.75658 | -2.48525 |
| O | -0.26276 | 2.66489 | -0.45152 |
| H | -0.28624 | 2.31100 | 0.55722 |
| H | -1.00741 | 3.26369 | -0.58944 |
| N | -0.13784 | 1.80942 | 1.95821 |
| C | -0.90260 | 2.65400 | 2.88270 |
| H | -1.96887 | 2.57764 | 2.64877 |
| H | -0.74522 | 2.36594 | 3.93084 |
| H | -0.59074 | 3.69627 | 2.75699 |
| C | 1.30051 | 1.81069 | 2.24779 |
| H | 1.51996 | 1.46872 | 3.26793 |
| H | 1.81488 | 1.15859 | 1.53526 |
| H | 1.68972 | 2.82651 | 2.12491 |
| H | -0.48308 | 0.85479 | 2.02984 |

**Single point energy: -1351.76975183 Hartrees**
**Gibbs free energy: -1351.513885 Hartrees**
**No. img. Freqs= 1**

| | | | |
|---|---|---|---|
| C | 0.46472 | -1.16528 | -1.52392 |
| C | -0.43658 | -0.07746 | -1.25547 |
| C | -0.07731 | 0.99222 | -0.37221 |
| C | 1.21077 | 1.00602 | 0.19847 |
| C | 2.16726 | -0.03967 | -0.19367 |
| C | 1.79220 | -1.05166 | -1.04596 |
| C | -1.00392 | 1.98849 | -0.04759 |
| C | 1.51288 | 2.02578 | 1.11149 |
| H | 2.49454 | -1.80933 | -1.36903 |
| C | 0.58225 | 3.00842 | 1.43787 |
| C | -0.67999 | 3.00031 | 0.85574 |
| H | 2.48907 | 2.06095 | 1.58673 |
| H | 0.84858 | 3.78450 | 2.14805 |
| H | -1.41386 | 3.76370 | 1.09525 |
| C | -2.32914 | 1.94319 | -0.65383 |
| O | -3.24415 | 2.69431 | -0.51099 |
| N | -1.66253 | -0.14947 | -1.77289 |
| O | -2.57701 | 0.87021 | -1.54127 |
| S | 3.78048 | 0.11750 | 0.43818 |
| C | 4.64213 | -1.34555 | -0.17967 |
| H | 5.64889 | -1.27747 | 0.24178 |
| H | 4.16508 | -2.26259 | 0.17719 |
| H | 4.71209 | -1.33374 | -1.27091 |
| O | -2.11830 | -1.04561 | -2.44743 |
| H | 0.28250 | -1.76329 | -2.40959 |
| O | -0.29199 | -2.63413 | -0.44167 |
| H | -0.69366 | -2.38517 | 0.52687 |
| H | -0.98545 | -3.09191 | -0.93458 |
| N | -1.30812 | -2.15910 | 1.85331 |
| C | -2.40011 | -3.13505 | 1.96766 |
| H | -1.99764 | -4.14604 | 1.85550 |
| H | -2.91972 | -3.06022 | 2.93174 |
| H | -3.12591 | -2.95605 | 1.16661 |
| C | -1.77298 | -0.77750 | 2.01327 |
| H | -2.38529 | -0.65081 | 2.91614 |
| H | -0.91165 | -0.10584 | 2.07404 |
| H | -2.37837 | -0.49971 | 1.14188 |
| H | -0.61854 | -2.35895 | 2.57407 |

**Single point energy: -1351.77136936 Hartrees**
**Gibbs free energy: -1351.514762 Hartrees**
**No. img. Freqs= 1**

| | | | |
|---|---|---|---|
| C | -0.54080 | 1.15760 | -1.51098 |
| C | 0.46063 | 0.19355 | -1.14498 |
| C | 0.15257 | -0.92973 | -0.30946 |
| C | -1.17148 | -1.11179 | 0.13643 |
| C | -2.19902 | -0.15652 | -0.30121 |
| C | -1.87613 | 0.89613 | -1.12691 |
| C | 1.15312 | -1.83607 | 0.06018 |
| C | -1.43617 | -2.20245 | 0.97652 |
| H | -2.63394 | 1.57480 | -1.49732 |
| C | -0.43393 | -3.09371 | 1.34912 |
| C | 0.86613 | -2.91995 | 0.88927 |
| H | -2.44040 | -2.37214 | 1.35414 |
| H | -0.67324 | -3.92978 | 1.99825 |
| H | 1.65705 | -3.61112 | 1.16445 |
| C | 2.51038 | -1.62907 | -0.43185 |
| O | 3.48346 | -2.29209 | -0.24494 |
| N | 1.71225 | 0.42037 | -1.54559 |
| O | 2.71752 | -0.48914 | -1.24230 |
| S | -3.82135 | -0.46896 | 0.23952 |
| C | -4.78658 | 0.89523 | -0.44683 |
| H | -5.80309 | 0.73360 | -0.07697 |
| H | -4.42319 | 1.86015 | -0.08251 |
| H | -4.79716 | 0.86091 | -1.53966 |
| O | 2.11546 | 1.37863 | -2.16482 |
| H | -0.35914 | 1.77048 | -2.38688 |
| O | -0.05566 | 2.73916 | -0.40121 |
| H | 0.26357 | 2.47280 | 0.58203 |
| H | 0.65732 | 3.24446 | -0.81346 |
| N | 0.69592 | 2.15789 | 1.97794 |
| C | 2.01353 | 1.51480 | 1.98702 |
| H | 2.72252 | 2.11897 | 1.41295 |
| H | 2.40215 | 1.38002 | 3.00548 |
| H | 1.93337 | 0.52841 | 1.52051 |
| C | -0.31491 | 1.39255 | 2.71671 |
| H | -0.05364 | 1.28251 | 3.77794 |
| H | -1.28324 | 1.89608 | 2.63814 |
| H | -0.40553 | 0.39204 | 2.28110 |
| H | 0.78364 | 3.07127 | 2.41925 |

**Single point energy: -1351.76826208 Hartrees**
**Gibbs free energy: -1351.516001 Hartrees**
**No. img. Freqs= 1**

| | | | |
|---|---|---|---|
| C | -0.63707 | -0.91113 | -0.62944 |
| C | -0.29108 | 0.47869 | -0.53138 |
| C | 1.01231 | 0.89416 | -0.10919 |
| C | 2.00357 | -0.08211 | 0.11807 |
| C | 1.67958 | -1.48931 | -0.15083 |
| C | 0.40952 | -1.85769 | -0.54029 |
| C | 1.29976 | 2.25028 | 0.08277 |
| C | 3.26003 | 0.35013 | 0.56649 |
| H | 0.16839 | -2.88802 | -0.77168 |
| C | 3.53432 | 1.69951 | 0.76729 |
| C | 2.55913 | 2.65898 | 0.51998 |
| H | 4.04729 | -0.37048 | 0.76836 |
| H | 4.51692 | 2.00143 | 1.11518 |
| H | 2.76341 | 3.71563 | 0.66371 |
| C | 0.25928 | 3.23557 | -0.18602 |
| O | 0.31417 | 4.42404 | -0.10528 |
| N | -1.25899 | 1.37120 | -0.73532 |
| O | -0.99058 | 2.72834 | -0.60919 |
| S | 2.98758 | -2.62364 | 0.00293 |
| C | 2.23236 | -4.22379 | -0.36150 |
| H | 3.03840 | -4.95195 | -0.23414 |
| H | 1.42978 | -4.45335 | 0.34500 |
| H | 1.87483 | -4.26647 | -1.39383 |
| O | -2.41175 | 1.14636 | -1.02562 |
| H | -1.53731 | -1.17255 | -1.17427 |
| O | -1.65331 | -1.02344 | 1.09206 |
| H | -2.72390 | -1.20837 | 0.98021 |
| H | -1.26439 | -1.74545 | 1.60046 |
| N | -4.14393 | -1.37920 | 0.78807 |
| C | -4.39444 | -1.79424 | -0.59753 |
| H | -3.87422 | -2.73693 | -0.79455 |
| H | -5.46521 | -1.93097 | -0.79835 |
| H | -4.00800 | -1.02657 | -1.27590 |
| C | -4.84632 | -0.13963 | 1.14732 |
| H | -5.93198 | -0.23232 | 1.01205 |
| H | -4.63442 | 0.10526 | 2.19199 |
| H | -4.47937 | 0.67531 | 0.51651 |
| H | -4.46274 | -2.11965 | 1.40936 |

**Single point energy: -1351.76664094 Hartrees**
**Gibbs free energy: -1351.511794 Hartrees**
**No. img. Freqs= 1**

| | | | |
|---|---|---|---|
| C | 0.82373 | -0.45976 | -0.75372 |
| C | -0.54280 | -0.85966 | -0.56842 |
| C | -1.51923 | 0.05505 | -0.05765 |
| C | -1.15662 | 1.40229 | 0.14835 |
| C | 0.19067 | 1.83614 | -0.24459 |
| C | 1.11893 | 0.92443 | -0.69534 |
| C | -2.81566 | -0.37910 | 0.24160 |
| C | -2.11747 | 2.26509 | 0.69559 |
| H | 2.11173 | 1.22989 | -1.00324 |
| C | -3.39783 | 1.81983 | 1.01050 |
| C | -3.75861 | 0.49763 | 0.77626 |
| H | -1.87224 | 3.30587 | 0.88803 |
| H | -4.11763 | 2.51261 | 1.43423 |

| | | | |
|---|---|---|---|
| H | -4.75808 | 0.13944 | 1.00397 |
| C | -3.16997 | -1.76865 | -0.02245 |
| O | -4.21482 | -2.31872 | 0.14389 |
| N | -0.84944 | -2.14088 | -0.77925 |
| O | -2.15010 | -2.58419 | -0.56250 |
| S | 0.50719 | 3.54435 | -0.15656 |
| C | 2.22219 | 3.70071 | -0.70104 |
| H | 2.43572 | 4.77236 | -0.65681 |
| H | 2.90281 | 3.17369 | -0.02635 |
| H | 2.34073 | 3.35674 | -1.73227 |
| O | -0.11125 | -3.02045 | -1.15057 |
| H | 1.45782 | -1.10099 | -1.35612 |
| O | 1.58252 | -1.29029 | 0.88186 |
| H | 2.61915 | -1.49614 | 0.67093 |
| H | 1.54346 | -0.61896 | 1.57528 |
| N | 4.02930 | -1.72193 | 0.31868 |
| C | 4.76876 | -2.15333 | 1.51077 |
| H | 4.36466 | -3.10709 | 1.86096 |
| H | 5.84214 | -2.26881 | 1.30961 |
| H | 4.63925 | -1.40808 | 2.30302 |
| C | 4.54778 | -0.47403 | -0.25205 |
| H | 5.61559 | -0.54214 | -0.49866 |
| H | 3.98921 | -0.22928 | -1.16109 |
| H | 4.40936 | 0.33596 | 0.47322 |
| H | 4.08816 | -2.45539 | -0.38405 |

**Single point energy: -1351.76877077 Hartrees**
**Gibbs free energy: -1351.515811 Hartrees**
**No. img. Freqs= 1**

| | | | |
|---|---|---|---|
| C | 0.55784 | 1.00926 | -1.03920 |
| C | 0.20730 | -0.35317 | -0.75155 |
| C | -1.02033 | -0.68597 | -0.09067 |
| C | -1.93973 | 0.34058 | 0.20538 |
| C | -1.62670 | 1.71077 | -0.22296 |
| C | -0.43086 | 1.99818 | -0.84540 |
| C | -1.30410 | -2.01061 | 0.26088 |
| C | -3.12002 | -0.00947 | 0.87757 |
| H | -0.19888 | 2.99837 | -1.18875 |
| C | -3.39030 | -1.32755 | 1.23313 |
| C | -2.48755 | -2.33771 | 0.92169 |
| H | -3.85086 | 0.75232 | 1.13388 |
| H | -4.31315 | -1.56514 | 1.75212 |
| H | -2.68929 | -3.37187 | 1.18391 |
| C | -0.34458 | -3.05269 | -0.08257 |
| O | -0.40782 | -4.22624 | 0.11751 |
| N | 1.10120 | -1.29839 | -1.04273 |
| O | 0.82846 | -2.62886 | -0.75036 |
| S | -2.85480 | 2.90593 | 0.06084 |
| C | -2.13329 | 4.44329 | -0.55539 |
| H | -2.88805 | 5.20850 | -0.35233 |
| H | -1.21571 | 4.69340 | -0.01555 |
| H | -1.95939 | 4.39220 | -1.63381 |
| O | 2.19388 | -1.14238 | -1.54010 |
| H | 1.35316 | 1.19276 | -1.75090 |
| O | 1.92773 | 1.38075 | 0.39448 |
| H | 2.85047 | 0.79110 | 0.44209 |
| H | 1.48754 | 1.34504 | 1.25238 |
| N | 4.12251 | 0.12487 | 0.48085 |
| C | 4.67849 | 0.20147 | 1.83630 |
| H | 4.01320 | -0.31772 | 2.53283 |
| H | 5.67807 | -0.24865 | 1.89530 |
| H | 4.74813 | 1.25253 | 2.13604 |
| C | 4.98565 | 0.75628 | -0.52437 |
| H | 5.99185 | 0.31737 | -0.53289 |
| H | 4.53583 | 0.63745 | -1.51416 |
| H | 5.06842 | 1.82544 | -0.30285 |
| H | 3.99613 | -0.85282 | 0.23039 |

**Single point energy: -1351.76973139 Hartrees**
**Gibbs free energy: -1351.515857 Hartrees**
**No. img. Freqs= 1**

| | | | |
|---|---|---|---|
| C | -0.28861 | 1.06479 | -1.65152 |
| C | 0.85764 | 0.38183 | -1.11256 |
| C | 0.71051 | -0.75576 | -0.25216 |
| C | -0.57876 | -1.27242 | -0.01132 |
| C | -1.71997 | -0.67294 | -0.71817 |
| C | -1.55031 | 0.43577 | -1.51407 |
| C | 1.82792 | -1.33377 | 0.36072 |
| C | -0.69860 | -2.34587 | 0.88238 |
| H | -2.37997 | 0.87748 | -2.05111 |
| C | 0.41634 | -2.90539 | 1.50096 |
| C | 1.68779 | -2.40999 | 1.23647 |
| H | -1.67633 | -2.76112 | 1.10978 |
| H | 0.28914 | -3.73679 | 2.18677 |
| H | 2.56818 | -2.84556 | 1.69920 |
| C | 3.15102 | -0.79560 | 0.06620 |
| O | 4.21300 | -1.16522 | 0.46234 |
| N | 2.05856 | 0.91633 | -1.33379 |
| O | 3.19638 | 0.31381 | -0.80739 |
| S | -3.25135 | -1.47575 | -0.53022 |
| C | -4.39494 | -0.46518 | -1.49686 |
| H | -5.36973 | -0.94541 | -1.37368 |
| H | -4.44584 | 0.55513 | -1.10674 |
| H | -4.12537 | -0.47199 | -2.55689 |
| O | 2.30628 | 1.92674 | -1.95154 |
| H | -0.13295 | 1.73486 | -2.48908 |
| O | -0.44030 | 2.63274 | -0.45957 |
| H | -0.42156 | 2.28653 | 0.55310 |
| H | 0.27233 | 3.27212 | -0.59054 |
| N | -0.49910 | 1.80827 | 1.96358 |

| | | | |
|---|---|---|---|
| C | -1.91494 | 1.67819 | 2.32550 |
| H | -2.38816 | 0.92878 | 1.68350 |
| H | -2.04712 | 1.38354 | 3.37522 |
| H | -2.41547 | 2.63847 | 2.16368 |
| C | 0.22011 | 2.75948 | 2.81944 |
| H | 0.14881 | 2.49468 | 3.88282 |
| H | 1.27478 | 2.78588 | 2.52888 |
| H | -0.20510 | 3.75895 | 2.67941 |
| H | -0.05769 | 0.89535 | 2.05096 |

**Single point energy: -1351.77045221 Hartrees**
**Gibbs free energy: -1351.514628 Hartrees**
**No. img. Freqs= 1**

| | | | |
|---|---|---|---|
| C | -0.45763 | 1.09560 | -1.62504 |
| C | 0.68883 | 0.34754 | -1.18220 |
| C | 0.55303 | -0.80399 | -0.33910 |
| C | -0.73729 | -1.24358 | 0.02003 |
| C | -1.90183 | -0.54193 | -0.54052 |
| C | -1.73781 | 0.55120 | -1.35904 |
| C | 1.68547 | -1.47226 | 0.13968 |
| C | -0.83786 | -2.34160 | 0.88644 |
| H | -2.58521 | 1.05237 | -1.80925 |
| C | 0.29386 | -2.99277 | 1.36947 |
| C | 1.56269 | -2.56755 | 0.99374 |
| H | -1.81247 | -2.70564 | 1.19861 |
| H | 0.18134 | -3.84005 | 2.03804 |
| H | 2.45405 | -3.07269 | 1.35316 |
| C | 3.00545 | -1.00581 | -0.26946 |
| O | 4.07504 | -1.45731 | 0.00137 |
| N | 1.89503 | 0.81991 | -1.49874 |
| O | 3.03758 | 0.13453 | -1.10432 |
| S | -3.46184 | -1.20276 | -0.14602 |
| C | -4.63339 | -0.07804 | -0.93843 |
| H | -5.62004 | -0.46158 | -0.66330 |
| H | -4.52483 | 0.94004 | -0.55446 |
| H | -4.53289 | -0.10418 | -2.02680 |
| O | 2.14305 | 1.83475 | -2.10901 |
| H | -0.33688 | 1.74778 | -2.48231 |
| O | -0.41639 | 2.69445 | -0.45643 |
| H | -0.27725 | 2.34515 | 0.54530 |
| H | 0.30383 | 3.29739 | -0.68350 |
| N | -0.11196 | 1.83867 | 1.93565 |
| C | -0.87516 | 2.69882 | 2.84794 |
| H | -1.93644 | 2.65180 | 2.58719 |
| H | -0.74811 | 2.40155 | 3.89759 |
| H | -0.53495 | 3.73335 | 2.73322 |
| C | 1.31929 | 1.80412 | 2.25142 |
| H | 1.51200 | 1.46381 | 3.27744 |
| H | 1.83069 | 1.13312 | 1.55450 |
| H | 1.73768 | 2.80934 | 2.13325 |
| H | -0.48284 | 0.89260 | 1.99682 |

**Single point energy: -1351.76824194 Hartrees**
**Gibbs free energy: -1351.515487 Hartrees**
**No. img. Freqs= 1**

| | | | |
|---|---|---|---|
| C | 0.70491 | 0.76726 | -0.67025 |
| C | 0.20235 | -0.56770 | -0.50153 |
| C | -1.14136 | -0.81067 | -0.06326 |
| C | -2.00974 | 0.28298 | 0.12748 |
| C | -1.51824 | 1.63382 | -0.17864 |
| C | -0.21768 | 1.83337 | -0.58569 |
| C | -1.58862 | -2.11609 | 0.17145 |
| C | -3.30979 | 0.01589 | 0.58057 |
| H | 0.14901 | 2.81960 | -0.84014 |
| C | -3.74430 | -1.28437 | 0.82043 |
| C | -2.88892 | -2.35982 | 0.61166 |
| H | -4.00758 | 0.82988 | 0.75311 |
| H | -4.75808 | -1.45570 | 1.16737 |
| H | -3.21600 | -3.38078 | 0.78403 |
| C | -0.67489 | -3.22791 | -0.05963 |
| O | -0.86762 | -4.39675 | 0.07374 |
| N | 1.05209 | -1.57996 | -0.68115 |
| O | 0.62187 | -2.88987 | -0.51224 |
| S | -2.67490 | 2.92466 | -0.04639 |
| C | -1.72614 | 4.41360 | -0.43050 |
| H | 4.15823 | -2.57988 | 2.18226 |
| H | 5.57713 | -1.51940 | 1.95899 |
| H | 3.99906 | -0.82631 | 2.41635 |
| C | 4.56322 | -0.17523 | -0.15727 |
| H | 5.65446 | -0.06994 | -0.09533 |
| H | 4.26000 | -0.11060 | -1.20688 |
| H | 4.10406 | 0.64929 | 0.39854 |
| H | 4.50918 | -2.20809 | -0.12248 |

**Single point energy: -1351.76703073 Hartrees**
**Gibbs free energy: -1351.514463 Hartrees**
**No. img. Freqs= 1**

| | | | |
|---|---|---|---|
| C | 1.01240 | -0.07341 | -0.82505 |
| C | -0.27381 | -0.65620 | -0.56300 |
| C | -1.36019 | 0.12771 | -0.05747 |
| C | -1.19217 | 1.51954 | 0.09158 |
| C | 0.08039 | 2.12316 | -0.32543 |
| C | 1.11464 | 1.33817 | -0.78632 |
| C | -2.57469 | -0.47667 | 0.28758 |
| C | -2.26345 | 2.25803 | 0.61490 |
| H | 2.04423 | 1.77566 | -1.12872 |
| C | -3.46533 | 1.64860 | 0.96211 |
| C | -3.63082 | 0.27842 | 0.79575 |
| H | -2.16959 | 3.33011 | 0.76094 |
| H | -4.27577 | 2.24909 | 1.36240 |

| | | | |
|---|---|---|---|
| H | -4.56539 | -0.20959 | 1.05510 |
| C | -2.72262 | -1.91561 | 0.10465 |
| O | -3.66812 | -2.60579 | 0.33305 |
| N | -0.39307 | -1.97637 | -0.70527 |
| O | -1.60419 | -2.59857 | -0.42294 |
| S | 0.16929 | 3.85876 | -0.25120 |
| C | 1.85904 | 4.24188 | -0.76219 |
| H | 1.93371 | 5.33048 | -0.68871 |
| H | 2.58752 | 3.78784 | -0.08492 |
| H | 2.03856 | 3.94219 | -1.79814 |
| O | 0.46168 | -2.75338 | -1.05874 |
| H | 1.70275 | -0.62944 | -1.44888 |
| O | 1.94424 | -0.77774 | 0.78541 |
| H | 2.85822 | -1.32681 | 0.57864 |
| H | 2.15973 | -0.01624 | 1.33768 |
| N | 4.04066 | -2.14654 | 0.33603 |
| C | 3.74106 | -3.18538 | -0.65868 |
| H | 3.44293 | -2.71603 | -1.60073 |
| H | 4.60707 | -3.83526 | -0.84216 |
| H | 2.90937 | -3.79758 | -0.29823 |
| C | 4.47501 | -2.70960 | 1.62111 |
| H | 5.35109 | -3.36172 | 1.50875 |
| H | 4.72102 | -1.89657 | 2.31040 |
| H | 3.65417 | -3.29362 | 2.04983 |
| H | 4.78754 | -1.55828 | -0.02711 |

**Dimethylamine catalysed water addition to carbonyl C atom of acid chloride**

**Single point energy: -1812.1346267Hartrees**
**Gibbs free energy: --1811.884694 Hartrees**
**No. img. Freqs= 1**

| | | | |
|---|---|---|---|
| C | -1.44321 | -1.65795 | 1.13054 |
| C | -0.24410 | -1.39202 | 0.52699 |
| C | -0.11602 | -0.41529 | -0.51388 |
| C | -1.34076 | 0.09052 | -1.05665 |
| C | -2.59687 | -0.20671 | -0.40943 |
| C | -2.62231 | -1.02156 | 0.69967 |
| C | 1.11560 | 0.11053 | -1.02298 |
| C | -1.29527 | 0.89895 | -2.21978 |
| H | -3.54609 | -1.25280 | 1.21685 |
| C | -0.09802 | 1.22511 | -2.80654 |
| C | 1.10360 | 0.86353 | -2.17737 |
| H | -2.21793 | 1.26043 | -2.66329 |
| H | -0.07492 | 1.81529 | -3.71782 |
| H | 2.04323 | 1.22620 | -2.58371 |
| C | 2.38816 | 0.02535 | -0.19254 |
| O | 2.43031 | -0.37955 | 0.94049 |
| N | 0.82133 | -2.35435 | 0.83461 |
| O | 1.45701 | -2.79763 | -0.10379 |
| S | -4.05819 | 0.52841 | -1.08472 |
| C | -5.38074 | -0.12528 | -0.04079 |
| H | -6.30200 | 0.29729 | -0.45265 |
| H | -5.43525 | -1.21626 | -0.10040 |
| H | -5.27673 | 0.20331 | 0.99733 |
| O | 0.94501 | -2.72939 | 1.98625 |
| Cl | 3.88542 | -0.33871 | -1.27941 |
| H | -1.49474 | -2.41251 | 1.90917 |
| O | 2.71531 | 1.88935 | 0.04925 |
| H | 1.73263 | 2.14714 | 0.81727 |
| H | 2.73483 | 2.42319 | -0.75217 |
| N | 0.80047 | 2.27701 | 1.58780 |
| C | 1.28327 | 2.86171 | 2.84845 |
| H | 0.46432 | 2.97857 | 3.56638 |
| H | 2.05123 | 2.20755 | 3.26793 |
| H | 1.72479 | 3.83995 | 2.63803 |
| C | -0.26353 | 3.05532 | 0.93749 |
| H | -0.53929 | 2.56750 | -0.00173 |
| H | -1.14579 | 3.13487 | 1.58143 |
| H | 0.11604 | 4.05782 | 0.71936 |
| H | 0.46425 | 1.33167 | 1.77100 |

**Single point energy: -1812.13442096 Hartrees**
**Gibbs free energy: -1811.884751 Hartrees**
**No. img. Freqs= 1**

| | | | |
|---|---|---|---|
| C | 1.71717 | -2.00569 | -0.97157 |
| C | 0.49012 | -1.71098 | -0.44298 |
| C | 0.25421 | -0.52421 | 0.32446 |
| C | 1.41881 | 0.22387 | 0.69553 |
| C | 2.70869 | -0.11512 | 0.14281 |
| C | 2.82832 | -1.18093 | -0.71875 |
| C | -1.02676 | -0.00953 | 0.71036 |
| C | 1.28372 | 1.31113 | 1.59425 |
| H | 3.77821 | -1.45232 | -1.16370 |
| C | 0.05614 | 1.66790 | 2.09280 |
| C | -1.09724 | 1.02560 | 1.61727 |
| H | 2.16232 | 1.86860 | 1.90432 |
| H | -0.03265 | 2.48016 | 2.80832 |
| H | -2.06865 | 1.38299 | 1.94236 |
| C | -2.28907 | -0.47722 | 0.00369 |
| O | -2.29419 | -1.08234 | -1.03218 |
| N | -0.47483 | -2.81511 | -0.50923 |
| O | -1.06170 | -3.09944 | 0.51831 |
| S | 4.08960 | 0.89314 | 0.60390 |
| C | 5.48155 | 0.11555 | -0.24703 |
| H | 6.35577 | 0.70782 | 0.03933 |
| H | 5.62903 | -0.91563 | 0.08721 |

| | | | |
|---|---|---|---|
| H | 5.36709 | 0.16019 | -1.33404 |
| O | -0.56254 | -3.44479 | -1.54749 |
| Cl | -3.60197 | -1.00873 | 1.32023 |
| H | 1.84692 | -2.91945 | -1.54293 |
| O | -3.11081 | 1.13077 | -0.40856 |
| H | -2.43340 | 1.93756 | -0.99496 |
| H | -3.62013 | 1.51158 | 0.31454 |
| N | -1.73600 | 2.77184 | -1.66912 |
| C | -1.76397 | 4.07602 | -0.99251 |
| H | -1.18202 | 4.82526 | -1.54153 |
| H | -2.80010 | 4.41451 | -0.90567 |
| H | -1.34166 | 3.96124 | 0.01091 |
| C | -0.37263 | 2.25896 | -1.87418 |
| H | -0.42532 | 1.26269 | -2.32072 |
| H | 0.20508 | 2.92307 | -2.52741 |
| H | 0.12872 | 2.19056 | -0.90457 |
| H | -2.19393 | 2.85648 | -2.57495 |

**Single point energy: -1812.13460609 Hartrees**
**Gibbs free energy: -1811.884343 Hartrees**
**No. img. Freqs= 1**

| | | | |
|---|---|---|---|
| C | -1.45728 | -2.04572 | 0.96901 |
| C | -0.26171 | -1.68378 | 0.41136 |
| C | -0.12786 | -0.52056 | -0.41301 |
| C | -1.34787 | 0.11231 | -0.81584 |
| C | -2.60211 | -0.29284 | -0.22577 |
| C | -2.62956 | -1.31731 | 0.69290 |
| C | 1.10772 | 0.07195 | -0.82940 |
| C | -1.29943 | 1.14367 | -1.78762 |
| H | -3.55165 | -1.63549 | 1.16502 |
| C | -0.10367 | 1.55018 | -2.32674 |
| C | 1.09791 | 1.03818 | -1.81214 |
| H | -2.21875 | 1.61158 | -2.12662 |
| H | -0.08013 | 2.31116 | -3.10130 |
| H | 2.03993 | 1.45563 | -2.15380 |
| C | 2.38404 | -0.18650 | -0.04022 |
| O | 2.43058 | -0.79246 | 0.99466 |
| N | 0.79419 | -2.69898 | 0.50900 |
| O | 1.42168 | -2.94709 | -0.50419 |
| S | -4.05885 | 0.58190 | -0.72127 |
| C | -5.38255 | -0.25680 | 0.17885 |
| H | -6.30025 | 0.25584 | -0.12453 |
| H | -5.45500 | -1.31013 | -0.10725 |
| H | -5.26401 | -0.15421 | 1.26138 |
| O | 0.91921 | -3.30159 | 1.55912 |
| Cl | 3.86234 | -0.42602 | -1.23576 |
| H | -1.51244 | -2.93848 | 1.58381 |
| O | 2.79471 | 1.57373 | 0.45753 |
| H | 1.73252 | 2.08367 | 0.85108 |
| H | 3.21945 | 2.08037 | -0.24299 |
| N | 0.65743 | 2.50302 | 1.32055 |
| C | 0.32398 | 1.70051 | 2.50883 |
| H | -0.61914 | 2.03383 | 2.95607 |
| H | 0.24459 | 0.64917 | 2.22063 |
| H | 1.13116 | 1.80146 | 3.23955 |
| C | 0.77316 | 3.94213 | 1.59839 |
| H | 0.99974 | 4.46933 | 0.66763 |
| H | -0.15430 | 4.33887 | 2.02599 |
| H | 1.59297 | 4.09982 | 2.30520 |
| H | -0.06220 | 2.35797 | 0.61309 |

**Single point energy: -1812.13520434 Hartrees**
**Gibbs free energy: -1811.885807 Hartrees**
**No. img. Freqs= 1**

| | | | |
|---|---|---|---|
| C | 2.01696 | 2.06286 | 0.22062 |
| C | 0.90325 | 1.40897 | -0.23285 |
| C | 0.84515 | -0.01826 | -0.33798 |
| C | 2.08426 | -0.71264 | -0.15026 |
| C | 3.25512 | -0.00130 | 0.30411 |
| C | 3.19014 | 1.35421 | 0.53443 |
| C | -0.33148 | -0.79968 | -0.58260 |
| C | 2.14009 | -2.10556 | -0.40797 |
| H | 4.04589 | 1.91529 | 0.89053 |
| C | 1.02622 | -2.79765 | -0.81069 |
| C | -0.21575 | -2.14472 | -0.86079 |
| H | 3.07910 | -2.63822 | -0.29181 |
| H | 1.08863 | -3.85895 | -1.03249 |
| H | -1.11637 | -2.71372 | -1.06489 |
| C | -1.71258 | -0.20716 | -0.37836 |
| O | -1.93317 | 0.80452 | 0.23890 |
| N | -0.11083 | 2.28774 | -0.82579 |
| O | -0.54181 | 1.97976 | -1.92187 |
| S | 4.73877 | -0.93591 | 0.55217 |
| C | 5.92995 | 0.30396 | 1.10858 |
| H | 6.86235 | -0.24741 | 1.26233 |
| H | 6.09572 | 1.07237 | 0.34772 |
| H | 5.62728 | 0.75514 | 2.05816 |
| O | -0.39357 | 3.31932 | -0.24313 |
| Cl | -2.80281 | -0.52357 | -1.90329 |
| H | 2.01336 | 3.14670 | 0.28004 |
| O | -2.50323 | -1.61183 | 0.62879 |
| H | -3.52463 | -1.12162 | 1.11388 |
| H | -1.88313 | -1.77094 | 1.35085 |
| N | -4.54488 | -0.59484 | 1.63624 |
| C | -4.08881 | 0.20845 | 2.78161 |
| H | -4.93035 | 0.70246 | 3.28020 |
| H | -3.57590 | -0.44227 | 3.49560 |
| H | -3.38611 | 0.96296 | 2.41716 |
| C | -5.28717 | 0.19752 | 0.64221 |
| H | -5.61417 | -0.45844 | -0.16794 |

| | | | |
|---|---|---|---|
| H | -6.15888 | 0.67946 | 1.10020 |
| H | -4.61944 | 0.96007 | 0.23336 |
| H | -5.14386 | -1.34600 | 1.97331 |

**Single point energy: -1812.13661014 Hartrees**
**Gibbs free energy: -1811.888072 Hartrees**
**No. img. Freqs= 1**

| | | | |
|---|---|---|---|
| C | 2.23534 | 2.00330 | 0.35547 |
| C | 1.07173 | 1.44437 | -0.10016 |
| C | 0.92312 | 0.03158 | -0.28303 |
| C | 2.12043 | -0.74643 | -0.16561 |
| C | 3.34377 | -0.13404 | 0.29427 |
| C | 3.36995 | 1.20881 | 0.59572 |
| C | -0.30445 | -0.66328 | -0.53913 |
| C | 2.08301 | -2.12427 | -0.49758 |
| H | 4.26802 | 1.69635 | 0.95587 |
| C | 0.91912 | -2.72504 | -0.90511 |
| C | -0.28067 | -1.99626 | -0.88782 |
| H | 2.98910 | -2.71929 | -0.43509 |
| H | 0.91001 | -3.77496 | -1.18231 |
| H | -1.22008 | -2.49763 | -1.09271 |
| C | -1.63825 | 0.00170 | -0.27479 |
| O | -1.79537 | 0.97250 | 0.42022 |
| N | 0.09566 | 2.41486 | -0.60737 |
| O | -0.38118 | 2.20114 | -1.70717 |
| S | 4.77030 | -1.17015 | 0.45734 |
| C | 6.04512 | -0.04005 | 1.06042 |
| H | 6.94228 | -0.65678 | 1.16901 |
| H | 6.25051 | 0.75720 | 0.33988 |
| H | 5.78348 | 0.37631 | 2.03774 |
| O | -0.11356 | 3.42106 | 0.04648 |
| Cl | -2.76703 | -0.12677 | -1.81078 |
| H | 2.30043 | 3.08020 | 0.47555 |
| O | -2.53622 | -1.42333 | 0.62644 |
| H | -3.58470 | -0.85268 | 0.87110 |
| H | -2.03611 | -1.52430 | 1.44548 |
| N | -4.66158 | -0.21113 | 1.06761 |
| C | -5.80767 | -0.97212 | 0.55379 |
| H | -6.74738 | -0.42729 | 0.70206 |
| H | -5.66027 | -1.16158 | -0.51258 |
| H | -5.86137 | -1.93056 | 1.07906 |
| C | -4.76830 | 0.12252 | 2.49413 |
| H | -3.89213 | 0.70601 | 2.78948 |
| H | -5.67638 | 0.69964 | 2.70325 |
| H | -4.79230 | -0.80483 | 3.07524 |
| H | -4.55976 | 0.64983 | 0.53184 |

**Single point energy: -1812.13518239 Hartrees**
**Gibbs free energy: -1811.886904 Hartrees**
**No. img. Freqs= 1**

| | | | |
|---|---|---|---|
| C | 2.40781 | 2.02646 | 0.23561 |
| C | 1.21686 | 1.50089 | -0.18770 |
| C | 0.99686 | 0.08875 | -0.28755 |
| C | 2.15530 | -0.73932 | -0.12641 |
| C | 3.40931 | -0.16259 | 0.29532 |
| C | 3.50240 | 1.19173 | 0.52167 |
| C | -0.26478 | -0.55812 | -0.50157 |
| C | 2.04960 | -2.13039 | -0.37782 |
| H | 4.42482 | 1.65280 | 0.85452 |
| C | 0.85625 | -2.69535 | -0.74961 |
| C | -0.30608 | -1.90853 | -0.77386 |
| H | 2.92584 | -2.76434 | -0.28199 |
| H | 0.79478 | -3.75768 | -0.96653 |
| H | -1.26869 | -2.37432 | -0.95261 |
| C | -1.56497 | 0.18818 | -0.27343 |
| O | -1.66186 | 1.21104 | 0.35274 |
| N | 0.29274 | 2.48868 | -0.75632 |
| O | -0.19120 | 2.23447 | -1.84404 |
| S | 4.78548 | -1.25574 | 0.51165 |
| C | 6.12999 | -0.14834 | 0.99280 |
| H | 7.00180 | -0.79764 | 1.11705 |
| H | 6.34523 | 0.58501 | 0.21000 |
| H | 5.92346 | 0.34860 | 1.94520 |
| O | 0.13573 | 3.54198 | -0.16512 |
| Cl | -2.68938 | 0.02990 | -1.82343 |
| H | 2.52669 | 3.10393 | 0.29244 |
| O | -2.51159 | -1.11625 | 0.68494 |
| H | -3.62835 | -0.72138 | 0.89757 |
| H | -2.03980 | -1.15732 | 1.52603 |
| N | -4.84539 | -0.34719 | 1.10233 |
| C | -4.94644 | 1.11883 | 1.02352 |
| H | -5.97220 | 1.45403 | 1.21786 |
| H | -4.26863 | 1.56573 | 1.75468 |
| H | -4.64001 | 1.44138 | 0.02532 |
| C | -5.73363 | -1.03951 | 0.15647 |
| H | -5.60816 | -2.11956 | 0.27140 |
| H | -6.78319 | -0.77240 | 0.32786 |
| H | -5.45143 | -0.75863 | -0.86181 |
| H | -5.08680 | -0.63986 | 2.04747 |

**Single point energy: -1812.13810591 Hartrees**
**Gibbs free energy: -1811.887572 Hartrees**
**No. img. Freqs= 1**

| | | | |
|---|---|---|---|
| C | -1.47139 | -2.03536 | 0.97991 |
| C | -0.27294 | -1.67080 | 0.42908 |
| C | -0.13937 | -0.51749 | -0.40834 |
| C | -1.35871 | 0.10948 | -0.82092 |
| C | -2.61589 | -0.30153 | -0.24257 |
| C | -2.64618 | -1.32066 | 0.68261 |
| C | 1.09547 | 0.07666 | -0.82421 |

| | | | |
|---|---|---|---|
| C | -1.30628 | 1.14489 | -1.78930 |
| H | -3.57108 | -1.64249 | 1.14668 |
| C | -0.10920 | 1.56205 | -2.31597 |
| C | 1.09252 | 1.05194 | -1.79756 |
| H | -2.22533 | 1.60976 | -2.13336 |
| H | -0.08437 | 2.33127 | -3.08234 |
| H | 2.03843 | 1.48027 | -2.11137 |
| C | 2.37191 | -0.17027 | -0.03859 |
| O | 2.42070 | -0.72898 | 1.02935 |
| N | 0.79291 | -2.67065 | 0.56149 |
| O | 1.43457 | -2.93512 | -0.43844 |
| S | -4.07431 | 0.55876 | -0.75845 |
| C | -5.39966 | -0.27918 | 0.13992 |
| H | -6.31798 | 0.22578 | -0.17429 |
| H | -5.46489 | -1.33542 | -0.13711 |
| H | -5.28914 | -0.16624 | 1.22230 |
| O | 0.91425 | -3.24645 | 1.62749 |
| Cl | 3.83557 | -0.49426 | -1.21530 |
| H | -1.52506 | -2.91966 | 1.60699 |
| O | 2.76450 | 1.62742 | 0.28206 |
| H | 1.76655 | 2.05845 | 0.84108 |
| H | 3.50936 | 1.56741 | 0.89200 |
| N | 0.68262 | 2.48286 | 1.34498 |
| C | 0.34074 | 1.67457 | 2.52551 |
| H | -0.59333 | 2.01902 | 2.98377 |
| H | 0.24122 | 0.62772 | 2.22770 |
| H | 1.15200 | 1.75320 | 3.25531 |
| C | 0.80848 | 3.91871 | 1.63359 |
| H | 1.04495 | 4.44977 | 0.70771 |
| H | -0.11682 | 4.32140 | 2.06122 |
| H | 1.62621 | 4.06653 | 2.34544 |
| H | -0.03793 | 2.35058 | 0.63545 |

**Single point energy: -1812.13867974 Hartrees**
**Gibbs free energy: -1811.888897 Hartrees**
**No. img. Freqs= 1**

| | | | |
|---|---|---|---|
| C | 1.43701 | 2.08663 | 0.82968 |
| C | 0.25165 | 1.64052 | 0.31123 |
| C | 0.15667 | 0.42113 | -0.43457 |
| C | 1.39932 | -0.18532 | -0.80861 |
| C | 2.64121 | 0.31021 | -0.26458 |
| C | 2.63641 | 1.39387 | 0.58418 |
| C | -1.05416 | -0.25299 | -0.79690 |
| C | 1.38707 | -1.28646 | -1.70171 |
| H | 3.54933 | 1.78088 | 1.02133 |
| C | 0.20664 | -1.80063 | -2.17597 |
| C | -1.01187 | -1.30825 | -1.68241 |
| H | 2.32407 | -1.73267 | -2.02112 |
| H | 0.21071 | -2.62867 | -2.87866 |
| H | -1.94025 | -1.79748 | -1.95589 |
| C | -2.35607 | 0.05099 | -0.07859 |
| O | -2.44876 | 0.69822 | 0.93357 |
| N | -0.84051 | 2.61889 | 0.36593 |
| O | -1.46979 | 2.80553 | -0.65883 |
| S | 4.12894 | -0.53619 | -0.71608 |
| C | 5.41897 | 0.39460 | 0.14156 |
| H | 6.35520 | -0.10334 | -0.12780 |
| H | 5.46026 | 1.43240 | -0.20177 |
| H | 5.29577 | 0.34792 | 1.22757 |
| O | -0.99295 | 3.25744 | 1.39167 |
| Cl | -3.77237 | 0.31041 | -1.36086 |
| H | -4.10699 | -2.86856 | 2.50705 |
| H | -5.38342 | -1.69183 | 2.11387 |
| C | -4.96438 | -1.72453 | -0.56362 |
| H | -4.36307 | -1.47826 | -1.44230 |
| H | -5.68033 | -2.51451 | -0.81976 |
| H | -5.51085 | -0.82900 | -0.25465 |
| H | -3.51252 | -2.93159 | 0.21989 |

**Single point energy: -1812.13833946 Hartrees**
**Gibbs free energy: -1811.888529 Hartrees**
**No. img. Freqs= 1**

| | | | |
|---|---|---|---|
| C | 1.35626 | 2.12455 | -0.09909 |
| C | 0.43279 | 1.39138 | -0.79450 |
| C | 0.33261 | -0.03107 | -0.66456 |
| C | 1.38576 | -0.67112 | 0.06525 |
| C | 2.36524 | 0.11857 | 0.77182 |
| C | 2.30402 | 1.49299 | 0.72536 |
| C | -0.74085 | -0.84814 | -1.14803 |
| C | 1.43793 | -2.08758 | 0.10095 |
| H | 3.01297 | 2.11482 | 1.25906 |
| C | 0.48457 | -2.84776 | -0.52739 |
| C | -0.62484 | -2.22064 | -1.11735 |
| H | 2.24773 | -2.58458 | 0.62650 |
| H | 0.55149 | -3.93174 | -0.51825 |
| H | -1.43904 | -2.82214 | -1.50541 |
| C | -2.07743 | -0.23072 | -1.51301 |
| O | -2.42737 | 0.87580 | -1.18486 |
| N | -0.27017 | 2.14101 | -1.84165 |
| O | -0.33075 | 1.63118 | -2.94497 |
| S | 3.60676 | -0.74449 | 1.69301 |
| C | 4.63929 | 0.58319 | 2.35442 |
| H | 5.43439 | 0.07509 | 2.90817 |
| H | 5.09074 | 1.17611 | 1.55350 |
| H | 4.08325 | 1.22256 | 3.04631 |
| O | -0.66695 | 3.26278 | -1.58096 |
| Cl | -2.60229 | -0.78683 | -3.28042 |
| H | 1.39368 | 3.20116 | -0.23218 |
| O | -3.18000 | -1.42348 | -0.63330 |
| H | -3.15819 | -1.09985 | 0.51328 |

| | | | |
|---|---|---|---|
| H | -4.04965 | -1.22306 | -1.00013 |
| N | -3.10225 | -0.73075 | 1.76071 |
| C | -1.71194 | -0.44522 | 2.14710 |
| H | -1.63852 | -0.20027 | 3.21322 |
| H | -1.08834 | -1.31730 | 1.92936 |
| H | -1.35239 | 0.40553 | 1.56173 |
| C | -3.99243 | 0.42690 | 1.93594 |
| H | -5.01457 | 0.13650 | 1.67743 |
| H | -3.97006 | 0.79668 | 2.96786 |
| H | -3.66847 | 1.22170 | 1.25761 |
| H | -3.44709 | -1.50501 | 2.32490 |

## Dimethylamine catalysed water addition to C7 atom of acid chloride

**Single point energy: -1812.12735337 Hartrees**
**Gibbs free energy: - 1811.875967 Hartrees**
**No. img. Freqs= 1**

| | | | |
|---|---|---|---|
| C | -0.58726 | 1.08098 | -1.66977 |
| C | 0.56383 | 0.61365 | -0.98858 |
| C | 0.48235 | -0.45254 | -0.02342 |
| C | -0.81498 | -0.97538 | 0.27198 |
| C | -1.95176 | -0.59192 | -0.56433 |
| C | -1.80425 | 0.33175 | -1.55027 |
| C | 1.57861 | -1.04905 | 0.66881 |
| C | -0.98310 | -1.87933 | 1.33961 |
| H | -2.62052 | 0.60863 | -2.20582 |
| C | 0.08462 | -2.31699 | 2.09632 |
| C | 1.37279 | -1.92684 | 1.72666 |
| H | -1.97754 | -2.24403 | 1.58040 |
| H | -0.06642 | -2.99866 | 2.92750 |
| H | 2.23593 | -2.34206 | 2.23952 |
| C | 3.00785 | -0.96328 | 0.26462 |
| O | 3.95570 | -0.87541 | 0.97295 |
| N | 1.68513 | 1.46101 | -1.09249 |
| O | 2.64364 | 1.31549 | -0.31787 |
| S | -3.48542 | -1.42157 | -0.25614 |
| C | -4.62995 | -0.62116 | -1.40272 |
| H | -5.60455 | -1.07350 | -1.19633 |
| H | -4.69083 | 0.45568 | -1.21886 |
| H | -4.36158 | -0.82141 | -2.44405 |
| O | 1.66931 | 2.38615 | -1.90986 |
| Cl | 3.27655 | -1.40336 | -1.48865 |
| H | -0.44052 | 1.65640 | -2.57385 |
| O | -1.14143 | 2.72575 | -0.75333 |
| H | -1.03088 | 2.42973 | 0.57834 |
| H | -0.51978 | 3.40248 | -1.04481 |
| N | -0.98640 | 2.16041 | 1.69956 |
| C | -2.08467 | 2.85493 | 2.39445 |
| H | -3.03433 | 2.54087 | 1.95517 |
| H | -2.07313 | 2.61978 | 3.46299 |
| H | -1.96253 | 3.93212 | 2.25390 |
| C | 0.35280 | 2.49050 | 2.22069 |
| H | 0.41576 | 2.26019 | 3.28830 |
| H | 1.10431 | 1.91378 | 1.67591 |
| H | 0.53627 | 3.55674 | 2.06321 |
| H | -1.13318 | 1.15311 | 1.76954 |

**Single point energy: -1812.12421629 Hartrees**
**Gibbs free energy: -1811.873101 Hartrees**
**No. img. Freqs= 1**

| | | | |
|---|---|---|---|
| C | -0.70642 | 0.88538 | -1.74411 |
| C | 0.46594 | 0.45376 | -1.07646 |
| C | 0.40581 | -0.53345 | -0.03507 |
| C | -0.87755 | -1.08091 | 0.28338 |
| C | -2.01452 | -0.79915 | -0.58897 |
| C | -1.89433 | 0.08663 | -1.61264 |
| C | 1.51219 | -1.04029 | 0.71551 |
| C | -1.02798 | -1.91567 | 1.40793 |
| H | -2.71000 | 0.29139 | -2.29629 |
| C | 0.04165 | -2.26153 | 2.20778 |
| C | 1.32081 | -1.84878 | 1.82978 |
| H | -2.01357 | -2.29473 | 1.66293 |
| H | -0.09879 | -2.88720 | 3.08368 |
| H | 2.18989 | -2.19151 | 2.38469 |
| C | 2.94520 | -0.91656 | 0.33883 |
| O | 3.86871 | -0.73108 | 1.06086 |
| N | 1.58936 | 1.29411 | -1.27154 |
| O | 2.47292 | 1.33674 | -0.40133 |
| S | -3.50699 | -1.69962 | -0.26941 |
| C | -4.66722 | -1.03002 | -1.48178 |
| H | -5.61591 | -1.53658 | -1.28087 |
| H | -4.80564 | 0.04735 | -1.35097 |
| H | -4.35640 | -1.25838 | -2.50542 |
| O | 1.65169 | 2.00203 | -2.27397 |
| Cl | 3.28264 | -1.44491 | -1.37553 |
| H | -0.57925 | 1.45178 | -2.65657 |
| O | -1.16614 | 2.50238 | -0.79002 |
| H | -0.87924 | 2.31708 | 0.59255 |
| H | -2.07514 | 2.72604 | -1.01756 |
| N | -0.59558 | 2.18856 | 1.67274 |
| C | -1.79693 | 1.79377 | 2.43098 |
| H | -2.20831 | 0.88259 | 1.98978 |
| H | -1.54477 | 1.61805 | 3.48026 |
| H | -2.53501 | 2.59679 | 2.35889 |
| C | 0.03731 | 3.43297 | 2.15126 |
| H | 0.32669 | 3.33108 | 3.20089 |

| | | | |
|---|---|---|---|
| H | 0.91842 | 3.63567 | 1.53907 |
| H | -0.67746 | 4.25232 | 2.04131 |
| H | 0.08876 | 1.43250 | 1.71123 |

**Single point energy: -1812.12629852 Hartrees**
**Gibbs free energy: -1811.874617 Hartrees**
**No. img. Freqs= 1**

| | | | |
|---|---|---|---|
| C | -0.85207 | 1.03382 | -1.59717 |
| C | 0.27952 | 0.37605 | -1.05950 |
| C | 0.13418 | -0.62256 | -0.03540 |
| C | -1.19569 | -0.97662 | 0.35267 |
| C | -2.32945 | -0.48486 | -0.42695 |
| C | -2.14146 | 0.43470 | -1.41139 |
| C | 1.19479 | -1.31001 | 0.62977 |
| C | -1.40282 | -1.82258 | 1.45941 |
| H | -2.95839 | 0.80790 | -2.01783 |
| C | -0.35129 | -2.35360 | 2.17770 |
| C | 0.95085 | -2.11934 | 1.73334 |
| H | -2.41652 | -2.05763 | 1.77043 |
| H | -0.53243 | -2.98224 | 3.04391 |
| H | 1.78957 | -2.60280 | 2.22657 |
| C | 2.60994 | -1.37193 | 0.17765 |
| O | 3.58459 | -1.33118 | 0.85400 |
| N | 1.49882 | 1.04059 | -1.31411 |
| O | 2.41082 | 0.98177 | -0.46210 |
| S | -3.91609 | -1.17516 | -0.05214 |
| C | -5.03249 | -0.27708 | -1.15265 |
| H | -6.03197 | -0.65563 | -0.91878 |
| H | -5.00648 | 0.79919 | -0.95690 |
| H | -4.81363 | -0.48486 | -2.20410 |
| O | 1.63050 | 1.71717 | -2.32861 |
| Cl | 2.78804 | -1.87120 | -1.56594 |
| H | -0.70676 | 1.65694 | -2.46820 |
| O | -0.96130 | 2.65237 | -0.47717 |
| H | -0.06243 | 2.50715 | 0.67835 |
| H | -1.89415 | 2.74397 | -0.25731 |
| N | 0.68665 | 2.42815 | 1.49460 |
| C | 0.05634 | 1.83286 | 2.68721 |
| H | -0.32955 | 0.84343 | 2.42937 |
| H | 0.78803 | 1.74487 | 3.49473 |
| H | -0.77007 | 2.47332 | 3.00604 |
| C | 1.26677 | 3.76538 | 1.72402 |
| H | 2.02436 | 3.71693 | 2.51095 |
| H | 1.71934 | 4.11302 | 0.79290 |
| H | 0.46770 | 4.45022 | 2.01857 |
| H | 1.42395 | 1.80442 | 1.14928 |

**Single point energy: -1812.12470169 Hartrees**
**Gibbs free energy: -1811.873659 Hartrees**
**No. img. Freqs= 1**

| | | | |
|---|---|---|---|
| C | -0.18353 | -1.25820 | -1.03344 |
| C | -0.34622 | 0.07431 | -0.58671 |
| C | 0.69377 | 0.75406 | 0.14212 |
| C | 1.88579 | 0.01835 | 0.42453 |
| C | 2.09782 | -1.27960 | -0.21276 |
| C | 1.12510 | -1.84107 | -0.98025 |
| C | 0.66265 | 2.10853 | 0.59103 |
| C | 2.86812 | 0.56892 | 1.27089 |
| H | 1.26712 | -2.78517 | -1.49157 |
| C | 2.73670 | 1.83024 | 1.81360 |
| C | 1.64414 | 2.61228 | 1.43655 |
| H | 3.75383 | -0.01162 | 1.51225 |
| H | 3.49423 | 2.22887 | 2.48121 |
| H | 1.57145 | 3.64373 | 1.77010 |
| C | -0.26322 | 3.16495 | 0.10421 |
| O | -0.77831 | 4.02738 | 0.73512 |
| N | -1.66960 | 0.54788 | -0.64930 |
| O | -2.01023 | 1.52821 | 0.02992 |
| S | 3.68331 | -2.03518 | 0.01911 |
| C | 3.51877 | -3.62345 | -0.82698 |
| H | 4.46810 | -4.13926 | -0.65454 |
| H | 2.70708 | -4.22039 | -0.40046 |
| H | 3.37894 | -3.49391 | -1.90417 |
| O | -2.50006 | -0.05874 | -1.33552 |
| Cl | -0.28581 | 3.30399 | -1.72182 |
| H | -0.86988 | -1.62626 | -1.78281 |
| O | -1.14418 | -2.38426 | 0.25788 |
| H | -2.51793 | -2.26171 | 0.57017 |
| H | -0.57337 | -2.33300 | 1.03200 |
| N | -3.61808 | -2.25368 | 0.82748 |
| C | -4.42007 | -2.52328 | -0.38211 |
| H | -4.08585 | -3.46533 | -0.82271 |
| H | -5.48112 | -2.58911 | -0.12418 |
| H | -4.25821 | -1.71008 | -1.09192 |
| C | -3.97180 | -0.99052 | 1.50621 |
| H | -4.99777 | -1.03943 | 1.88234 |
| H | -3.28012 | -0.83001 | 2.33661 |
| H | -3.88007 | -0.16902 | 0.79263 |
| H | -3.74622 | -3.02427 | 1.48296 |

**Single point energy: -1812.12703307 Hartrees**
**Gibbs free energy: -1811.876698 Hartrees**
**No. img. Freqs= 1**

| | | | |
|---|---|---|---|
| C | -1.64972 | 0.20253 | -2.03274 |
| C | -0.45966 | 0.31493 | -1.27539 |
| C | -0.30481 | -0.36827 | -0.01477 |
| C | -1.41651 | -1.12947 | 0.45829 |
| C | -2.55426 | -1.37064 | -0.42679 |
| C | -2.61203 | -0.78800 | -1.65498 |
| C | 0.86625 | -0.38038 | 0.80005 |

| | | | |
|---|---|---|---|
| C | -1.38863 | -1.68573 | 1.75214 |
| H | -3.42782 | -0.97304 | -2.34295 |
| C | -0.29196 | -1.55162 | 2.57770 |
| C | 0.85150 | -0.92698 | 2.07811 |
| H | -2.25320 | -2.23213 | 2.11773 |
| H | -0.29754 | -1.96814 | 3.58014 |
| H | 1.76010 | -0.89808 | 2.67302 |
| C | 2.23546 | -0.00115 | 0.36119 |
| O | 3.06216 | 0.58031 | 0.98194 |
| N | 0.39963 | 1.34530 | -1.69150 |
| O | 1.26690 | 1.78235 | -0.92224 |
| S | -3.79719 | -2.48768 | 0.15722 |
| C | -5.04071 | -2.45461 | -1.15346 |
| H | -5.85115 | -3.09665 | -0.79551 |
| H | -5.42996 | -1.44387 | -1.30792 |
| H | -4.65128 | -2.86701 | -2.08874 |
| O | 0.23895 | 1.85633 | -2.81068 |
| Cl | 2.75168 | -0.85186 | -1.17495 |
| H | -1.62244 | 0.51214 | -3.06765 |
| O | -2.67887 | 1.86643 | -1.69334 |
| H | -2.03222 | 3.09806 | -1.95339 |
| H | -2.97501 | 1.75139 | -0.78419 |
| N | -1.50516 | 4.06570 | -2.19712 |
| C | -1.13493 | 4.72784 | -0.93334 |
| H | -0.49968 | 4.05322 | -0.35469 |
| H | -0.59590 | 5.65804 | -1.13401 |
| H | -2.04616 | 4.94432 | -0.36912 |
| C | -2.35452 | 4.88510 | -3.07786 |
| H | -1.85004 | 5.82268 | -3.32938 |
| H | -2.56413 | 4.32121 | -3.98951 |
| H | -3.29423 | 5.09872 | -2.56177 |
| H | -0.65552 | 3.78518 | -2.69098 |

**Single point energy: -1812.12331978 Hartrees**
**Gibbs free energy: -1811.873663 Hartrees**
**No. img. Freqs= 1**

| | | | |
|---|---|---|---|
| C | -0.17133 | -1.26658 | -1.06414 |
| C | -0.35054 | 0.06257 | -0.61276 |
| C | 0.67912 | 0.74909 | 0.12777 |
| C | 1.87046 | 0.01927 | 0.42297 |
| C | 2.09597 | -1.27914 | -0.20894 |
| C | 1.13565 | -1.84621 | -0.98835 |
| C | 0.63607 | 2.10300 | 0.57560 |
| C | 2.84245 | 0.57525 | 1.27757 |
| H | 1.28734 | -2.79240 | -1.49309 |
| C | 2.69991 | 1.83629 | 1.81767 |
| C | 1.60699 | 2.61253 | 1.43007 |
| H | 3.72849 | -0.00168 | 1.52614 |
| H | 3.44896 | 2.23917 | 2.49221 |
| H | 1.52601 | 3.64352 | 1.76295 |
| C | -0.29121 | 3.15434 | 0.07964 |
| O | -0.81624 | 4.01398 | 0.70593 |
| N | -1.67456 | 0.52677 | -0.68136 |
| O | -2.02973 | 1.50004 | -0.00201 |
| S | 3.68014 | -2.02675 | 0.04634 |
| C | 3.53714 | -3.61447 | -0.80454 |
| H | 4.48639 | -4.12573 | -0.61820 |
| H | 2.72236 | -4.21618 | -0.39095 |
| H | 3.41318 | -3.48360 | -1.88341 |
| O | -2.49857 | -0.08632 | -1.37752 |
| Cl | -0.30565 | 3.28720 | -1.74576 |
| H | -0.84692 | -1.63997 | -1.82031 |
| O | -1.16257 | -2.42089 | 0.21174 |
| H | -2.53446 | -2.13991 | 0.41556 |
| H | -0.92036 | -3.35232 | 0.24756 |
| N | -3.63353 | -1.91484 | 0.53789 |
| C | -3.83771 | -1.28198 | 1.85335 |
| H | -3.22895 | -0.37644 | 1.90734 |
| H | -4.89149 | -1.02499 | 1.99330 |
| H | -3.52208 | -1.98062 | 2.63300 |
| C | -4.41732 | -3.14497 | 0.33537 |
| H | -5.48618 | -2.93786 | 0.44246 |
| H | -4.21354 | -3.53186 | -0.66554 |
| H | -4.10879 | -3.88543 | 1.07803 |
| H | -3.84688 | -1.23792 | -0.19745 |

**Single point energy: -1812.12682904 Hartrees**
**Gibbs free energy: -1811.876291 Hartrees**
**No. img. Freqs= 1**

| | | | |
|---|---|---|---|
| C | -0.65218 | 1.04284 | -1.60498 |
| C | 0.51393 | 0.62265 | -0.91829 |
| C | 0.49833 | -0.51957 | -0.04054 |
| C | -0.75784 | -1.16074 | 0.18432 |
| C | -1.90962 | -0.79315 | -0.63775 |
| C | -1.81476 | 0.20471 | -1.55515 |
| C | 1.62707 | -1.08264 | 0.62612 |
| C | -0.87037 | -2.16064 | 1.16922 |
| H | -2.63895 | 0.46731 | -2.20674 |
| C | 0.21608 | -2.57576 | 1.91216 |
| C | 1.47473 | -2.05714 | 1.60593 |
| H | -1.83653 | -2.61890 | 1.35954 |
| H | 0.10509 | -3.33279 | 2.68228 |
| H | 2.35950 | -2.44454 | 2.10348 |
| C | 3.05100 | -0.85521 | 0.26164 |
| O | 3.97897 | -0.75073 | 0.99365 |
| N | 1.56636 | 1.55647 | -0.93154 |
| O | 2.52849 | 1.41855 | -0.16036 |
| S | -3.38714 | -1.74316 | -0.41311 |
| C | -4.57325 | -0.93755 | -1.51252 |
| H | -5.51719 | -1.46708 | -1.35259 |

| | | | |
|---|---|---|---|
| H | -4.70696 | 0.11641 | -1.25090 |
| H | -4.28422 | -1.04158 | -2.56231 |
| O | 1.48629 | 2.54648 | -1.66811 |
| Cl | 3.38192 | -1.14371 | -1.51488 |
| H | -0.52361 | 1.66664 | -2.47990 |
| O | -1.33460 | 2.60733 | -0.65555 |
| H | -1.26737 | 2.55136 | 0.69772 |
| H | -0.78820 | 3.33110 | -0.98306 |
| N | -1.26187 | 2.51711 | 1.85813 |
| C | 0.11047 | 2.27570 | 2.34293 |
| H | 0.77631 | 3.03046 | 1.91837 |
| H | 0.14448 | 2.32372 | 3.43553 |
| H | 0.43341 | 1.28556 | 2.01305 |
| C | -2.23976 | 1.51849 | 2.32643 |
| H | -2.32004 | 1.54191 | 3.41746 |
| H | -3.21172 | 1.73506 | 1.87681 |
| H | -1.90818 | 0.52721 | 2.00846 |
| H | -1.56196 | 3.44184 | 2.16527 |

**Single point energy: -1812.12708366 Hartrees**
**Gibbs free energy: -1811.876115 Hartrees**
**No. img. Freqs= 1**

| | | | |
|---|---|---|---|
| C | -1.98885 | 0.61013 | -1.88002 |
| C | -0.64475 | 0.51473 | -1.44768 |
| C | -0.26931 | -0.33889 | -0.34757 |
| C | -1.31446 | -1.05131 | 0.31527 |
| C | -2.65421 | -1.07371 | -0.26912 |
| C | -2.93970 | -0.33369 | -1.37474 |
| C | 1.05583 | -0.56240 | 0.13130 |
| C | -1.03680 | -1.76978 | 1.49479 |
| H | -3.91391 | -0.35530 | -1.84734 |
| C | 0.23704 | -1.84244 | 2.01886 |
| C | 1.28964 | -1.26547 | 1.30738 |
| H | -1.84292 | -2.27920 | 2.01467 |
| H | 0.42736 | -2.38253 | 2.94103 |
| H | 2.31331 | -1.39637 | 1.64683 |
| C | 2.30998 | -0.25613 | -0.60660 |
| O | 3.32927 | 0.16023 | -0.16527 |
| N | 0.20250 | 1.51133 | -1.95854 |
| O | 1.27876 | 1.76270 | -1.39726 |
| S | -3.84125 | -2.14201 | 0.49456 |
| C | -5.34833 | -1.84460 | -0.45673 |
| H | -6.11482 | -2.46091 | 0.02256 |
| H | -5.65302 | -0.79510 | -0.40524 |
| H | -5.23776 | -2.16551 | -1.49666 |
| O | -0.16725 | 2.18362 | -2.93357 |
| Cl | 2.32209 | -0.94062 | -2.30361 |
| H | -2.18018 | 1.05175 | -2.84744 |
| O | -2.69504 | 2.29800 | -1.10692 |
| H | -1.94043 | 3.47781 | -1.31616 |
| H | -2.81539 | 2.06650 | -0.17982 |
| N | -1.33522 | 4.41070 | -1.50938 |
| C | -0.61393 | 4.76029 | -0.27205 |
| H | 0.00899 | 3.91295 | 0.02432 |
| H | 0.01511 | 5.64028 | -0.43305 |
| H | -1.34497 | 4.96844 | 0.51402 |
| C | -2.21561 | 5.47860 | -2.01291 |
| H | -1.63897 | 6.38764 | -2.20750 |
| H | -2.69155 | 5.13733 | -2.93487 |
| H | -2.98513 | 5.68330 | -1.26388 |
| H | -0.66100 | 4.14678 | -2.23082 |

**Single point energy: -1812.12748942 Hartrees**
**Gibbs free energy: -1811.875670 Hartrees**
**No. img. Freqs= 1**

| | | | |
|---|---|---|---|
| C | -2.16591 | -0.08384 | -2.30021 |
| C | -0.88492 | 0.24699 | -1.79658 |
| C | -0.52827 | -0.02356 | -0.42838 |
| C | -1.50697 | -0.66327 | 0.39293 |
| C | -2.73456 | -1.17428 | -0.21301 |
| C | -3.00346 | -0.94961 | -1.52651 |
| C | 0.72553 | 0.27177 | 0.18694 |
| C | -1.27195 | -0.81987 | 1.77252 |
| H | -3.89738 | -1.33093 | -2.00444 |
| C | -0.10106 | -0.39412 | 2.36553 |
| C | 0.91095 | 0.12439 | 1.55701 |
| H | -2.03383 | -1.27781 | 2.39643 |
| H | 0.05062 | -0.50296 | 3.43485 |
| H | 1.87427 | 0.38207 | 1.98822 |
| C | 1.98534 | 0.61362 | -0.52555 |
| O | 2.80267 | 1.41073 | -0.20090 |
| N | -0.17877 | 1.16538 | -2.59880 |
| O | 0.63454 | 1.94423 | -2.06007 |
| S | -3.80606 | -2.12071 | 0.83186 |
| C | -5.21884 | -2.48223 | -0.23456 |
| H | -5.92531 | -3.02525 | 0.40032 |
| H | -5.69398 | -1.56316 | -0.59018 |
| H | -4.93556 | -3.12276 | -1.07481 |
| O | -0.42850 | 1.25119 | -3.79926 |
| Cl | 2.40855 | -0.56528 | -1.85074 |
| H | -4.79184 | 4.12327 | -1.97532 |
| H | -3.86705 | 2.70223 | -2.53154 |
| C | -2.31593 | 4.88760 | -2.86885 |
| H | -3.09741 | 5.58853 | -3.17818 |
| H | -1.38211 | 5.42885 | -2.69884 |
| H | -2.15958 | 4.14400 | -3.65540 |
| H | -2.77291 | 4.86673 | -0.87133 |

**Single point energy: -1812.12346322 Hartrees**
**Gibbs free energy: -1811.873003 Hartrees**
**No. img. Freqs= 1**

| | | | |
|---|---|---|---|
| C | -0.67386 | 1.05627 | -1.65038 |
| C | 0.51006 | 0.62182 | -1.00863 |
| C | 0.49686 | -0.47444 | -0.08209 |
| C | -0.76022 | -1.10658 | 0.17686 |
| C | -1.91198 | -0.77914 | -0.65790 |
| C | -1.82985 | 0.20533 | -1.59088 |
| C | 1.62844 | -1.01648 | 0.60277 |
| C | -0.86920 | -2.06128 | 1.20698 |
| H | -2.65722 | 0.44314 | -2.24921 |
| C | 0.21884 | -2.44470 | 1.96371 |
| C | 1.47788 | -1.94402 | 1.62628 |
| H | -1.83633 | -2.50644 | 1.42270 |
| H | 0.11016 | -3.16433 | 2.76918 |
| H | 2.36400 | -2.30954 | 2.13796 |
| C | 3.05160 | -0.80053 | 0.23334 |
| O | 3.97722 | -0.66006 | 0.96240 |
| N | 1.60011 | 1.51879 | -1.12088 |
| O | 2.50166 | 1.48841 | -0.27039 |
| S | -3.37420 | -1.75421 | -0.42245 |
| C | -4.57086 | -0.98846 | -1.53876 |
| H | -5.50387 | -1.53735 | -1.38003 |
| H | -4.73187 | 0.06498 | -1.29027 |
| H | -4.27272 | -1.09692 | -2.58554 |
| O | 1.61678 | 2.34357 | -2.03279 |
| Cl | 3.39029 | -1.14017 | -1.53189 |
| H | -0.56334 | 1.69626 | -2.51555 |
| O | -1.21043 | 2.57112 | -0.58458 |
| H | -1.28546 | 2.49632 | 0.82793 |
| H | -1.89404 | 3.06414 | -1.05068 |
| N | -1.27743 | 2.44630 | 1.95054 |
| C | 0.07739 | 2.08703 | 2.41763 |
| H | 0.79627 | 2.78884 | 1.99058 |
| H | 0.11995 | 2.12231 | 3.50994 |
| H | 0.31128 | 1.07729 | 2.07405 |
| C | -2.32938 | 1.51889 | 2.40788 |
| H | -2.39235 | 1.52496 | 3.49964 |
| H | -3.28530 | 1.82658 | 1.97778 |
| H | -2.08254 | 0.51293 | 2.06079 |
| H | -1.49720 | 3.38764 | 2.27573 |

## 5 NMR Spectra

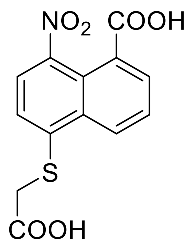


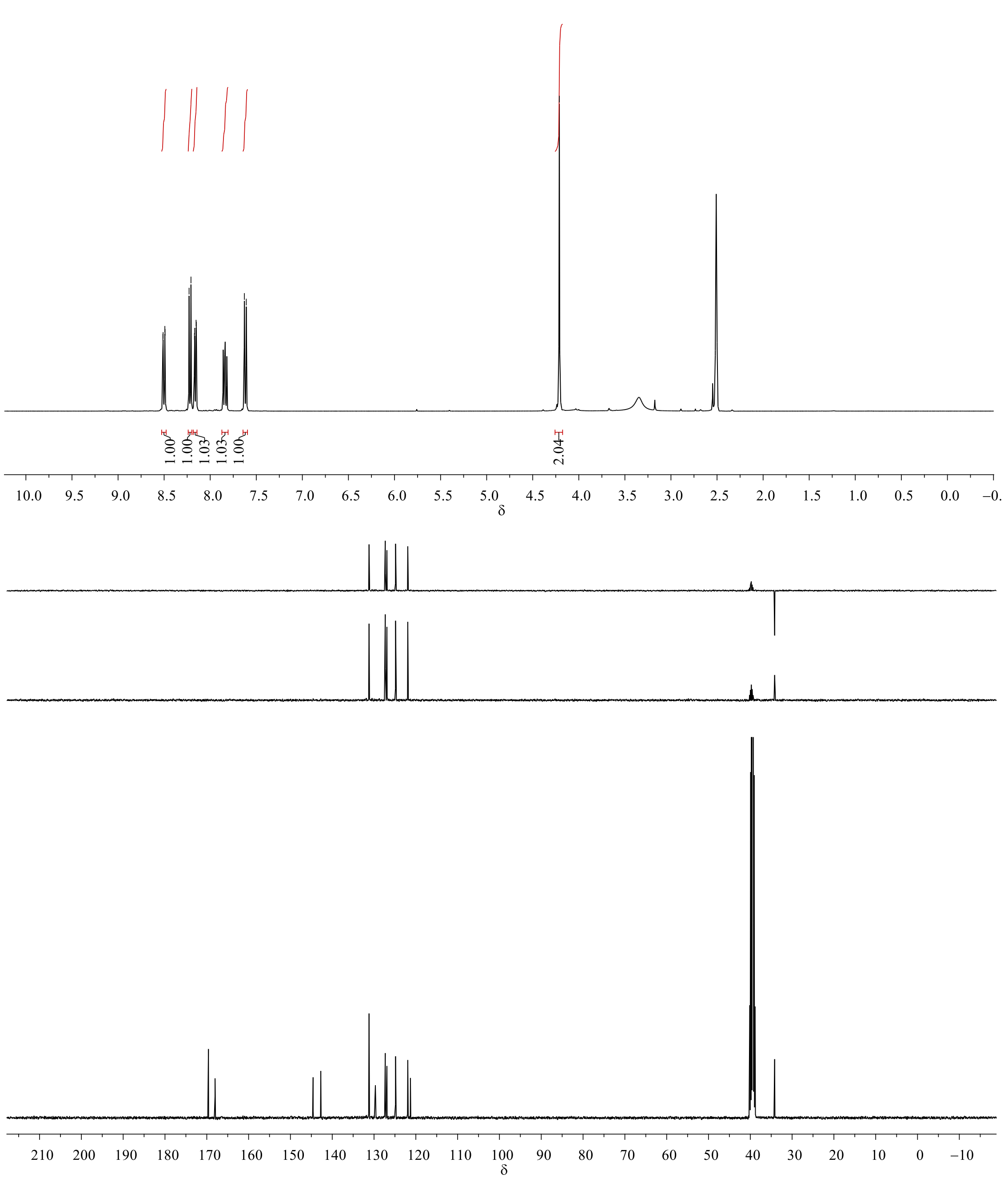


Figure S2. $^{1}H$ (400 MHz) and $^{13}C\{^{1}H\}$ (101 MHz) spectra of 5-((carboxymethyl)thio)-8-nitro-1-naphthoic acid in DMSO-$d_6$.

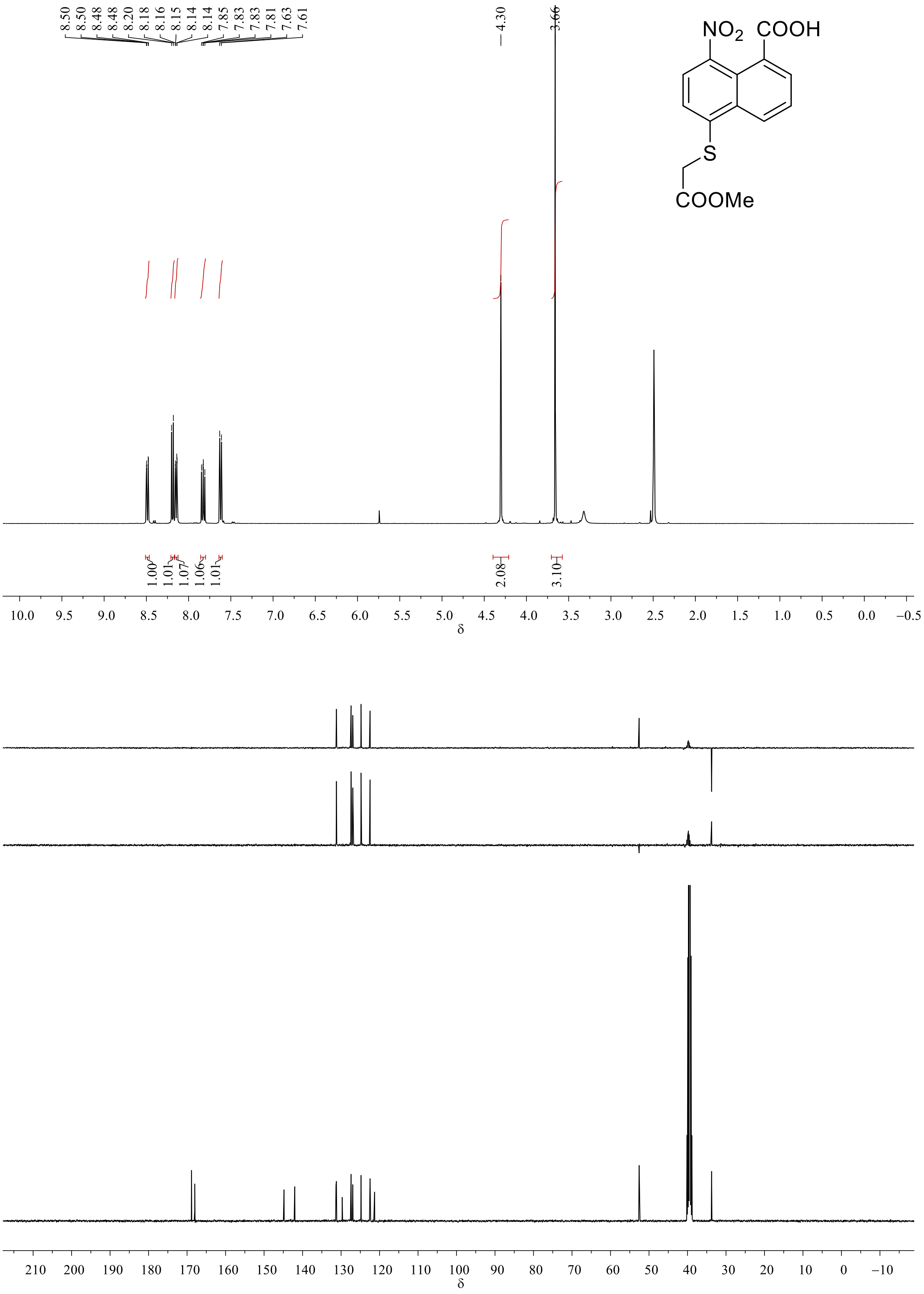


Figure S3. $^{1}H$ (400 MHz) and $^{13}C\{^{1}H\}$ (101 MHz) spectra of compound **1** in DMSO-$d_6$.

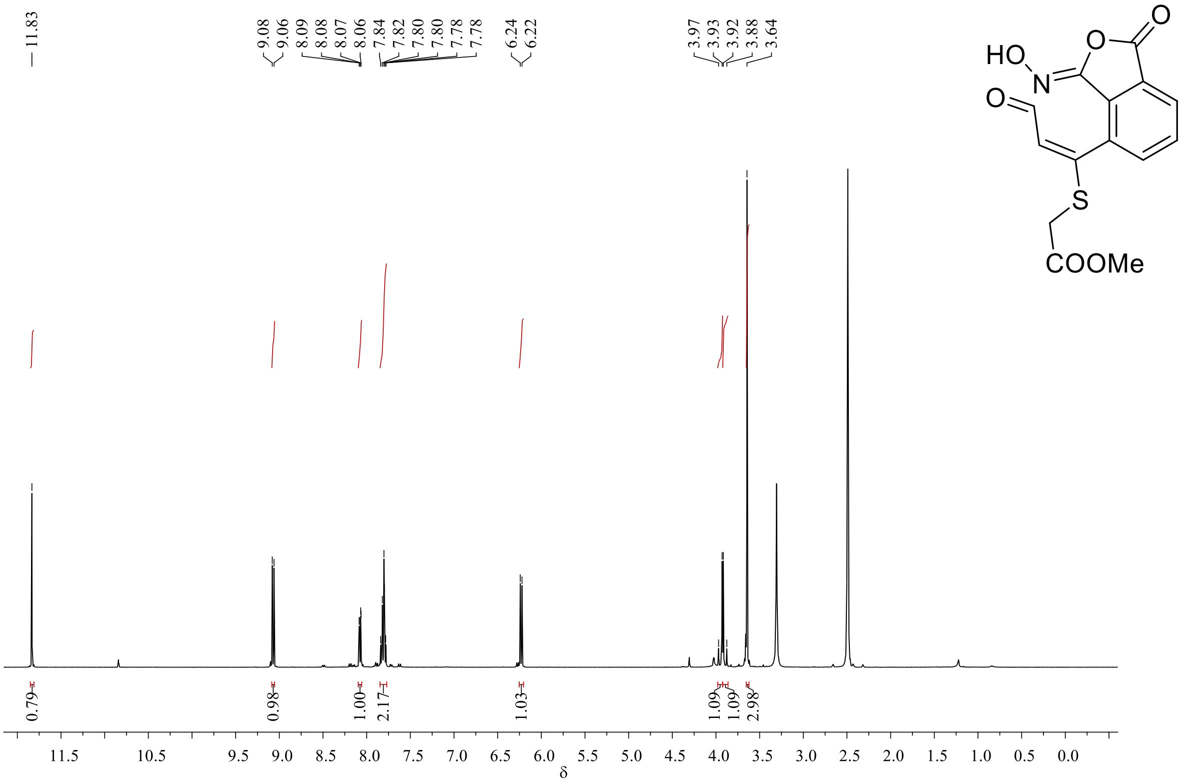

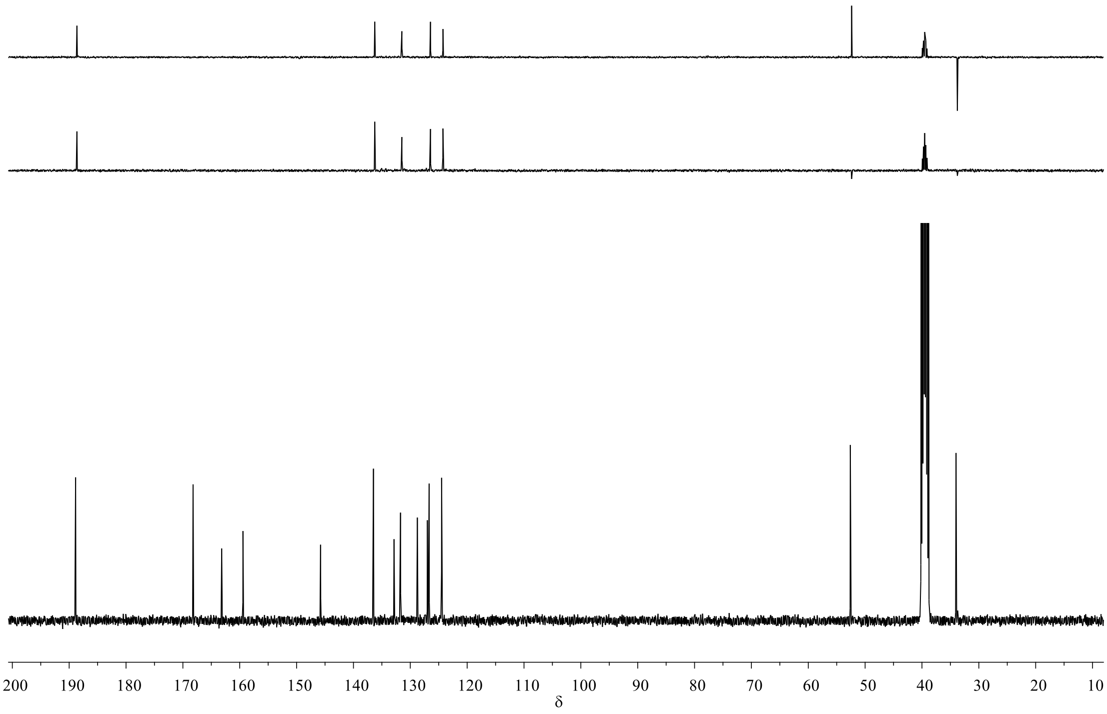

Figure S4. $^{1}H$ (400 MHz) and $^{13}C\{^{1}H\}$ (101 MHz) spectra of compound **4** in DMSO-$d_6$.

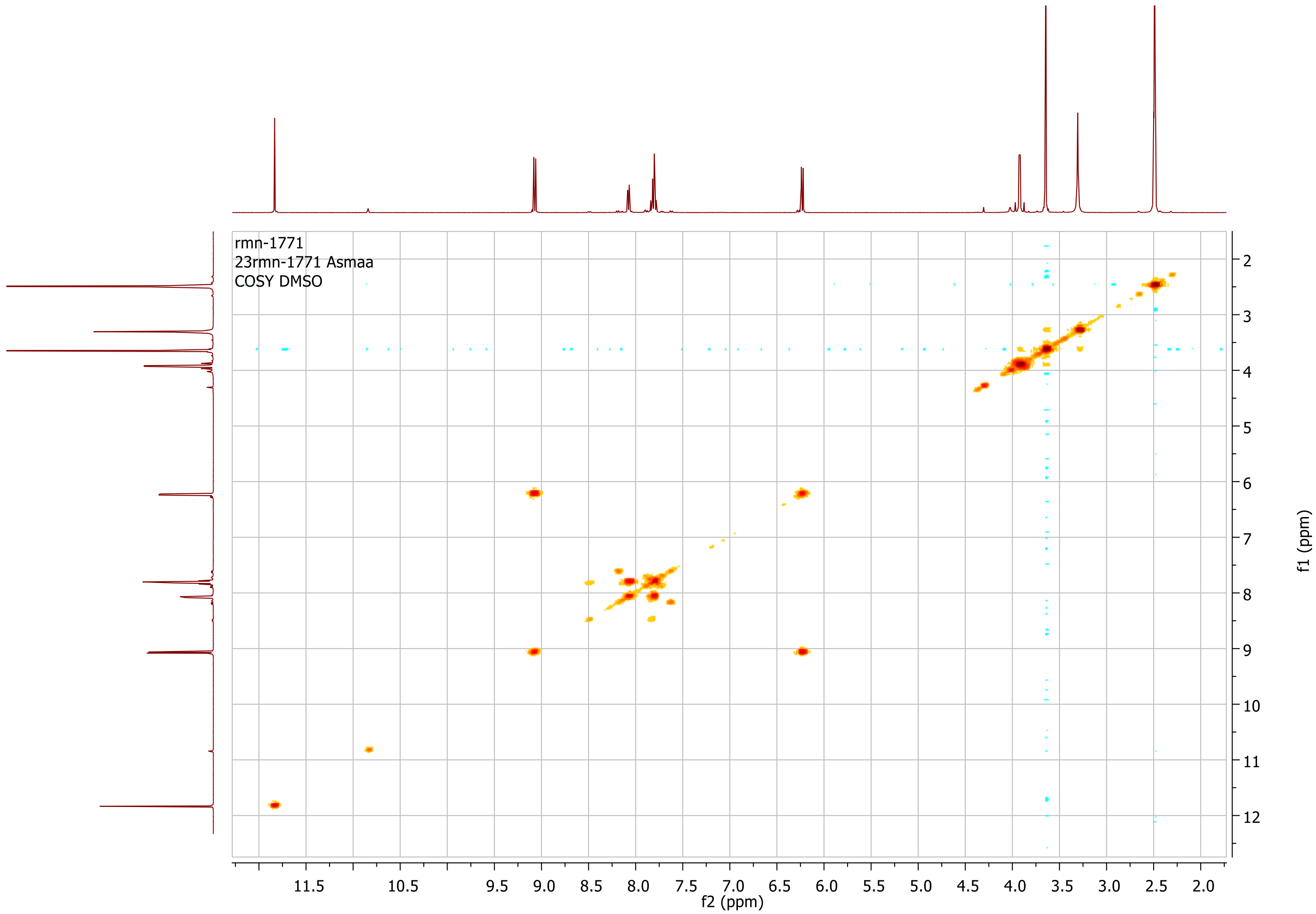


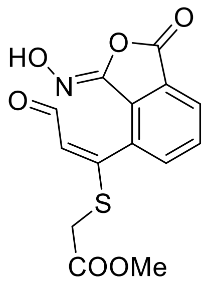


Figure S5. COSY spectrum of compound **4** in DMSO-$d_6$.

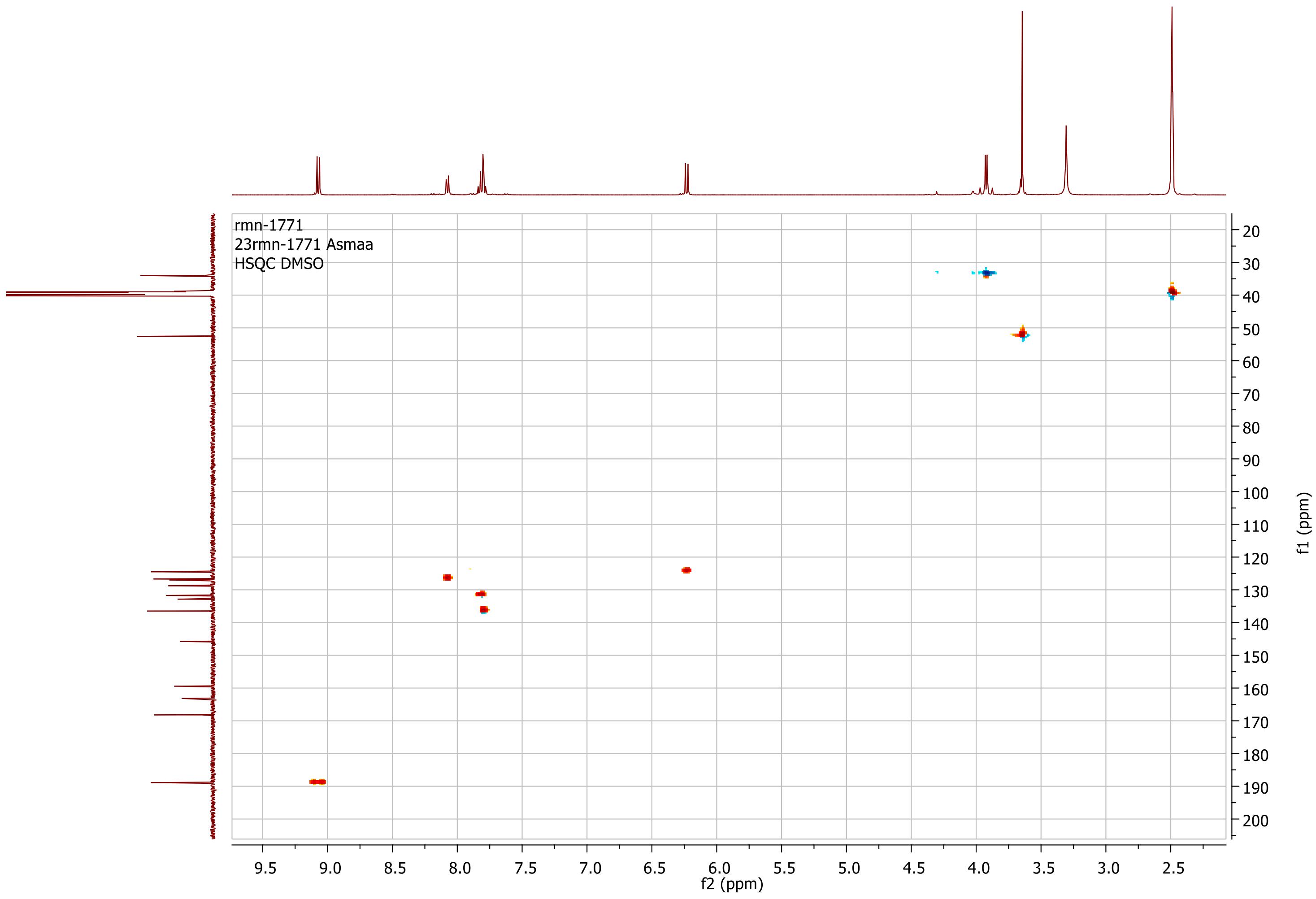


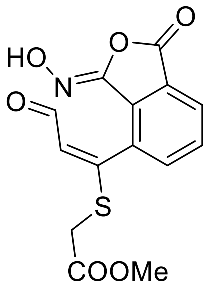


Figure S6. HSQC 145 Hz spectrum of compound **4** in DMSO-$d_6$.

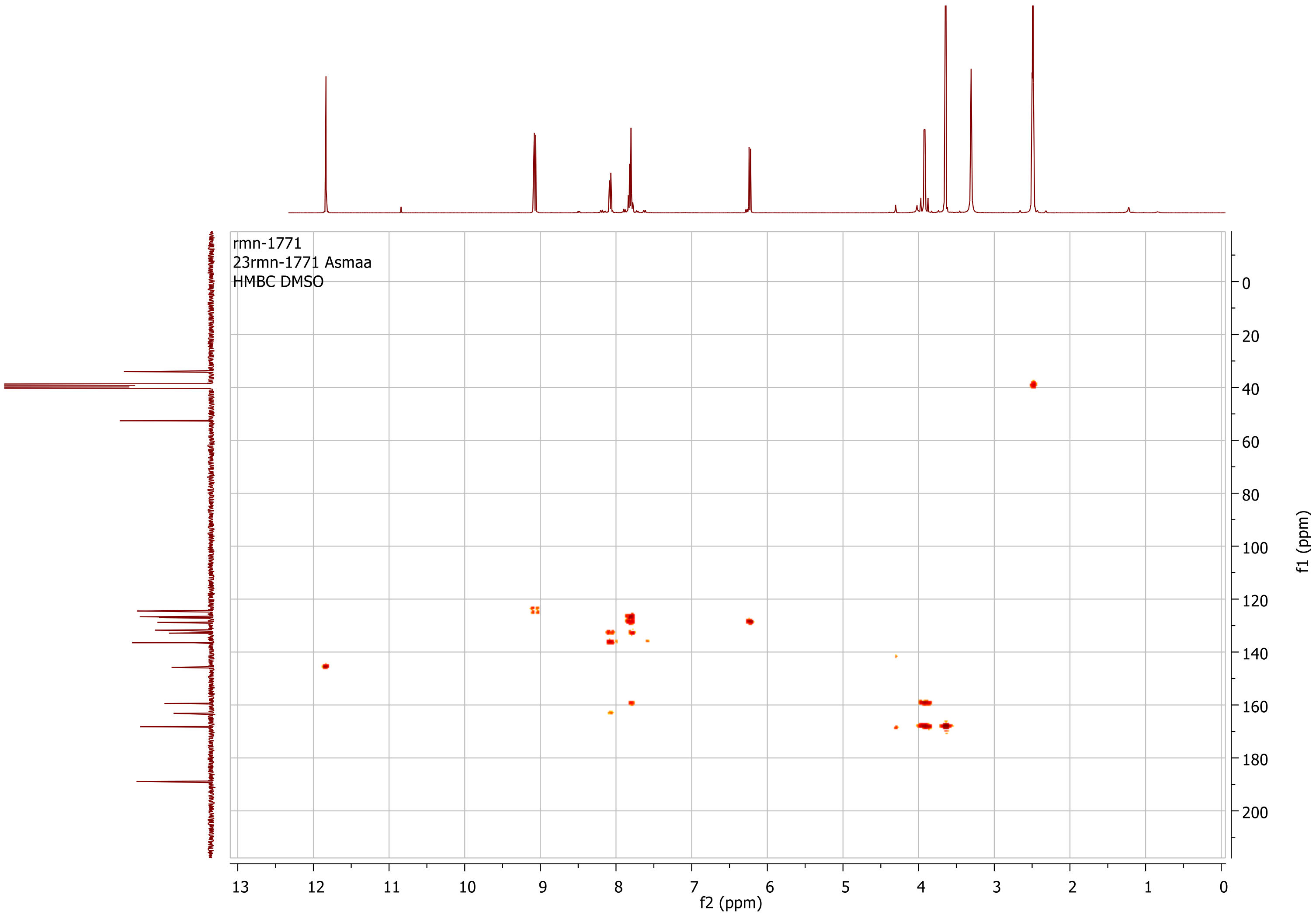


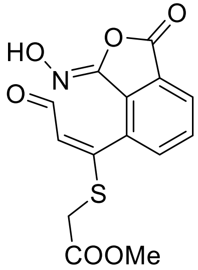


Figure S7. HMBC CIGAR spectrum of compound **4** in DMSO-$d_6$.

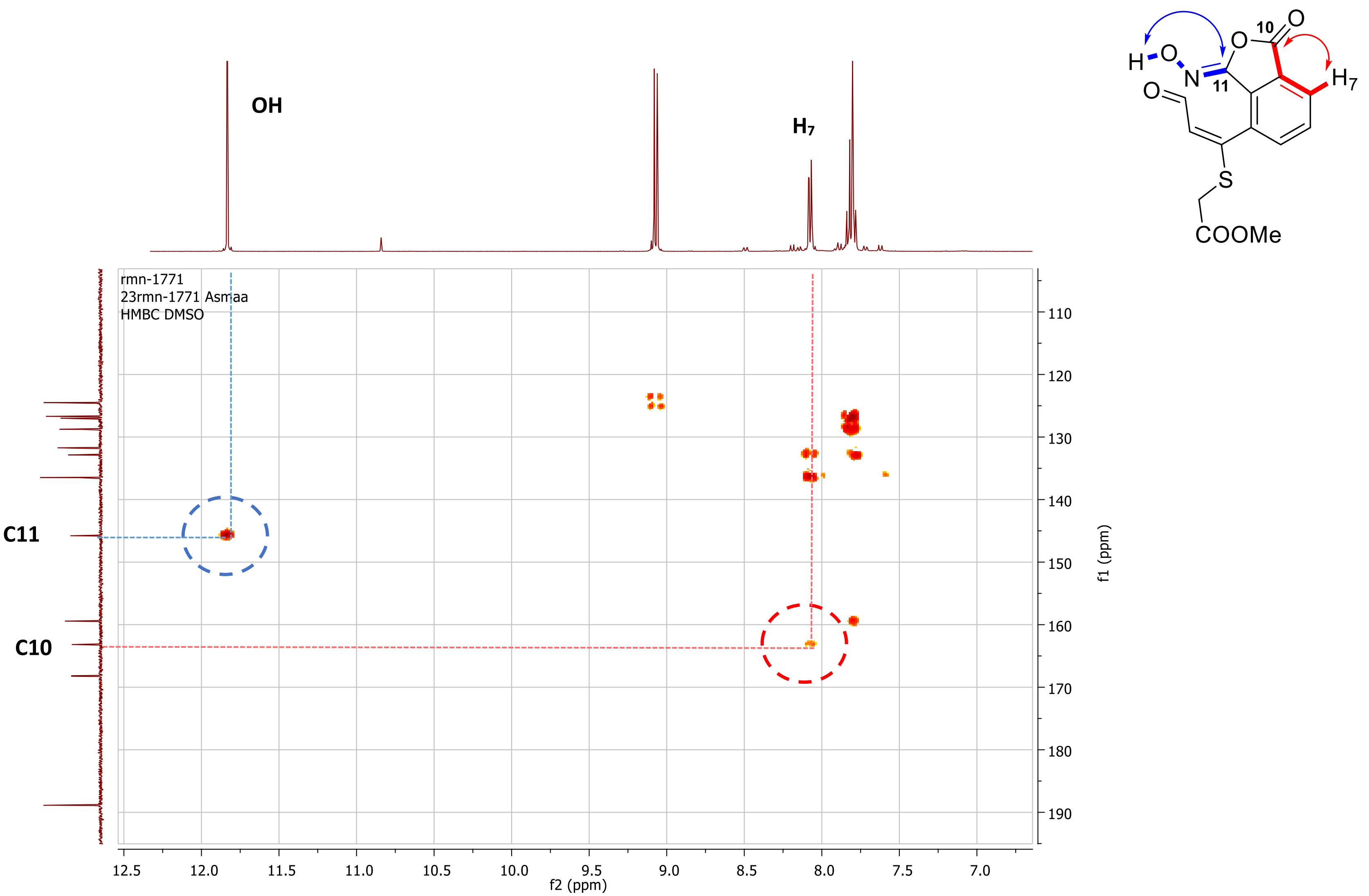


Figure S8. Expansion of HMBC CIGAR spectrum of compound **4** in DMSO-$d_6$.

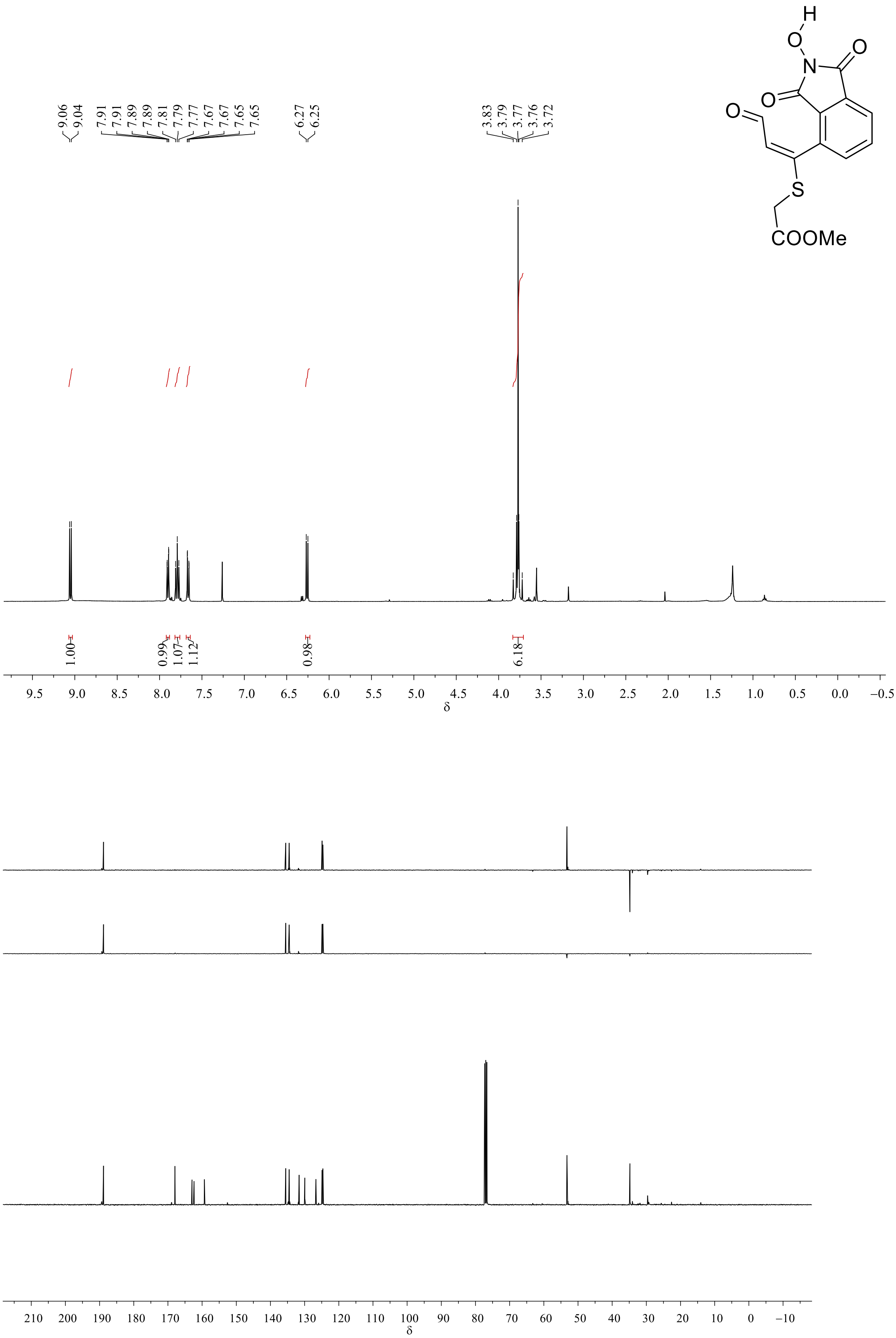


Figure S9. $^{1}$H (400 MHz) and $^{13}$C{$^{1}$H} (101 MHz) spectra of compound **5** in $CDCl_3$.

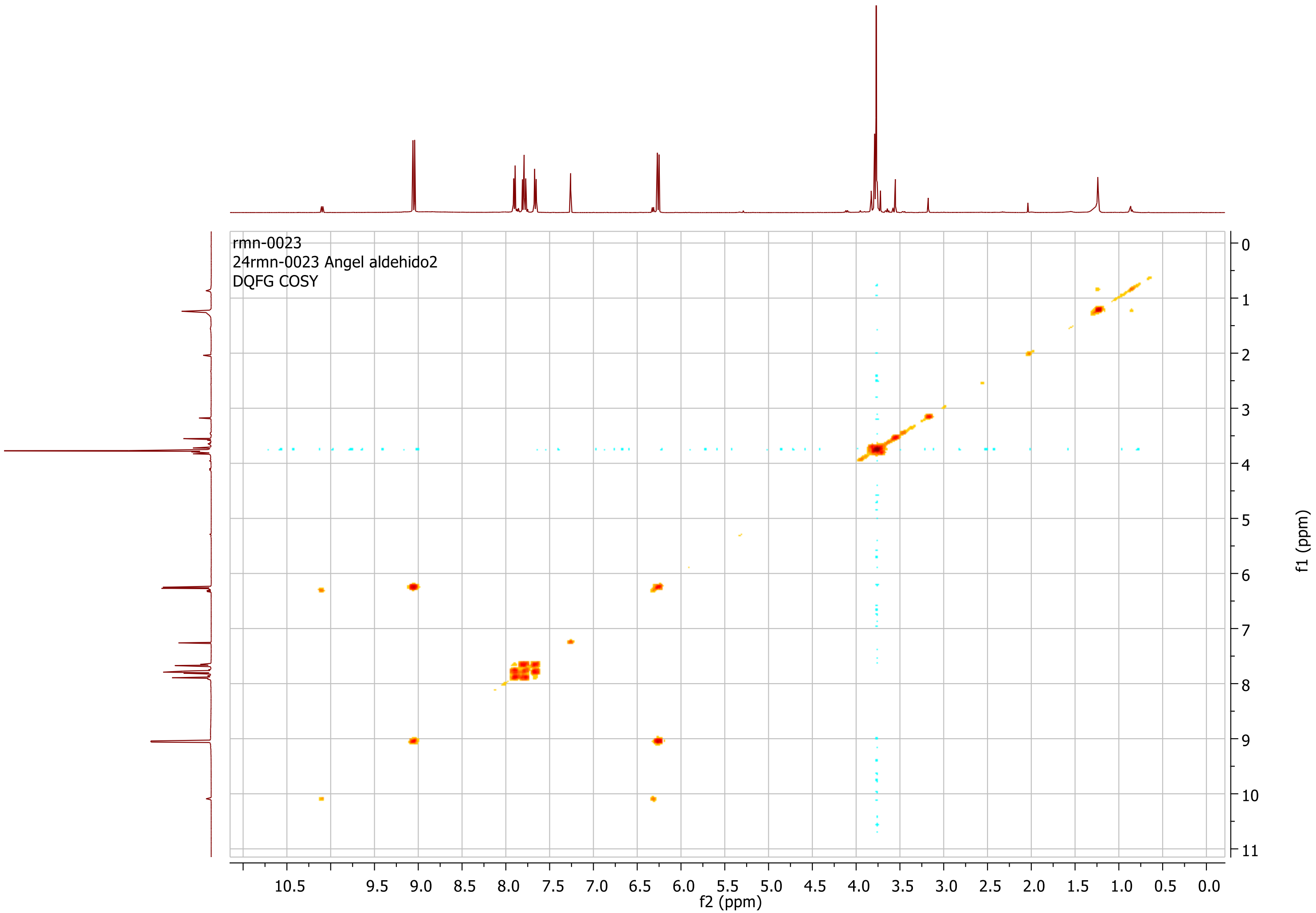


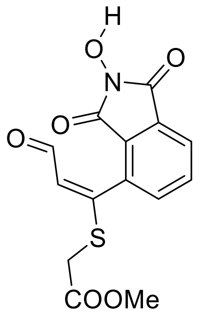

Figure S10. COSY spectrum of compound **5** in $CDCl_3$.

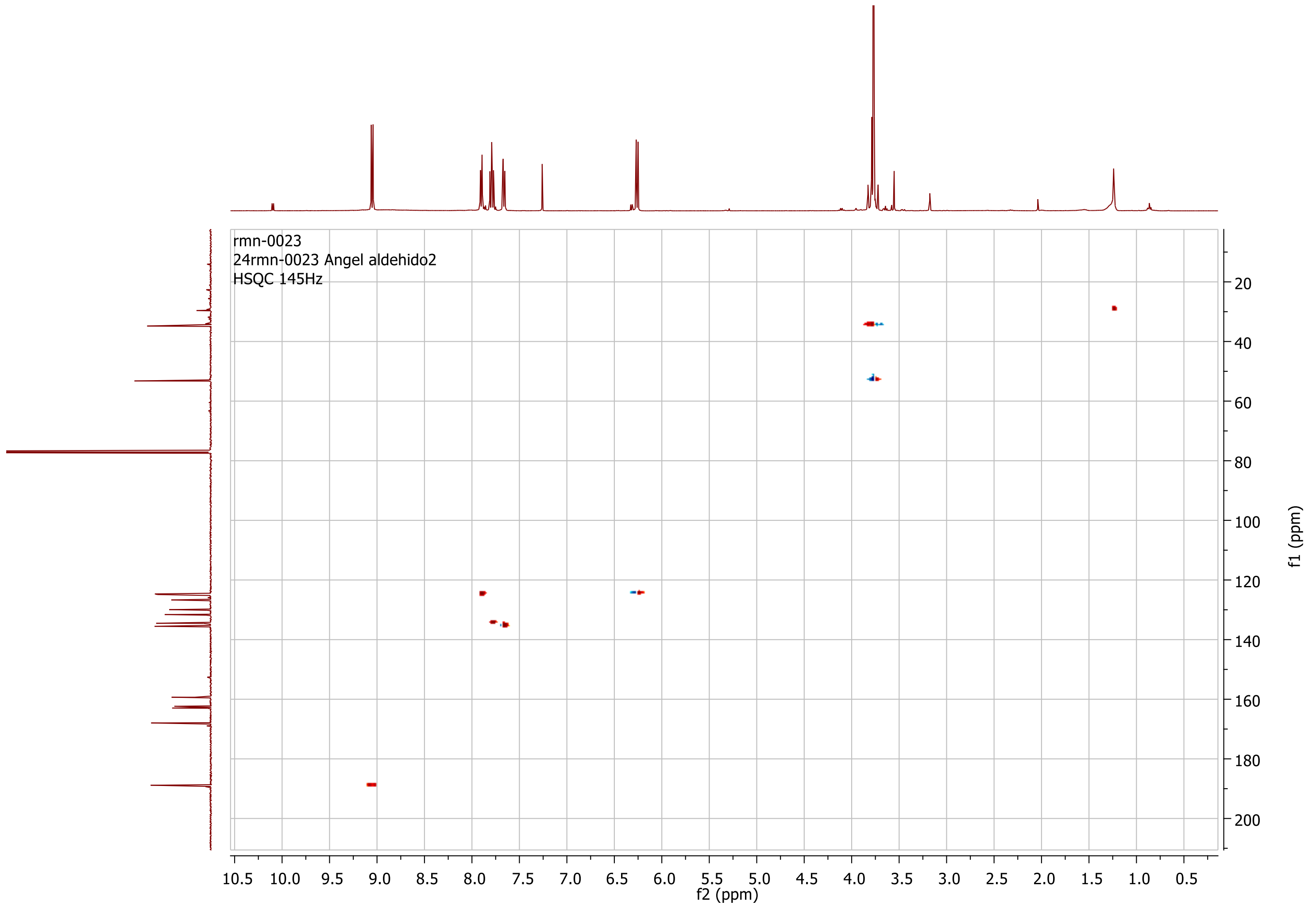


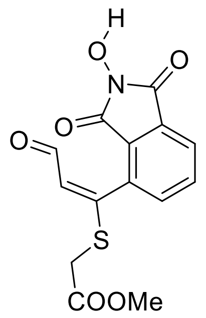


Figure S11. HSQC 145 Hz spectrum of compound **5** in $CDCl_3$.

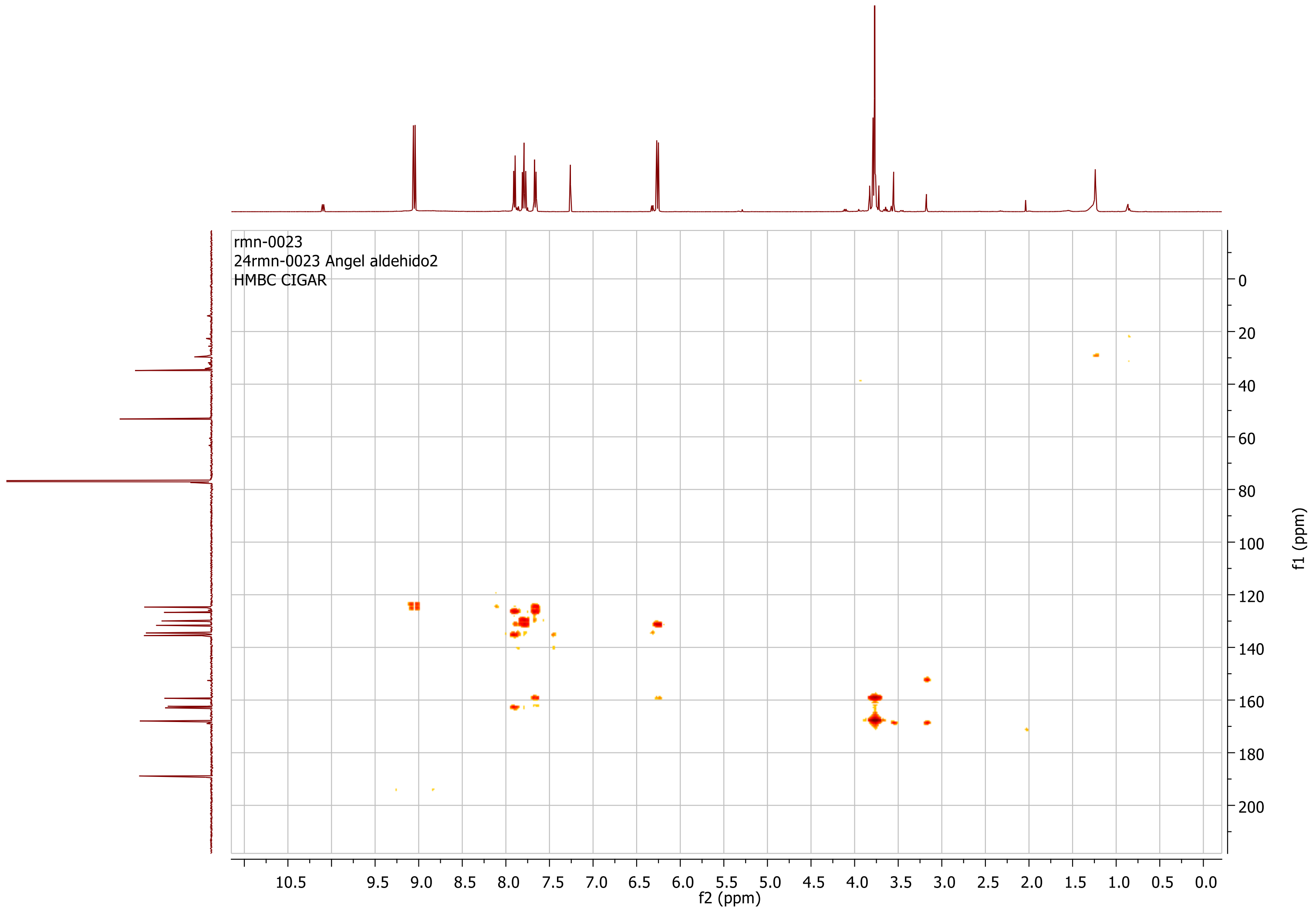


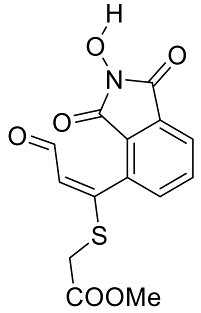


Figure S12. HMBC CIGAR spectrum of compound **5** in $CDCl_3$.

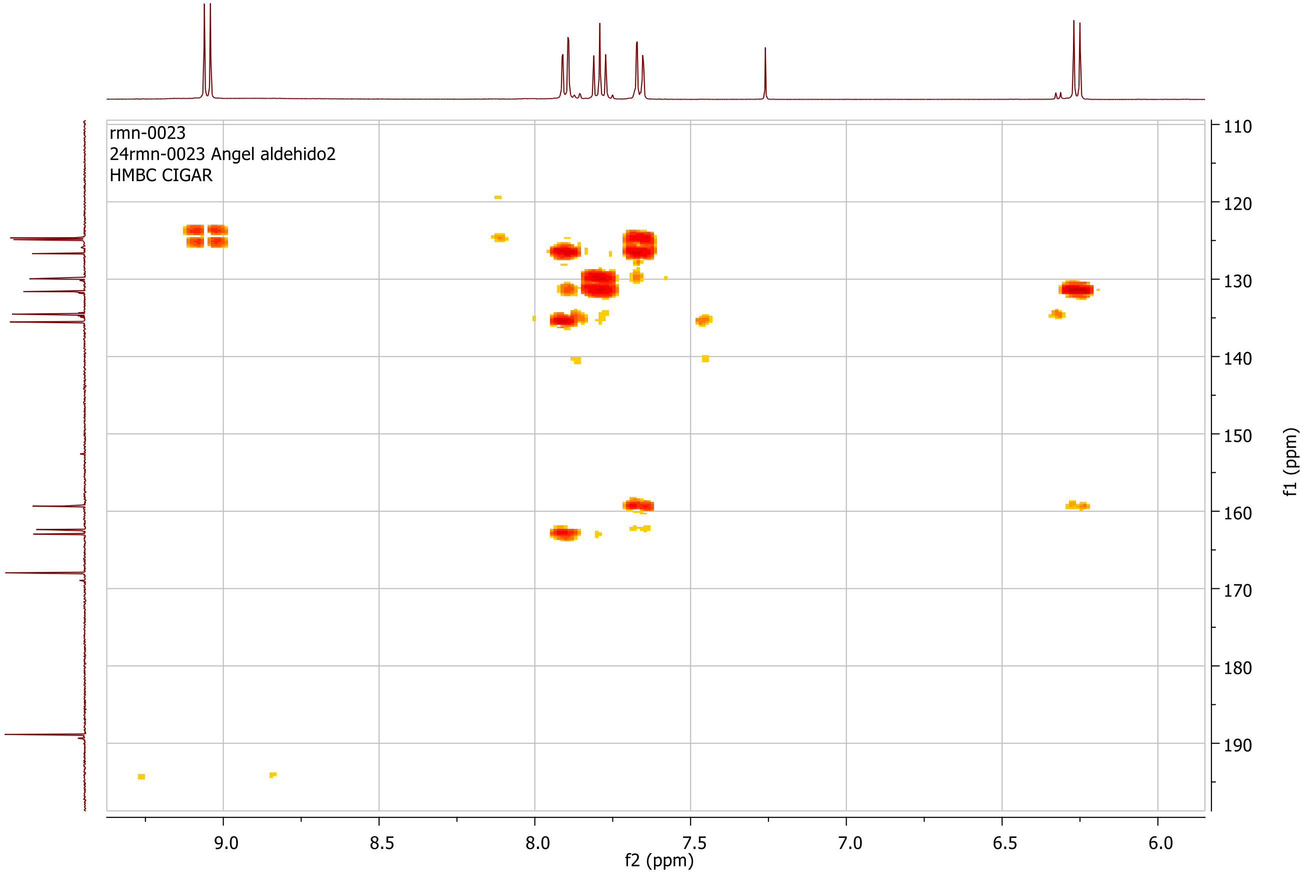


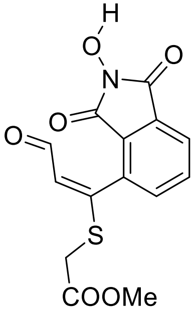


Figure S13. Expansion of HMBC CIGAR spectrum of compound **5** in $CDCl_3$.

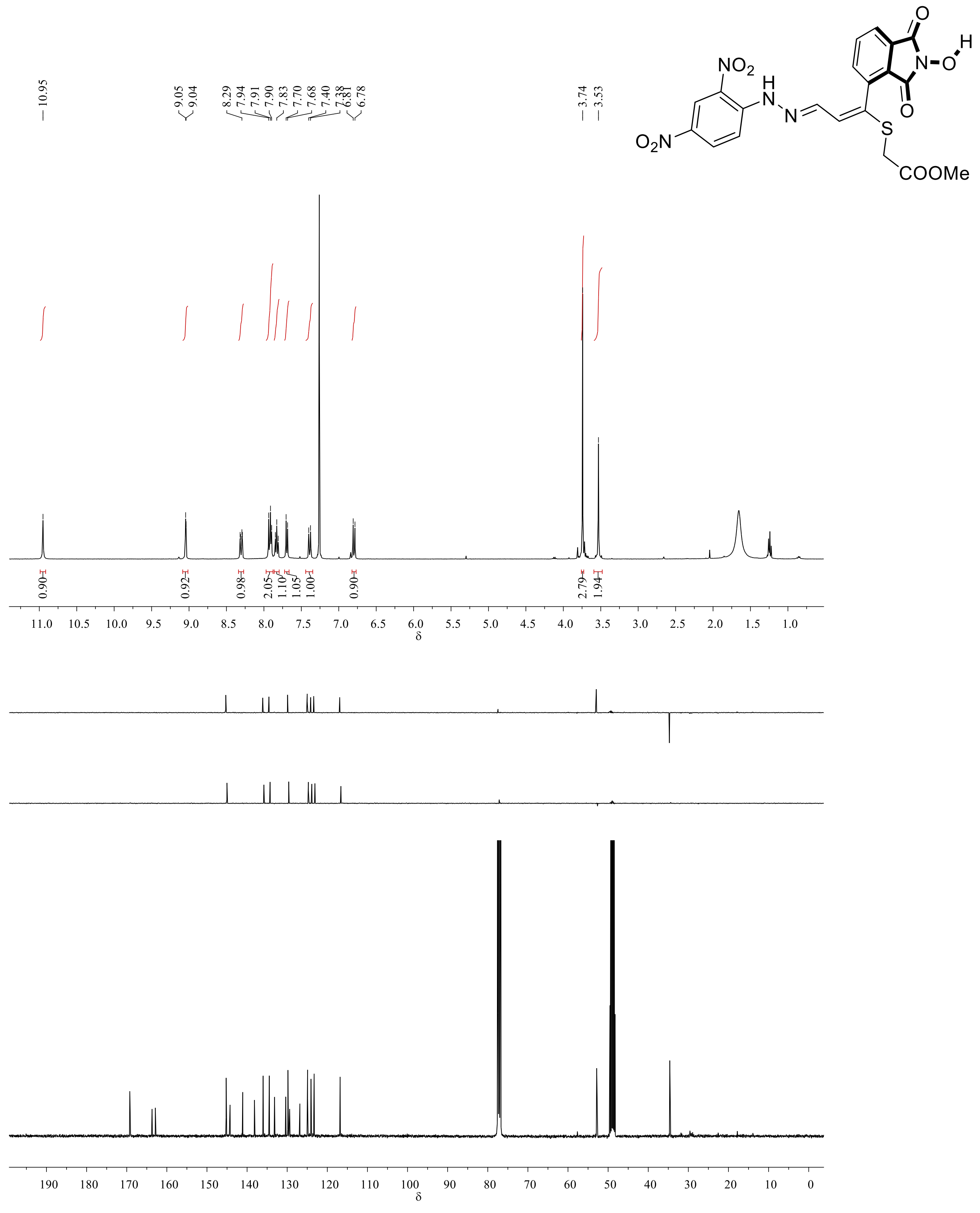

Figure S14. $^{1}$H (400 MHz) spectra of compound **6** in $CDCl_3$ and $^{13}C\{^1H\}$ (101 MHz) spectra in $CDCl_3/CD_3OD$.

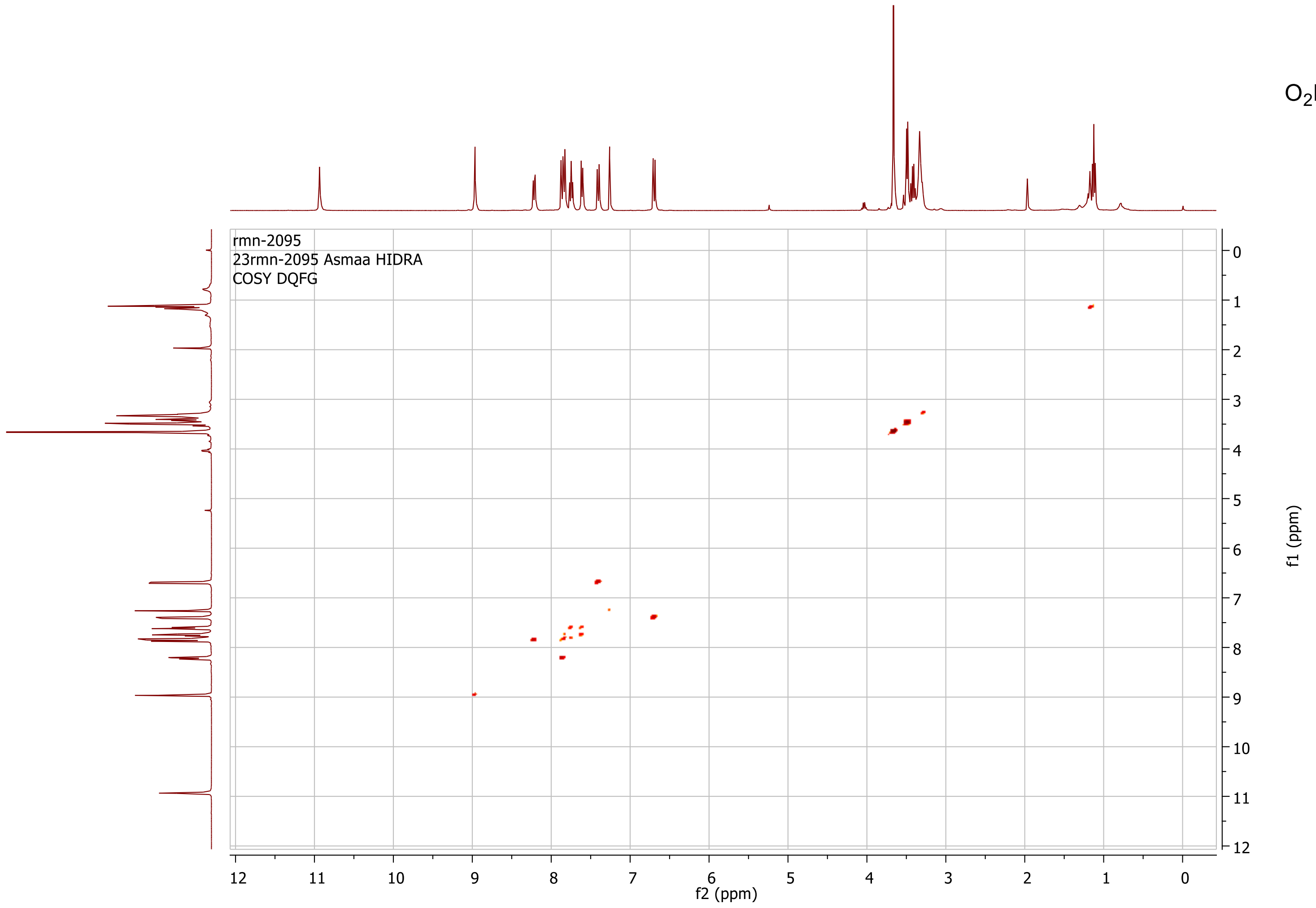


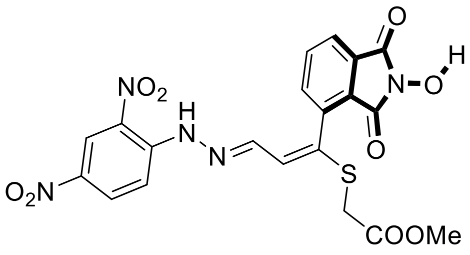


Figure S15. COSY spectrum of compound **6** in $CDCl_3$/$CD_3OD$.

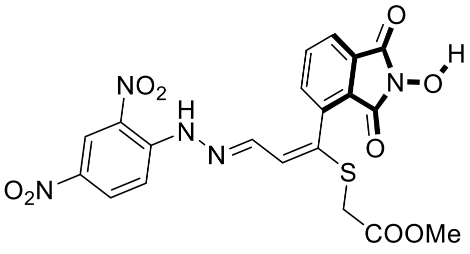


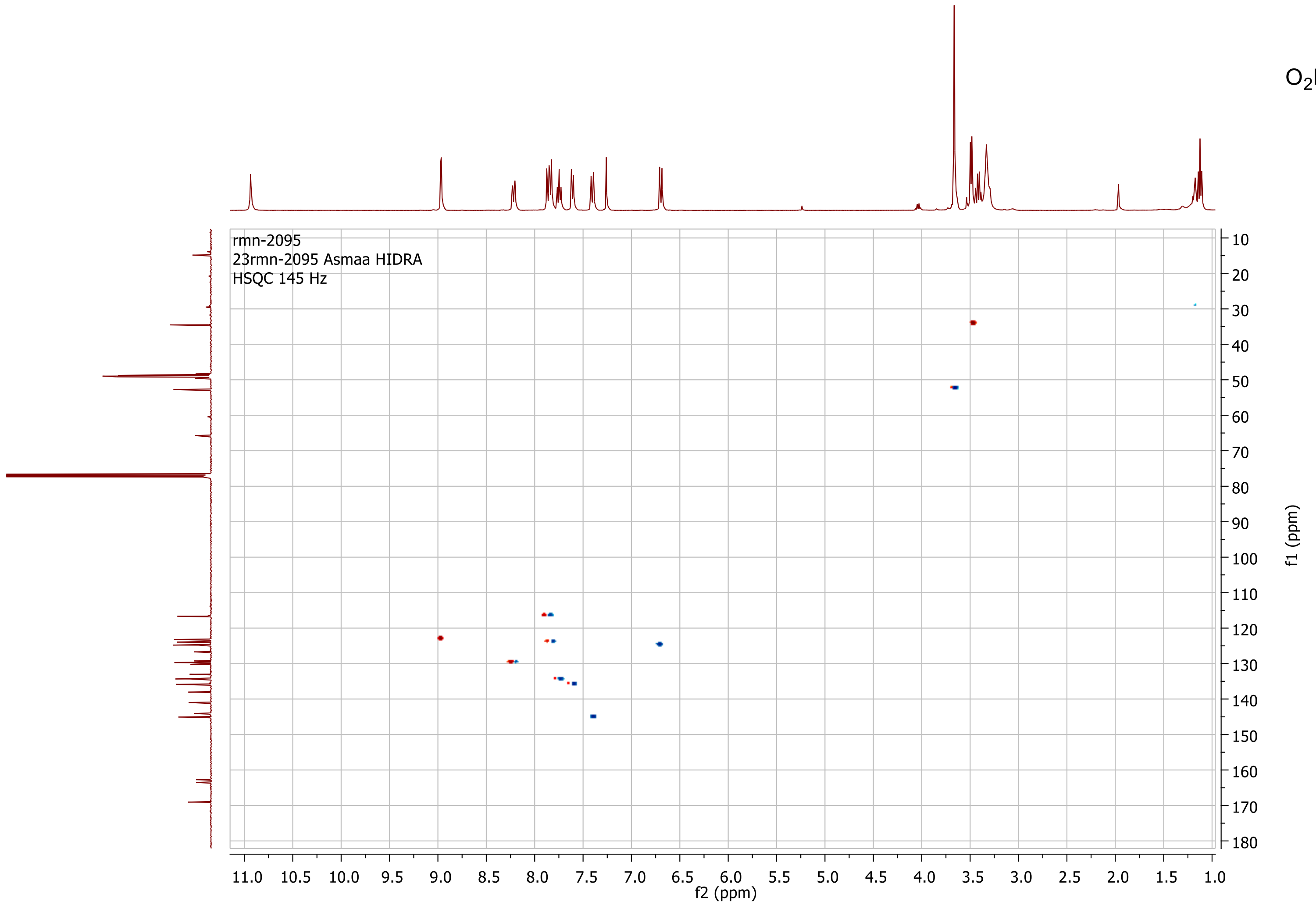


Figure S16. HSQC 145 Hz spectrum of compound **6** in $CDCl_3/CD_3OD$.

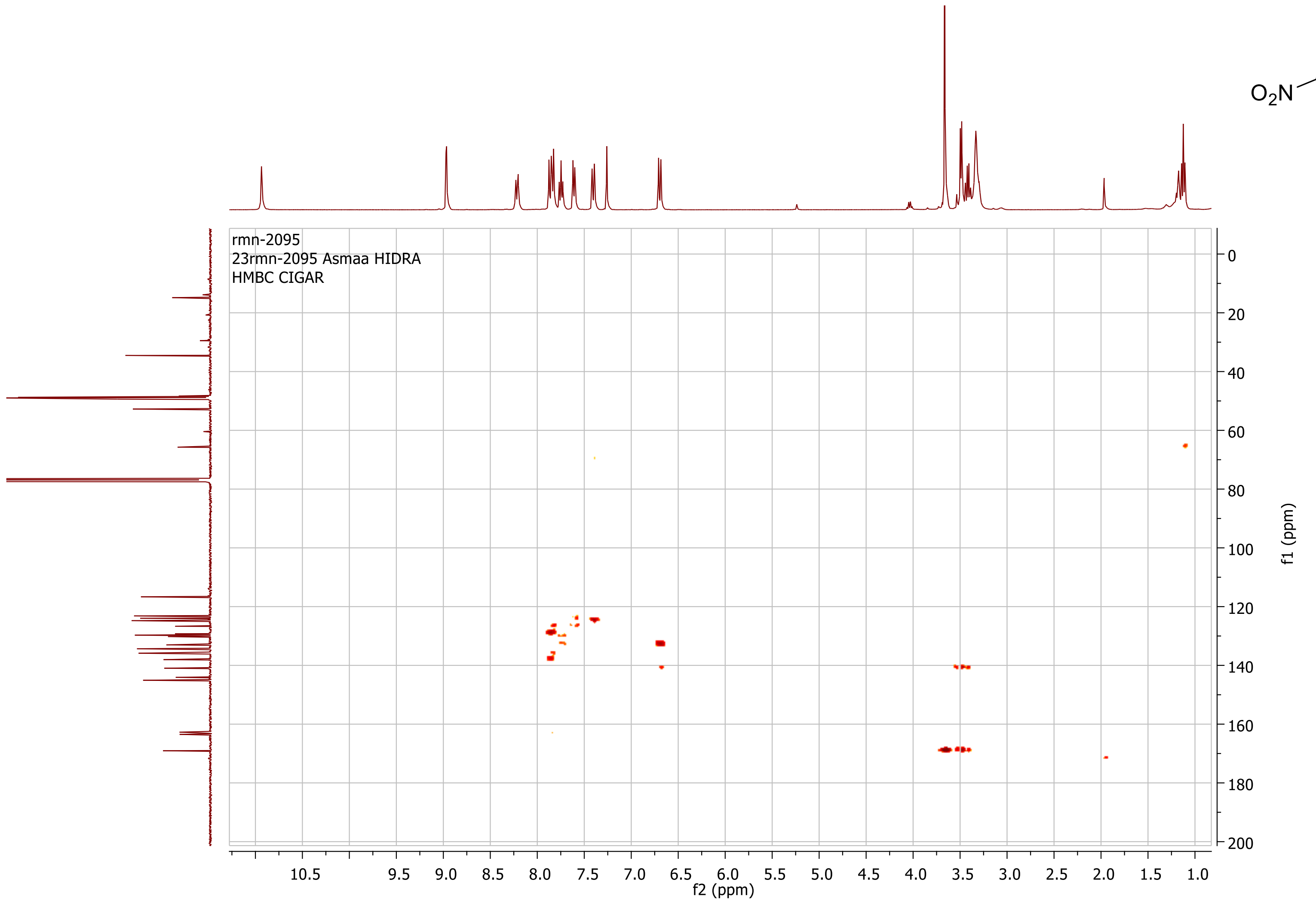


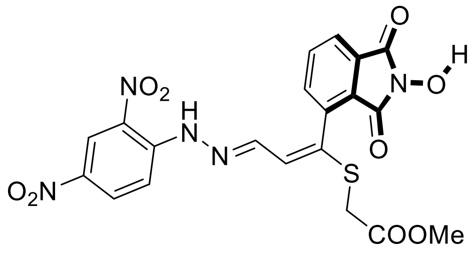


Figure S17. HBMC CIGAR spectrum of compound **6** in $CDCl_3/CD_3OD$.

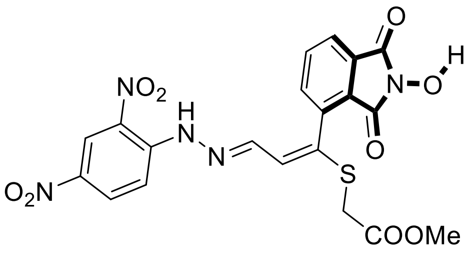


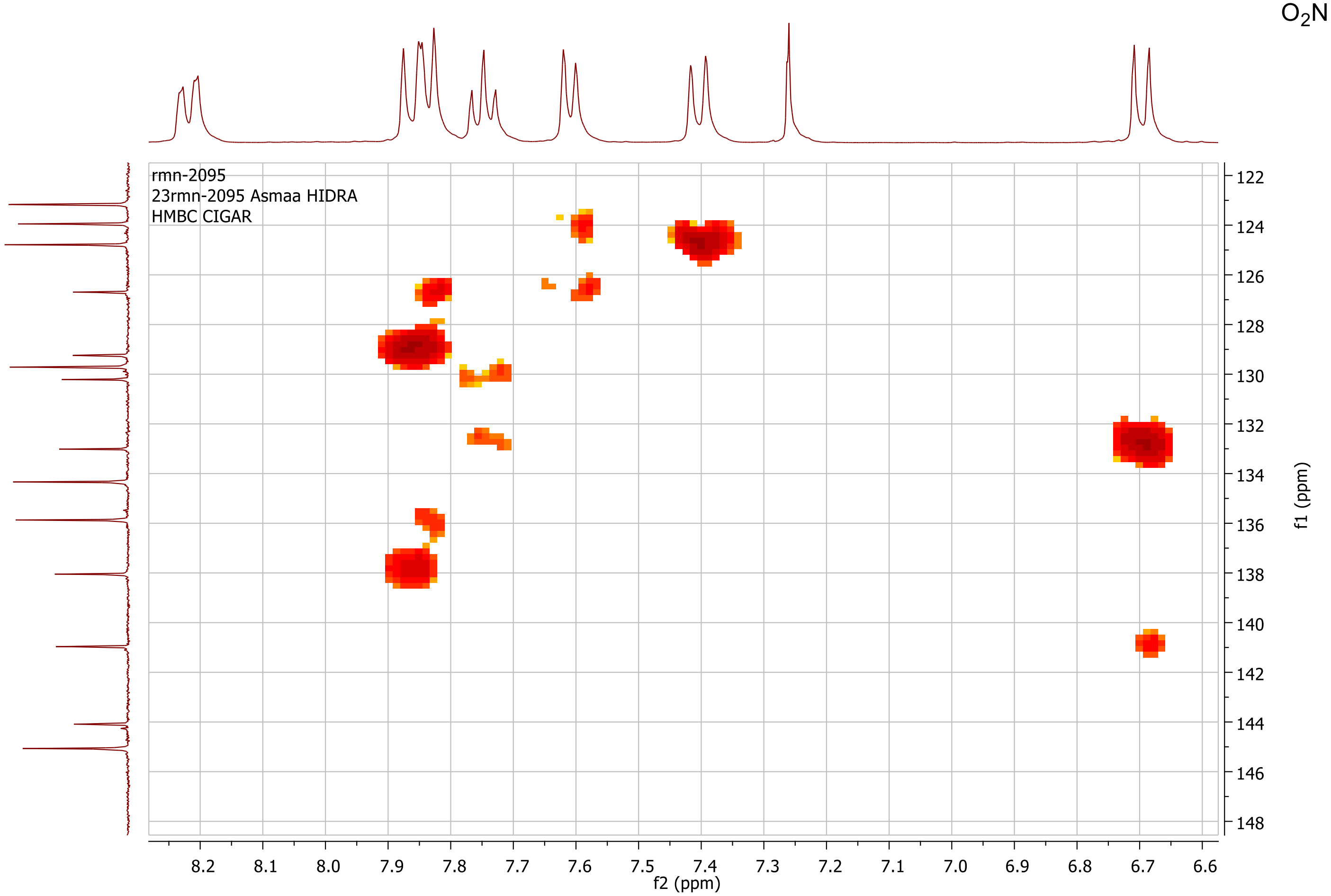


Figure S18. Expansion of HMBC CIGAR spectrum of compound **6** in $CDCl_3/CD_3OD$.

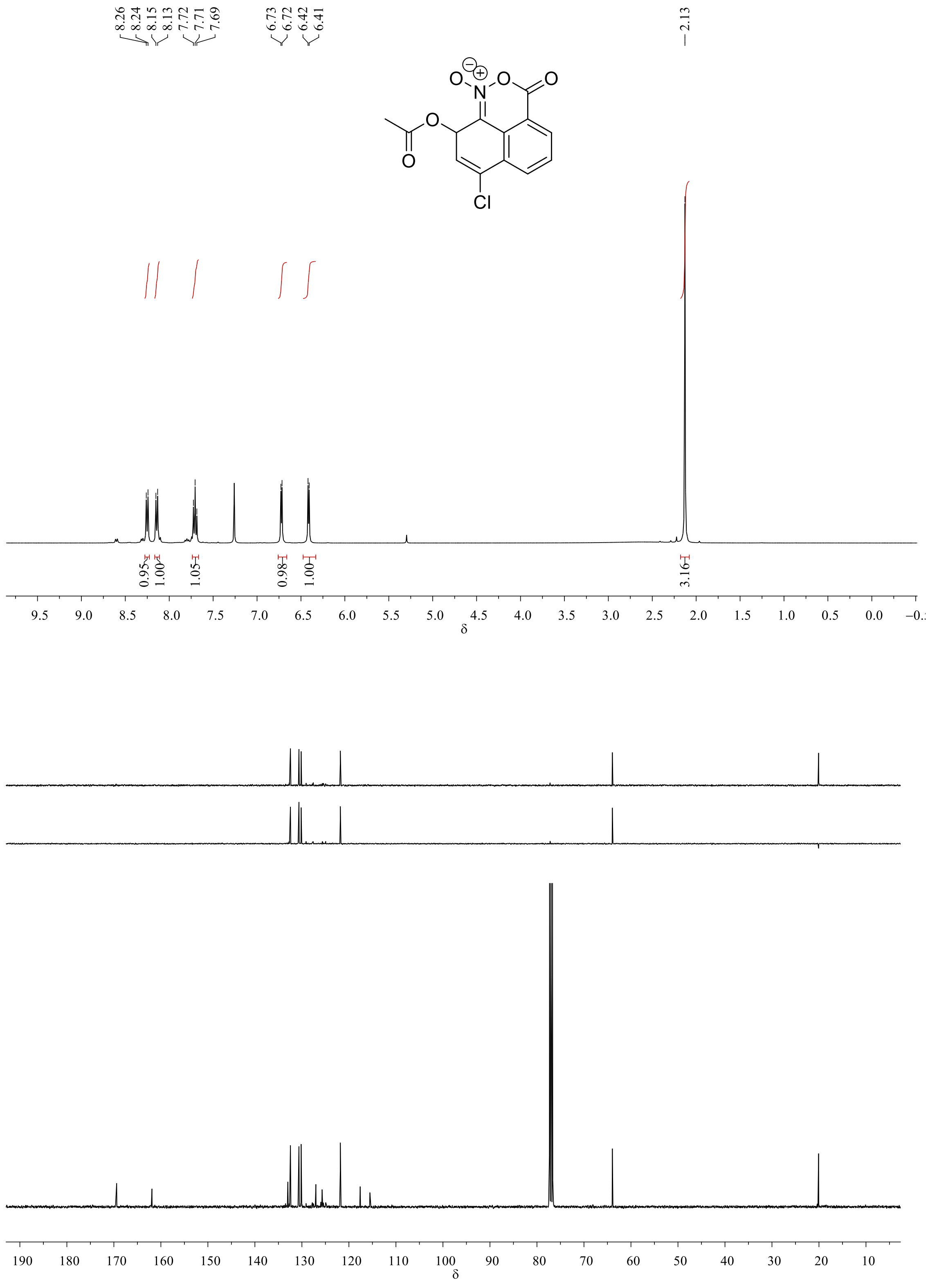

Figure S19. $^{1}H$ (400 MHz) and $^{13}C\{^{1}H\}$ (101 MHz) spectra of compound **12** in $CDCl_3$.

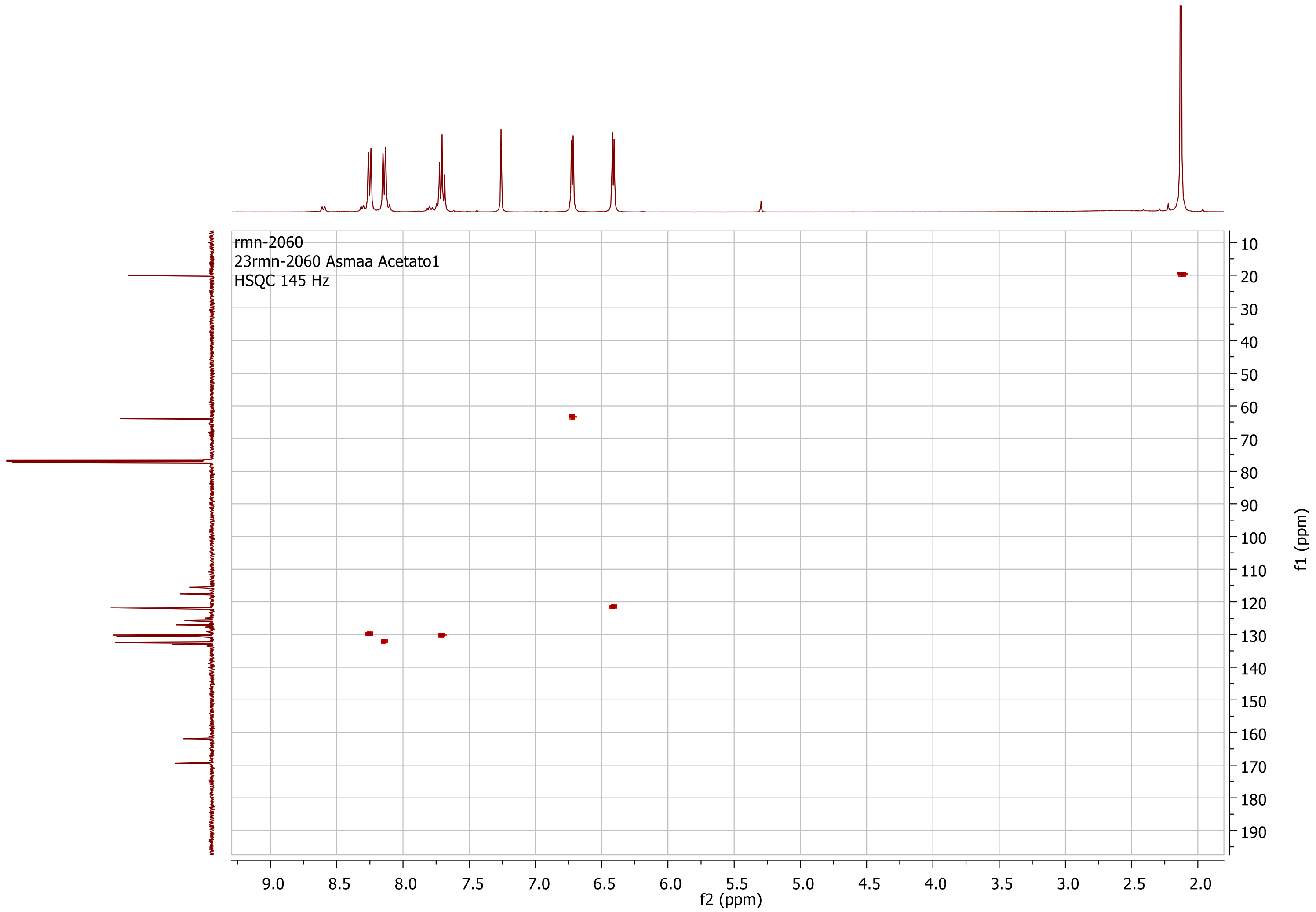


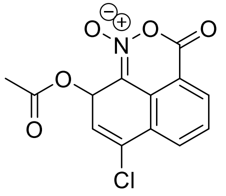

Figure S20. HSQC 145 Hz spectrum of compound **12** in $CDCl_3$.

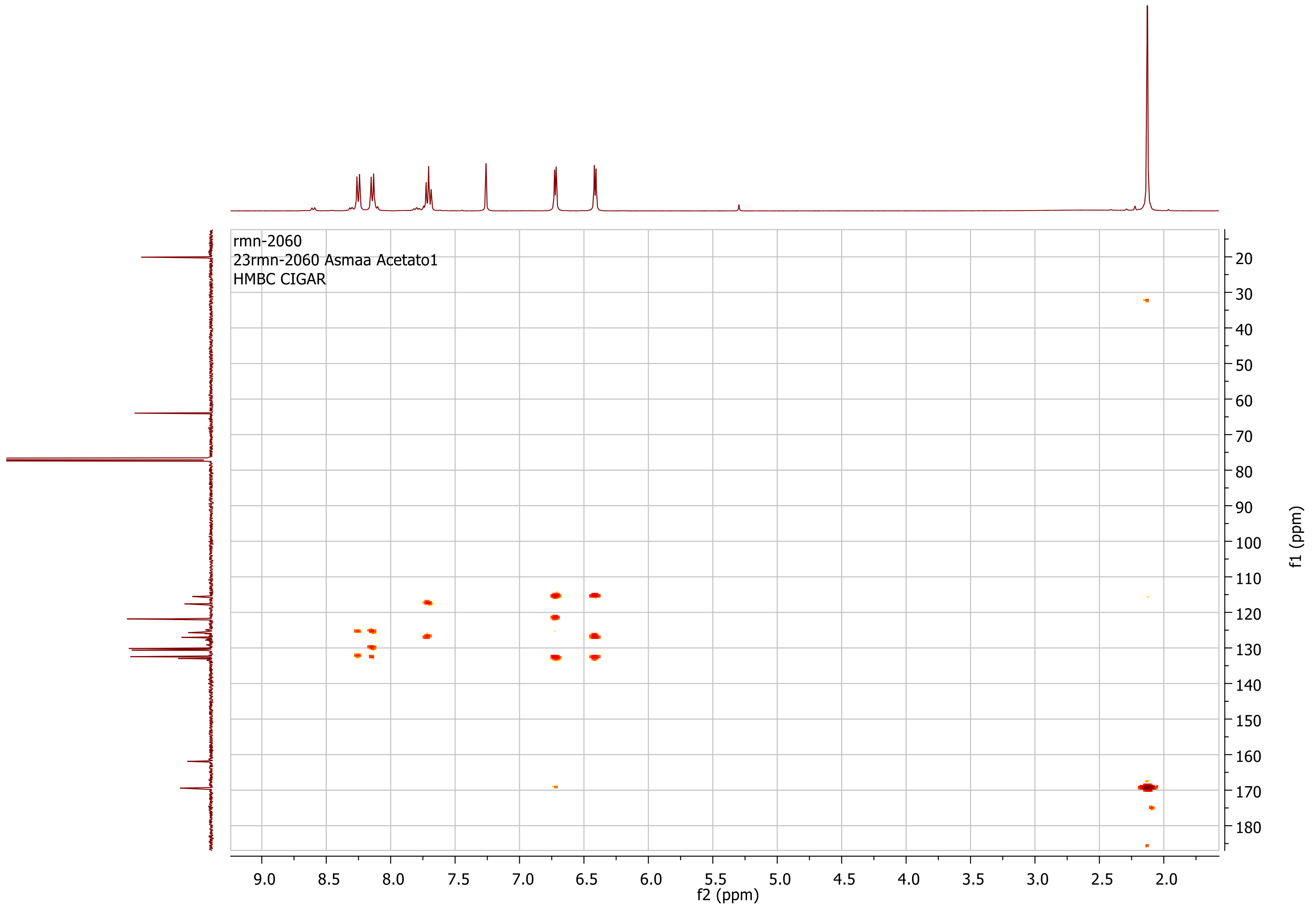


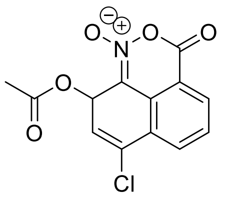


Figure S21. HBMC CIGAR spectrum of compound **12** in $CDCl_3$.

## 6 References


[1] Armarego, W. L. F.; Perrin, D. D. Purification of Laboratory Chemicals; 3th ed.; Pergamon Press: Oxford, 1988.

[2] Gottlieb, H. E.; Kotlyar, V.; Nudelman, A. *J. Org. Chem.* **1997**, *62*, 7512-7515.

[3] Habib, A.; Garrido-González, J. J.; Sánchez-Santos, E.; Boya del Teso, I.; Sanz, F.; Alcázar, F.; Fuentes de Arriba, Á. L.; Morán, J. R. 5-Chloro-8-nitro-1-naphthoyl (NNap): A Selective Protective Group for Amines and Amino Acids. *Org. Lett.* **2023**, *25*, 4103-4107.

[4] SAINT-NT Version 6.0, Madison, Wisconsin, USA: Bruker–AXS, 2001.

[5] SADABS 2008/1, Krause, L.; Herbst-Irmer, R.; Sheldrick, G. M.; Stalke. D. *J. Appl. Crystallogr.* **2015**, *48*, 3-10.

[6] SHELXL 2014/4, Sheldrick, G. M. *Acta Cryst. A* **2015**, *71*, 3-8.

[7] SHELXL 2014/7, Sheldrick, G. M. *Acta Cryst. A* **2015**, *71*, 3-8.

[8] Shmueli, U. ed. International Tables for Crystallography; Springer: New York, 2006.

[9] Macrae, C. F.; Edgington, P. R.; McCabe, P.; Pidcock, E.; Shields, G. P.; Taylor, R.; Towler, M.; Van de Streek, J. J. *J. Appl. Cryst.* **2006**, *39*, 453-457.

[10] Frisch, M. J.; Trucks, G. W.; Schlegel, H. B.; Scuseria, G. E.; Robb, M. A.; Cheeseman, J. R.; Scalmani, G.; Barone, V.; Petersson, G. A.; Nakatsuji, H.; Li, X.; Caricato, M.; Marenich, A. V.; Bloino, J.; Janesko, B. G.; Gomperts, R.; Mennucci, B.; Hratchian, H. P.; Ortiz, J. V.; Izmaylov, A. F.; Sonnenberg, J. L.; Williams; Ding, F.; Lipparini, F.; Egidi, F.; Goings, J.; Peng, B.; Petrone, A.; Henderson, T.; Ranasinghe, D.; Zakrzewski, V. G.; Gao, J.; Rega, N.; Zheng, G.; Liang, W.; Hada, M.; Ehara, M.; Toyota, K.; Fukuda, R.; Hasegawa, J.; Ishida, M.; Nakajima, T.; Honda, Y.; Kitao, O.; Nakai, H.; Vreven, T.; Throssell, K.; Montgomery Jr, J. A.; Peralta, J. E.; Ogliaro, F.; Bearpark, M. J.; Heyd, J. J.; Brothers, E. N.; Kudin, K. N.; Staroverov, V. N.; Keith, T. A.; Kobayashi, R.; Normand, J.; Raghavachari, K.; Rendell, A. P.; Burant, J. C.; Iyengar, S. S.; Tomasi, J.; Cossi, M.; Millam, J. M.; Klene, M.; Adamo, C.; Cammi, R.; Ochterski, J. W.; Martin, R. L.; Morokuma, K.; Farkas, O.; Foresman, J. B.; Fox, D. J. Gaussian 16 Rev. B.01, Wallingford, CT, **2016**.

[11] J.-D. Chai, J.-D.; Head-Gordon, M. *Phys. Chem. Chem. Phys.*, **2008**, *10*, 6615-6620.

[12] Hariharan, P. C.; Pople, J. A. *Mol. Phys.* **1974**, *27*, 209-214; Francl, M. M.; Pietro, W. J.; Hehre, W. J.; Binkley, J. S.; DeFrees, D. J.; Pople, J. A.; Gordon, M. S. *J. Chem. Phys.* **1982**, *77*, 3654-3665; Binning Jr., R. C.; Curtiss, L. A. *J. Comp. Chem.* **1990**, *11* 1206-1216; Clark, T.; Chandrasekhar, J.; Spitznagel, G. W.; Schleyer, P. v. R. *J. Comp. Chem.* **1983**, *4*, 294-301.

[13] Marenich, A. V.; Cramer, C. J.; Truhlar, D. G. *J. Phys. Chem. B* **2009**, *113*, 6378-6396.

[14] Grimme, S. *Chem. Eur. J.* **2012**, *18*, 9955–9964.

[15] https://github.com/patonlab/GoodVibes
